# Tethering Carbohydrates to the Vinyliminium Ligand of Antiproliferative Organometallic Diiron Complexes

**DOI:** 10.1021/acs.organomet.1c00519

**Published:** 2022-02-28

**Authors:** Silvia Schoch, Dalila Iacopini, Maria Dalla Pozza, Sebastiano Di Pietro, Ilaria Degano, Gilles Gasser, Valeria Di Bussolo, Fabio Marchetti

**Affiliations:** † Department of Chemistry and Industrial Chemistry, University of Pisa, 56124 Pisa, Italy; ‡ Chimie ParisTech, PSL University, CNRS, Institute of Chemistry for Life and Health Sciences, 75005 Paris, France; § Department of Pharmacy, University of Pisa, 56126 Pisa, Italy

## Abstract

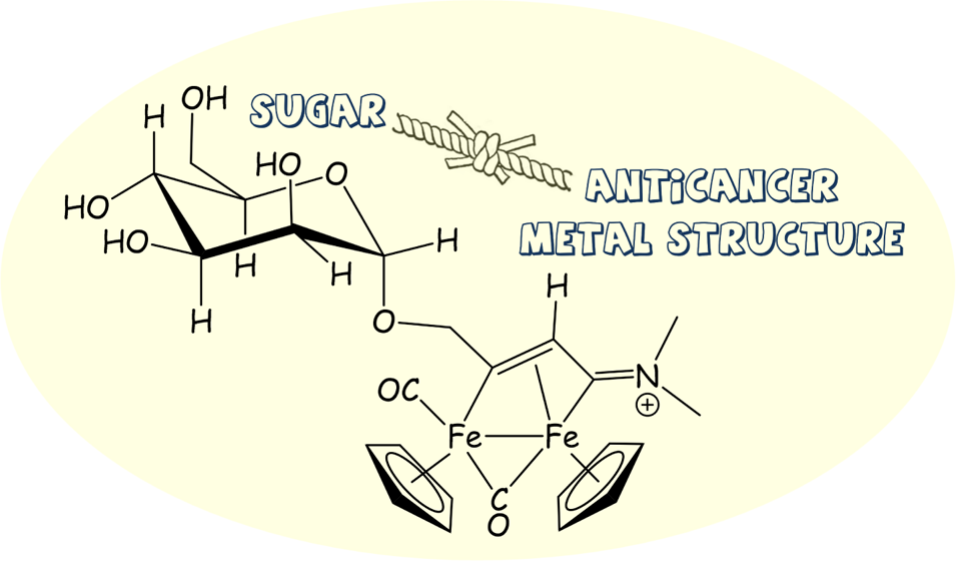

Four propargyl *O*-glycosides derivatized with mannose, glucose, and fructose moieties were synthesized and then incorporated within a diiron structure as part of a vinyliminium ligand. Hence, six glycoconjugated diiron complexes, [**2–5**]CF_3_SO_3_ (see Scheme 1) and the nonglycosylated analogues [**6a–b**]CF_3_SO_3_, were obtained in high yields and unambiguously characterized by elemental analysis, mass spectrometry, and IR and multinuclear NMR spectroscopies. All compounds exhibited a significant stability in DMSO-*d*_6_/D_2_O solution, with 63–89% of the complexes unaltered after 72 h at 37 °C and also in the cell culture medium. The cytotoxicity of [**2**–**6**]CF_3_SO_3_, as well as that of previously reported **7** and **8**, was assessed on CT26 (mouse colon carcinoma), U87 (human glioblastoma), MCF-7 (human breast adenocarcinoma), and RPE-1 (human normal retina pigmented epithelium) cell lines. In general, the IC_50_ values correlate with the hydrophobicity of the compounds (measured as octanol–water partition coefficients) and do not show an appreciable level of selectivity against cancer cells with respect to the nontumor ones.

## Introduction

**Figure 1:**
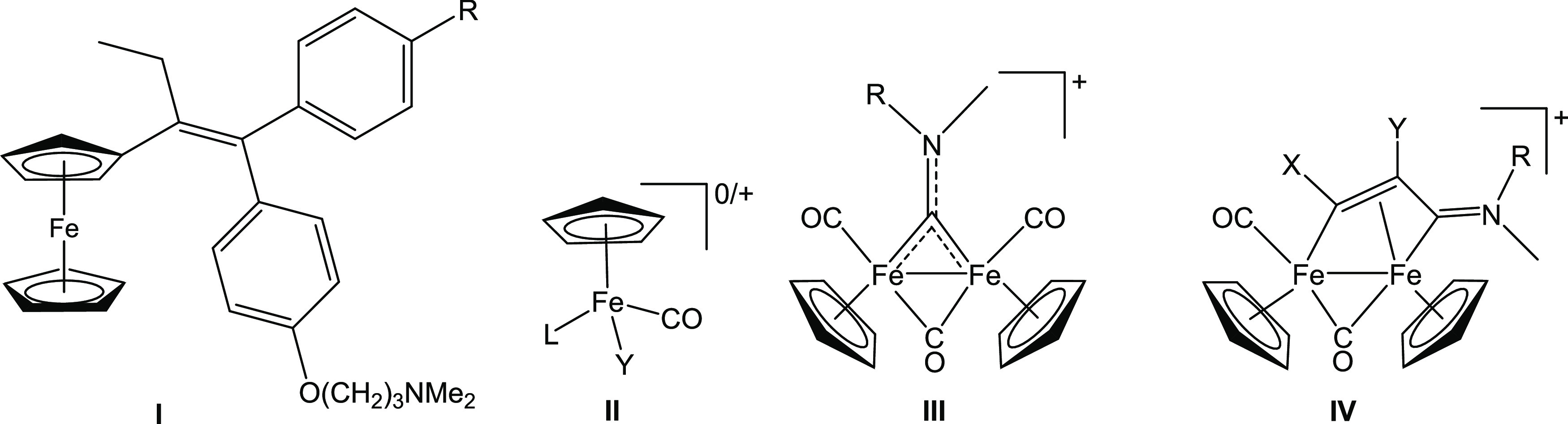
Structures of cyclopentadienyl iron complexes with anticancer activity: (**I**) ferrocifen (R = H, OH); (**II**) piano-stool monoiron complexes (L, Y = CO, phosphine, halide/pseudohalide); diiron complexes with a (**III**) bridging aminocarbyne or (**IV**) vinyliminium ligand (R = alkyl or aryl; R′ = alkyl, aryl, CO_2_Me, 2-thiophenyl, pyridyl; R″ = H, CO_2_Me, Ph, Me; triflate salts).

A wide range of transition-metal complexes have been evaluated for their anticancer properties^1^ with the aim of developing new effective drugs able to overcome the limitations associated with platinum compounds, which are massively administered in the clinic against several types of tumors.^2^ Among the different categories of transition-metal complexes, iron complexes based on the ferrocene scaffold have aroused notable interest in recent years,^3^ and especially, ferrocifens emerged, resulting from the conjugation of the ferrocene skeleton with the drug tamoxifen (Figure 1, structure **I**).^3,4^ The antiproliferative activity of these compounds is ascribable to the redox chemistry of the ferrocenyl iron(II) center, which undergoes oxidation to Fe^III^ in the tumor cells, thus enhancing the formation of toxic metabolites leading to cell death.^5^ Furthermore, “piano-stool” monoiron complexes, containing one cyclopentadienyl moiety and variable coligands (structure **II** in Figure 1), exert in some cases strong in vitro cytotoxicity against tumor cell lines.^6^ Otherwise, the anticancer properties of di-organoiron complexes have been less explored,^7^ despite the fact that a diiron carbonyl core constitutes the active unit of impressively efficient enzymes (i.e., hydrogenases),^8^ in agreement with the general principle that suitable bimetallic systems enable reactivity patterns not accessible in homologous monometallic compounds.^9^ The commercially available [Fe_2_Cp_2_(CO)_4_] (Cp = η^5^-C_5_H_5_) is a convenient entry into diiron organometallic chemistry.^10^ In particular, carbonyl ligands can be sequentially replaced by small molecular pieces, which are assembled, generating unusual bridging hydrocarbyl ligands stabilized by means of multisite coordination.^11^ Thus, cationic μ-aminocarbyne complexes (Figure 1, structure **III**) are accessible by multigram-scale procedures^12^ and represent the starting point to obtain vinyliminium derivatives (structure **IV**) via CO/alkyne substitution, featured by a notable structural variability.^13^ Complexes belonging to the families **III**(14) and **IV**(15) possess a variable antiproliferative activity related to a multitargeted mechanism of action, with prevalent imbalance of cell redox homeostasis.

A general strategy to optimize the activity of anticancer metal complexes consists in the attachment of an organic fragment with documented biological activity to the metal scaffold.^16^ Recently, we applied this approach to obtain diiron vinyliminium complexes **IV** derivatized with aspirin and chlorambucil, showing a clear influence of the bioactive moiety on the cytotoxicity profiles of the resulting complexes.^17^

The selective delivery of metal complexes to a specific kind of cells based on the metabolic features of the latter is a challenging goal, which may be useful for several purposes, including the therapy of pathological states such as cancer. In particular, tumor cells display a high avidity for carbohydrates, especially glucose, to sustain their high proliferation rate, which causes an increased glycolytic activity (Warburg effect).^18^ As a consequence of this significantly increased request of glucose, as energy and bioprecursor sources, cancer cells commonly overexpress glucose transporters (GLUTs) on their cellular membrane surface.^19^ In general, the attachment of carbohydrates to metal structures (either platinum complexes^20^ or not^21^) represents a smart strategy, which potentially exploits GLUT-mediated cell uptake, and carbohydrate–metal complexes generally display enhanced biocompatibility, hydrophilicity (solubility), and pharmacokinetic parameters compared to the nonconjugated counterparts. Other carbohydrates in addition to d-glucose, such as d-mannose and d-fructose as well as OH-protected monosaccharides, can be direct substrates, or their bioprecursors, of GLUT transporters and thus can be considered as candidates for a GLUT-targeting approach.^18b,22^ To date, only a few carbohydrate-containing iron complexes have been proposed as anticancer drug candidates.^23^

Here, we describe the straightforward synthesis of new diiron vinyliminium complexes derivatized with selected glucose, mannose, and fructose units, the evaluation of their behavior in aqueous media, and the assessment of their cytotoxicity toward a panel of cell lines.

## Results and Discussion

### Synthesis and Characterization of Complexes

**Figure 2:**
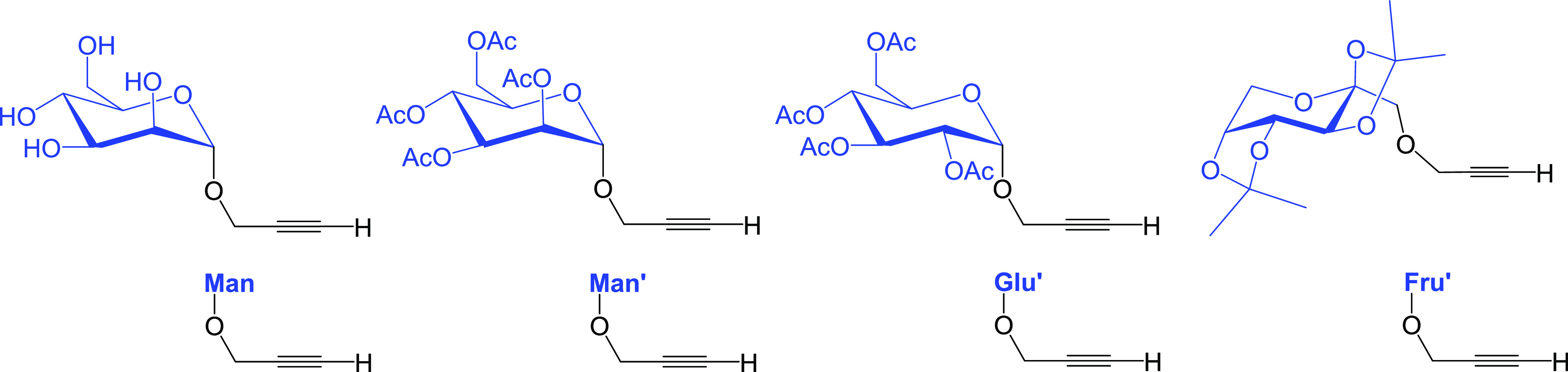
Propargyl *O*-glycosides employed in this work (HC≡CCH_2_OMan and HC≡CCH_2_OMan′ as mannose derivatives; HC≡CCH_2_OGlu′ as glucose derivative; HC≡CCH_2_OFru′ as fructose derivative).

Propargyl *O*-glycosides (Figure 2) were prepared from the corresponding commercially available monosaccharides using optimized literature procedures (see the Supporting Information for details).^24,25^

**Scheme 1:**
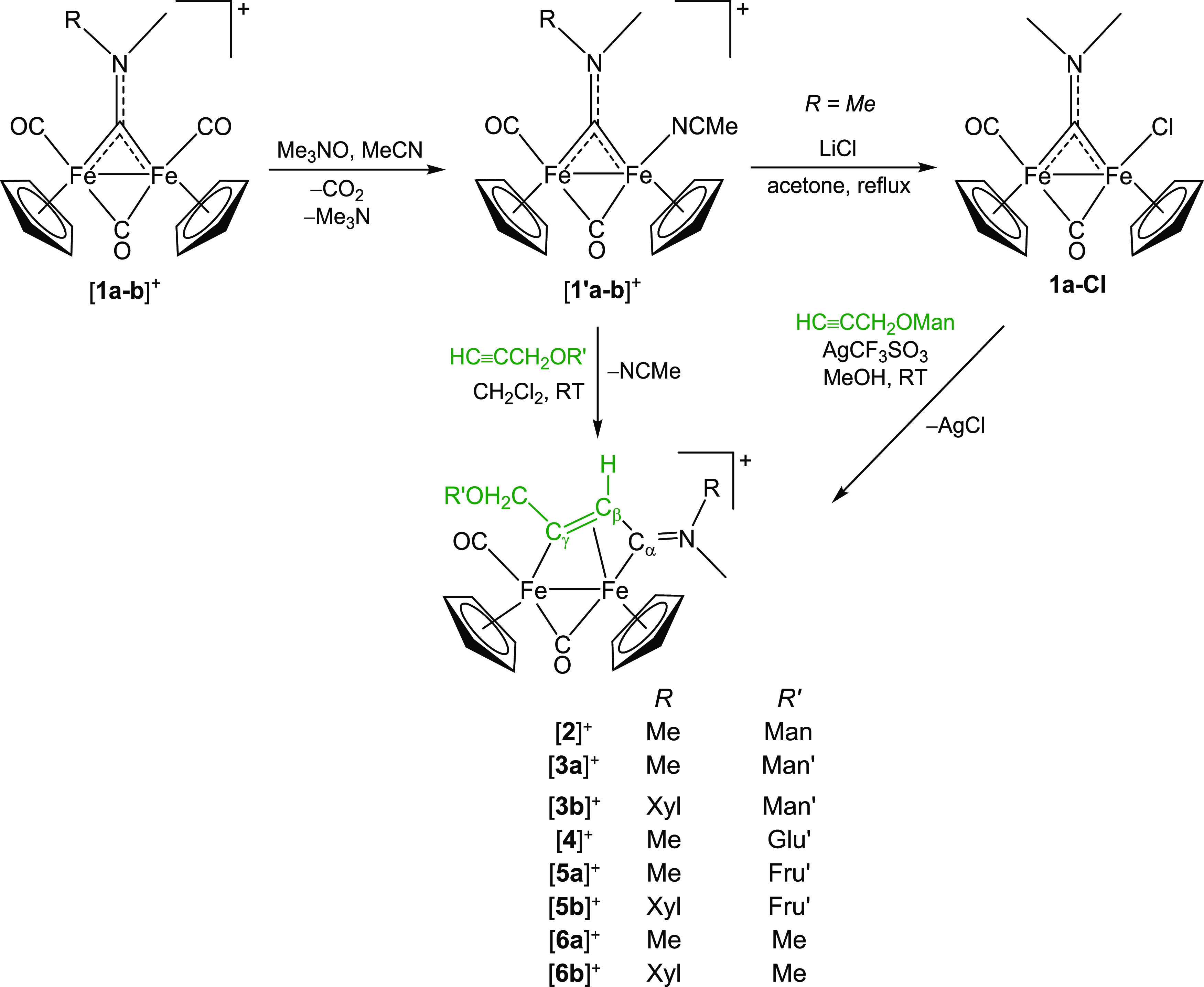
Synthesis of Glycoconjugated Diiron Vinyliminium Complexes (CF_3_SO_3_^–^ Salts) via Coupling of a Bridging Aminocarbyne Ligand with the Alkyne Function Belonging to Carbohydrate-Functionalized Propargyl *O*-Glycosides

Hence, diiron complexes with different carbohydrate-functionalized vinyliminium bridging ligands, [**2–5**]CF_3_SO_3_, were prepared from the easily available aminocarbyne precursors [**1a–b**]CF_3_SO_3_ (Scheme 1). First, one carbonyl ligand is replaced with the relatively labile acetonitrile molecule using the trimethylamine N-oxide strategy to give the adducts [**1**′**a–b**]CF_3_SO_3_ (Scheme 1). The subsequent reaction with the propargyl *O*-glycosides results in acetonitrile displacement by the alkyne function, immediately followed by regiospecific alkyne insertion into the iron–carbyne bond, affording [**2–5**]CF_3_SO_3_. By this method, complex [**2**]CF_3_SO_3_ obtained was impure; its successful preparation was achieved via intermediate acetonitrile/chloride substitution (formation of **1a-Cl**), followed by chloride abstraction with silver triflate in the presence of the alkyne HC≡CCH_2_OMan. Complexes [**6a–b**]CF_3_SO_3_, containing a methyl group in the place of the monosaccharide moiety, were also prepared as reference compounds.

Novel compounds [**2**–**6**]CF_3_SO_3_ were isolated in 85–95% yields after work-up and fully characterized. Mass spectra confirmed the identity of the glycosylated compounds, clearly showing the peak related to the cation.

IR spectra of [**2**–**6**]CF_3_SO_3_ (Figures S9–S17) were recorded in dichloromethane solution except for [**2**]CF_3_SO_3_ (methanol): they share the typical pattern of diiron vinyliminium complexes^13,15,26^ with two intense bands related to the terminal and bridging carbonyl ligands (in the ranges 1989–2002 and 1808–1816 cm^–1^, respectively) and a less intense absorption accounting for the iminium (C_α_–N) bond. The latter is affected mainly by the nature of the iminium substituent R, and it falls at ca. 1680 and 1630 cm^–1^ for R = Me and R = Xyl, respectively. In addition, the spectra of [**3a–b**]CF_3_SO_3_ and [**4**]CF_3_SO_3_ show the band due to the acetyl groups within the carbohydrate fragment around 1750 cm^–1^.

NMR spectra of [**2–5**]CF_3_SO_3_ (in acetone-*d*_6_ or CDCl_3_, Figures S19–S30) revealed the presence of two species in an almost equimolar ratio, and a plausible explanation is given in the following. The formation of the diiron vinyliminium core is not stereoselective, leading to a couple of enantiomers, which were recognized in many crystallographic structures (Figure 3).^13,15,26^ In the present case, the two enantiomers combine with the enantiopure carbohydrate (Figure 2), giving rise to a couple of diastereomers.

**Figure 3:**
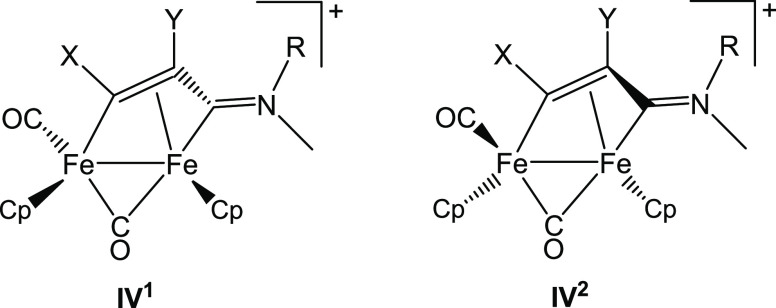
Diiron vinyliminium core is generally obtained as a couple of enantiomers due to the stereogenic iron centers.

Apart from the chirality issue mentioned above, the NMR spectra of [**2**–**6**]CF_3_SO_3_ suggested the highly regio- and stereoselective character of the alkyne insertion reaction. In fact, in the ^1^H NMR spectra, the C_β_–H hydrogen resonates within the interval of 4.5–5.3 ppm, whereas no signals were found at low fields typical for a bridging alkylidene (C_γ_H, >9 ppm).^26,27^ The Cp rings were seen as singlets in the range 5.06–5.74 ppm, which is indicative of a cis arrangement, upon comparison with a library of data available for homologous non glycosylated complexes.^13,15,17,26^ Moreover, the unequal iminium substituents in [**3b**]CF_3_SO_3_ and [**5b**]CF_3_SO_3_ (R = Xyl) adopt the E geometry. Instead, [**6b**]CF_3_SO_3_ exists as a mixture of E and Z isomers (additional Cp resonance at 4.83 ppm), with large prevalence of the former. The diastereotopic proton atoms belonging to the {C_γ_-CH_2_O} unit were detected in the 6.0–6.5 ppm range for [**2**–**5**]CF_3_SO_3_, mostly as a set of three/four signals, in accordance with the presence of two sugar-induced diastereomers. On the other hand, in [**6a–b**]CF_3_SO_3_, two doublets were clearly observed in the 5.5–6.0 ppm range, since the {C_γ_-CH_2_O} hydrogens are diastereotopically anisochronous even in the absence of the enantiopure carbohydrate moiety. In every case, the ^1^H NMR window on the carbohydrate fragment reflects the fully *J*-coupled complexity typical of a pyranosic system: thus, in [**3–5**]CF_3_SO_3_, a series of signals occur in the 4.0–5.5 ppm region, being slightly shielded (3.5–4.5 ppm) in the mannose complex [**2**]CF_3_SO_3_ due to the absence of acetyl protection.

From the ^13^C NMR spectra of [**3–5**]CF_3_SO_3_, the anomeric diagnostic signal can be highlighted in the 95–100 ppm range; as for ^1^H NMR spectra, most of the resonances related to the carbohydrate unit (60–80 ppm range) are doubled because of the pair of diastereomeric complexes. Salient features are represented by the resonances of C_α_ and C_γ_, falling within the intervals of 225.1–233.4, and 199.3–206.9 ppm, respectively. These values account for the (amino)alkylidene nature of C_α_ and the alkylidene nature of C_γ_, coherently with that reported for a vast series of non glycosylated vinyliminium complexes.^13,15^

### Solubility, Stability in Aqueous Solutions, and Octanol–Water Partition Coefficients

Complexes [**2**]CF_3_SO_3_ and [**3a**]CF_3_SO_3_ exhibited the highest water solubility, which could be quantified in D_2_O by ^1^H NMR using dimethylsulfone (Me_2_SO_2_) as a standard (6.1 and 2.0 g·L^–1^, respectively).^28,29^ While [**2**]CF_3_SO_3_ is well soluble in methanol and acetone, it is limitedly soluble in dichloromethane, almost insoluble in chloroform, and insoluble in diethyl ether. Complex [**3a**]CF_3_SO_3_ is well soluble in chlorinated solvents and insoluble in diethyl ether, which facilitated the purification during work-up. The remaining compounds, [**3b-6**]CF_3_SO_3_, were slightly soluble in water, well soluble in dichloromethane and chloroform, and insoluble in diethyl ether.

According to ^1^H NMR spectroscopy (Figures S35–S42), the compounds manifested a substantial stability in D_2_O or D_2_O/DMSO-*d*_6_ solutions (^1^H NMR), with up to 89% of the starting material recovered after 72 h at 37 °C (dimethylsulfone as standard, Table 1). The minor decomposition of the complexes is featured by the precipitation of some solid, while newly formed organometallic species were not detected in solution. Semiquantitative electrospray-ionization mass spectrometry (ESI-MS) analyses suggested that most complexes are quite robust even in the cell culture medium. Briefly, each sample was dissolved in a small volume of DMSO and the solution was diluted with RPMI-1640 medium (final DMSO concentration < 5%). The mixtures were analyzed immediately after preparation and then stored at 37 °C for 72 h in the dark before new analyses. The interpretation of the spectra showed that complexes [**3a–b**]CF_3_SO_3_ and [**4**]CF_3_SO_3_ gradually released one/two protecting groups. In the spectra acquired after 72 h for [**5a–b**]CF_3_SO_3_, bearing the isopropylidene-protected fructose, and [**6a–b**]CF_3_SO_3_, lacking the carbohydrate function, the unaltered complex was the largely prevalent species detected. Interestingly, the hydrophilic and inactive complex [**2**]CF_3_SO_3_ (vide infra) exhibited a distinctive behavior, in that almost immediate degradation was recognized, presumably triggered by some medium component; in this case, the only diiron derivative, which could be detected in solution, albeit in a low concentration, is [**9a**]^+^ (vide infra), resulting from the loss of the carbohydrate moiety. The stability of all complexes, expressed as the percentage of the compound retrieved after 72 h, is detailed in Table 1. According to these outcomes, it appears that the introduction of a nonprotected carbohydrate moiety within the vinyliminium moiety is totally detrimental to the stability of the diiron core; on the other hand, the choice of protected carbohydrates overcomes the stability issues and determines a progressive cleavage of the organometallic scaffold.

**Table 1:** Fraction of the Residual Diiron Complex in the D_2_O/DMSO-*d*_6_ Mixture (2:1 v/v), Determined by ^1^H NMR Spectroscopy (Me_2_SO_2_ as Internal Standard), and in RPMI, Determined by ESI-MS Analysis, after 72 h at 37 °C

compound	stability %	stability RPMI %
**[2]**CF_3_SO_3_	75^a^	0 (0^b^)
**[3a]**CF_3_SO_3_	78^a^	43 (52^b^)
**[3b]**CF_3_SO_3_	83	57 (77^b^)
**[4]**CF_3_SO_3_	69	32 (54^b^)
**[5a]**CF_3_SO_3_	89	78 (78^b^)
**[5b]**CF_3_SO_3_	82	84 (84^b^)
**[6a]**CF_3_SO_3_	78	94 (95^b^)
**[6b]**CF_3_SO_3_	86	97 (97^b^)

a D_2_O solution.

b Total amount of diiron complexes (starting complex + deacetylated derivatives).

Octanol–water partition coefficients (Log *P*_ow_) of the complexes were measured by means of a UV–vis method (see Experimental Studies for details), and the obtained values are reported in Table 2. In general, the diiron complexes display a significant level of hydrophilicity, with [**2**]CF_3_SO_3_ being the most hydrophilic one (Log *P*_ow_ = −0.90). The iminium substituents strongly contribute, and for instance, Log *P*_ow_ for the homologous complexes [**5a**]CF_3_SO_3_ and [**5b**]CF_3_SO_3_ are −0.53 (R = Me) and +0.43 (R = Xyl), respectively. The introduction of the acetylated mannosyl moiety (R′ = Man′, complexes **3a–b**) produces almost the same effect, in terms of hydrophilicity, as the methyl group (R′ = Me, complexes **6a–b**).

**Table 2:** IC_50_ Values (Reported in μM) Obtained after 48 h of Continuous Incubation of Diiron Complexes and Cisplatin with U87, CT26, MCF-7, and RPE1 Cells^a^

compound	CT26	U87	MCF-7	RPE1	Log *P*_ow_
**[2]**CF_3_SO_3_	>100	>100	>100	>100	–0.90 ± 0.06
**[3a]**CF_3_SO_3_	>100	>100	>100	>100	–0.71 ± 0.01
**[3b]**CF_3_SO_3_	20 ± 4	52 ± 15	>100	43 ± 9	–0.12 ± 0.01
**[4]**CF_3_SO_3_	48 ± 5	>100	>100	>100	–0.83 ± 0.01
**[5a]**CF_3_SO_3_	>100	>100	>100	>100	–0.53 ± 0.01
**[5b]**CF_3_SO_3_	6 ± 1	22 ± 3	23 ± 8	26 ± 17	0.43 ± 0.01
**[6a]**CF_3_SO_3_	>100	>100	>100	>100	–0.70 ± 0.01
**[6b]**CF_3_SO_3_	18 ± 8	81 ± 16	29 ± 13	24 ± 4	–0.19 ± 0.01
**[7]**CF_3_SO_3_	7 ± 1	6 ± 1	7 ± 1	8 ± 2	0.4^15a^
**[8]**CF_3_SO_3_	8 ± 1	17 ± 1	28 ± 1	28 ± 2	0.0^15a^
Cisplatin	0.8 ± 0.1	5.9 ± 1.4	19 ± 3	28 ± 4	

a On the right column, Log *P*_ow_ values are reported.

### Cytotoxicity Studies

**Figure 4:**
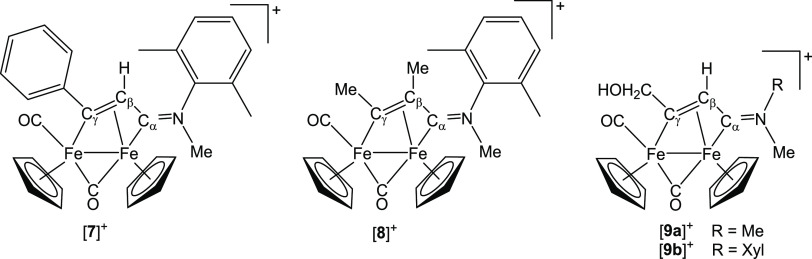
Previously reported diiron vinyliminium complexes analyzed or cited in this work (triflate salts).

The cytotoxicity of the novel diiron complexes [**2**–**6**]CF_3_SO_3_ was evaluated using increasing concentrations of the complexes against the cancer cell lines CT26, U87, and MCF-7 and the nontumoral cell line RPE-1. The concentration of the tested compounds inducing 50% reduction in the cell number compared to control cultures (IC_50_) was determined using the resazurin assay. The previously reported diiron complexes [**7**]CF_3_SO_3_ and [**8**]CF_3_SO_3_^15a^ (Figure 4) and cisplatin were used as references.

The results are compiled in Table 2, while dose–response cell viability curves are supplied as the Supporting Information (Figures S51–S54).

In general, data show a clear correlation between the cytotoxicity and the hydrophobicity of the glycoconjugated compounds and the absence of an appreciable selectivity. Instead, the degree of relative stability of the complexes (Table 1) does not appear to play a prominent role. Thus, [**2**]CF_3_SO_3_, [**3a**]CF_3_SO_3_, [**5a**]CF_3_SO_3_, and [**6a**]CF_3_SO_3_ are not cytotoxic in the concentration range of 0.01–100 μM against all of the tested cell lines, probably due to their substantial hydrophilic character (negative Log *P*_ow_ values), disfavoring cell penetration. The moderate cytotoxicity of [**4**]CF_3_SO_3_ (Log *P*_ow_ = −0.83) against the CT26 cell line emerges as an exception. The behavior of the mannosyl-peracetylated complex [**3b**]CF_3_SO_3_ may be compared with that of the analogous [**6b**]CF_3_SO_3_, lacking the carbohydrate moiety and featuring a close Log *P*_ow_ value. Thus, the two complexes display a comparable activity against the CT26 and U87 cell lines; otherwise, [**6b**]CF_3_SO_3_ is much more active against MCF-7 cells but less selective. On the other hand, nonglycosylated complexes [**7**]CF_3_SO_3_ (Log *P*_ow_ = 0.4) and [**8**]CF_3_SO_3_ (Log *P*_ow_ = 0.0) appear more effective than [**3b**]CF_3_SO_3_ (Log *P*_ow_ = −0.12), [**5b**]CF_3_SO_3_ (Log *P*_ow_ = 0.43), and [**6b**]CF_3_SO_3_ (Log *P*_ow_ = −0.19), suggesting that an appropriate choice of simple substituents on the vinyliminium chain might be more incisive than the incorporation of a carbohydrate moiety. In particular, the cytotoxicity of [**7**]CF_3_SO_3_ exceeds that of cisplatin against the MCF-7 cell line, while comparable IC_50_ values have been recognized for these two compounds on the U87 cell line.

To evaluate if the absence of glucose in the medium could increase or somehow affect the cytotoxicity of the tested compounds, we investigated the difference in terms of IC_50_ between the normal conditions and the cells cultivated in no-glucose medium. In principle, in the latter condition, cells would experience a major demand for glucose (and carbohydrates in general) and may become more prone to internalize the functionalized diiron complexes, resulting in an increased cytotoxicity.^30^ For this study, we selected the moderately active complexes [**3b**]CF_3_SO_3_ and [**5b**]CF_3_SO_3_, containing two different carbohydrate moieties, and [**6b**]CF_3_SO_3_, which is not decorated with any sugar moiety. The collected observations pointed out no different values of IC_50_ comparing the glucose and no-glucose conditions, indicating that the activity of the compounds is not influenced by the absence of glucose (Table 3 and Figure S55). In other words, cell glucose transporters do not seem to be involved in the uptake of the diiron complexes.

**Table 3:** IC_50_ Values (Reported in μM) Obtained after 48 h of Continuous Incubation of Diiron Complexes and Cisplatin with CT26 Cells, Cultivated with and without Glucose, Respectively

compound	with glucose	without glucose
**[3b]**CF_3_SO_3_	18 ± 3	15 ± 3
**[5b]**CF_3_SO_3_	10 ± 4	6.8 ± 0.7
**[6b]**CF_3_SO_3_	21 ± 2	10 ± 3
cisplatin	1.3 ± 0.2	0.7 ± 0.3

**Figure 5:**
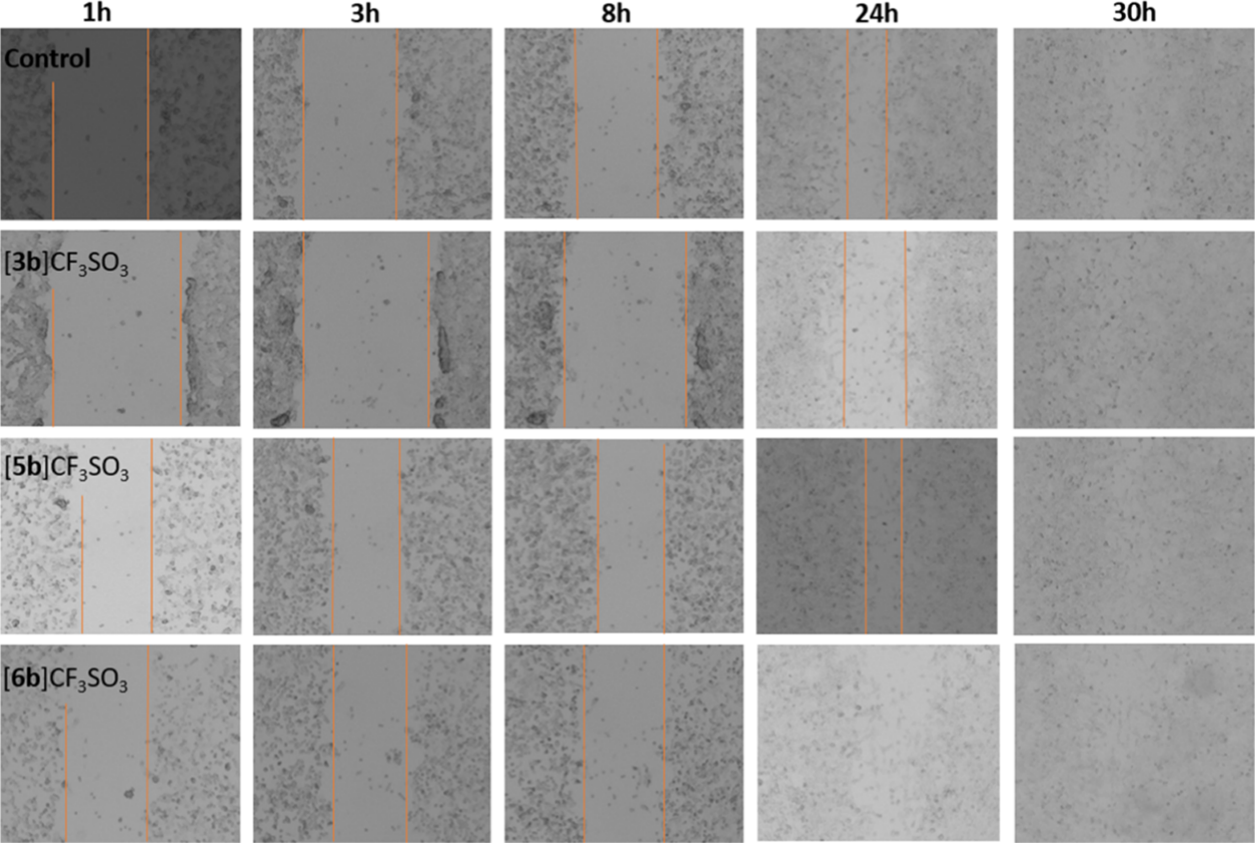
Migration of CT26 cells after 1, 3, 8, 24, and 30 h, following treatment with IC_20_ concentrations of [**3b**]CF_3_SO_3_, [**5b**]CF_3_SO_3_, and [**6b**]CF_3_SO_3_, respectively, or not (control). Orange lines indicate the edges of the scratches. In the experiment, we used less than 1% of DMSO containing Dulbecco’s modified Eagle medium (DMEM) medium. The images are representative from one successive experiment out of three successive individual experiments.

The wound healing assay (also known as the scratch assay) is an economical and simple method to evaluate cell migration *in vitro*, mimicking the migration of cells *in vivo*.^31^ We performed this assay on selected complexes to investigate their cell migration inhibitory potential. First, for each complex, the IC_20_ value (i.e., the concentration of the drug inhibiting 20% of the cell viability) was graphically determined from the respective plot of cell viability (Figures S51–S54). Then, CT26 colon carcinoma cells were treated with [**3b**]CF_3_SO_3_, [**5b**]CF_3_SO_3_, and [**6b**]CF_3_SO_3_ at the respective IC_20_ concentrations. The IC_20_ dose was used for each complex for the evaluation process, to affect the cells but avoiding any other kind of high concentration-dependent effect. After carefully scratching the cellular monolayer, the scratch was monitored to check the differences in the healing between cells treated with diiron complexes and nontreated cells. This qualitative comparison did not reveal a meaningful difference in terms of migration (Figure 5); in fact, the scratch was healed approximately to the same extent over 30 h in the distinct cases. We can conclude that the investigated diiron complexes are not capable of inhibiting the migration of the cells in the conditions used for the assay.

Overall, our findings suggest that diiron vinyliminium complexes [**2–8**]CF_3_SO_3_ exert their cytotoxicity inside the cells, in agreement with the absence of activity detected for the most hydrophilic complexes. The presence of a carbohydrate unit does not seem beneficial to the uptake, and a passive diffusion pathway could be hypothesized for the less hydrophilic complexes, but more studies are required to validate this hypothesis. In agreement with the previous reports, it is presumable that the cytotoxicity is triggered mainly by the intracellular disassembly of the diiron scaffold,^7,15,17^ with the release of iron(I) ions contributing to the imbalance of the cell redox homeostasis.^14,32^ In this regard, the complete inactivity of the highly unstable complex [**2**]CF_3_SO_3_ agrees in that, to supply an antiproliferative effect, the degradation must be operative inside the cell. The slightly lower performance exhibited by the relatively lipophilic carbohydrate complexes, compared to the nonfunctionalized ones [**7**–**8**]CF_3_SO_3_, might be related to some interference of the carbohydrate function with degradation routes, which appear essential to the drug activity (see above). In addition, the possible cleavage of the glycosidic bond inside the cell would lead to vinyliminium derivatives containing a {CH_2_OH} function; in this regard, it has to be noted that complexes [**9a**–**b**]^+^ (Figure 4), which would be generated by this process from [**3**]^+^ and [**5**]^+^, respectively, were previously found to be considerably less active and less selective than the related complexes with other C_γ_ substituents.^15a^

## Conclusions

Conjugation with carbohydrates is a well-established strategy to improve anticancer activity of transition-metal complexes, essentially aimed at increasing the drug uptake by cancer cells. Here, we report the incorporation of alkynes functionalized with different monosaccharide moieties within a di-organoiron scaffold, which was previously demonstrated to exert promising in vitro cytotoxicity. Antiproliferative activities of the new complexes on a panel of cancer cell lines correlate with their lipophilicity, ranging from moderate to inactive and showing an absence of appreciable selectivity with respect to a nontumoral cell line. On the other hand, analogous diiron complexes with different substituents on the bridging vinyliminium ligand, analyzed as references, performed better in the same conditions, thus confirming the potential of the present category of organometallics in the medicinal field. The absence of a clear favorable effect of the carbohydrate moiety may be a consequence of adverse steric factors, disfavoring the interaction of the encumbered diiron scaffold with GLUT transporters and thus hampering the transport of the complexes through the cell membrane.^18b^

However, the versatility of the diiron structure and the very general character of the alkyne insertion reaction affording vinyliminium ligands, demonstrated also in the present work, may constitute a notable potential for the design and future development of optimal iron drug candidates.

## Experimental Studies

### Synthesis and Characterization of Compounds

#### General Details

All operations were conducted in air, unless otherwise specified. Once isolated, all of the products were stored in air, except the hygroscopic complex [**2**]CF_3_SO_3_, which was stored under N_2_. Organic reactants were purchased from TCI Europe or Merck and were of the highest purity available, while solvents were purchased from Merck (petroleum ether, bp = 40–60 °C). The synthesis and characterization of propargyl *O*-glycosides are provided as the Supporting Information. Complexes [Fe_2_Cp_2_(CO)_2_(μ-CO){μ-CNMe(R)}]CF_3_SO_3_ (R = Me, [**1a**]CF_3_SO_3_; R = Xyl = 2,6-C_6_H_3_Me_2_, [**1b**]CF_3_SO_3_),^12^ [Fe_2_Cp_2_(CO)(μ-CO){μ-η^1^:η^3^-C_γ_(Ph)C_β_HC_α_N(Me)(Xyl)}]CF_3_SO_3_ (**7**),^15a^ and [Fe_2_Cp_2_(CO)(μ-CO){μ-η^1^:η^3^-C_γ_(Me)C_β_(Me)C_α_N(Me)(Xyl)}]CF_3_SO_3_ (**8**)^15a^ were prepared according to the respective literature procedures. Separations were carried out on columns of silica (Merck), deactivated alumina (Merck, 4% w/w water), or celite (Fluka, 512 Medium). Infrared spectra of solutions were recorded on a PerkinElmer Spectrum 100 FT-IR spectrometer with a CaF_2_ liquid transmission cell (2300–1500 cm^–1^ range) or on solid samples at 298 K on a PerkinElmer FT-IR spectrometer, equipped with a UATR sampling accessory. UV–vis spectra were recorded on an Ultraspec 2100 Pro spectrophotometer. IR and UV–vis spectra were processed with Spectragryph software.^33^ NMR spectra were recorded at 298 K on a Bruker Avance II DRX400 instrument equipped with a BBFO broadband probe. Chemical shifts (expressed in parts per million) are referenced to the residual solvent peaks (^1^H, ^13^C).^34^ NMR spectra were assigned with the assistance of ^1^H–^13^C (*gs*-HSQC and *gs*-HMBC) correlation experiments.^35^ NMR signals due to secondary isomeric forms (where it has been possible to detect them) are italicized. Elemental analyses were performed on a Vario MICRO cube instrument (Elementar). Electrospray-ionisation quadrupole time-of-flight (ESI-Q-ToF) flow injection analyses (FIA) were carried out using a 1200 Infinity HPLC (Agilent Technologies), coupled to a Jet Stream ESI interface (Agilent) with a quadrupole-time of flight tandem mass spectrometer 6530 Infinity Q-TOF (Agilent Technologies). High-performance liquid chromatography-mass spectrometry (HPLC-MS) grade acetonitrile was used as the mobile phase (Carlo Erba, Italy). The flow rate was 0.2 mL min^–1^ (total run time 3 min). The ESI operating conditions were: drying gas (N_2_, purity > 98%): 350 °C and 10 L·min^–1^; capillary voltage: 4.5 kV; nozzle voltage: 1 kV; nebulizer gas: 35 psig; sheath gas (N_2_, purity > 98%): 375 °C and 11 L min^–1^. The fragmentor was kept at 50 V, the skimmer at 65 V, and the OCT 1 RF at 750 V. High-resolution ESI-MS spectra were achieved in positive mode in the range 100–1700 *m*/*z*; the mass axis was calibrated daily using the Agilent tuning mix HP0321 (Agilent Technologies) prepared in acetonitrile and water.

### Synthesis and Characterization of [Fe_2_Cp_2_(Cl)(CO)(μ-CO){μ-CNMe_2_}], 1a-Cl (Figure 6)

**Figure 6:**
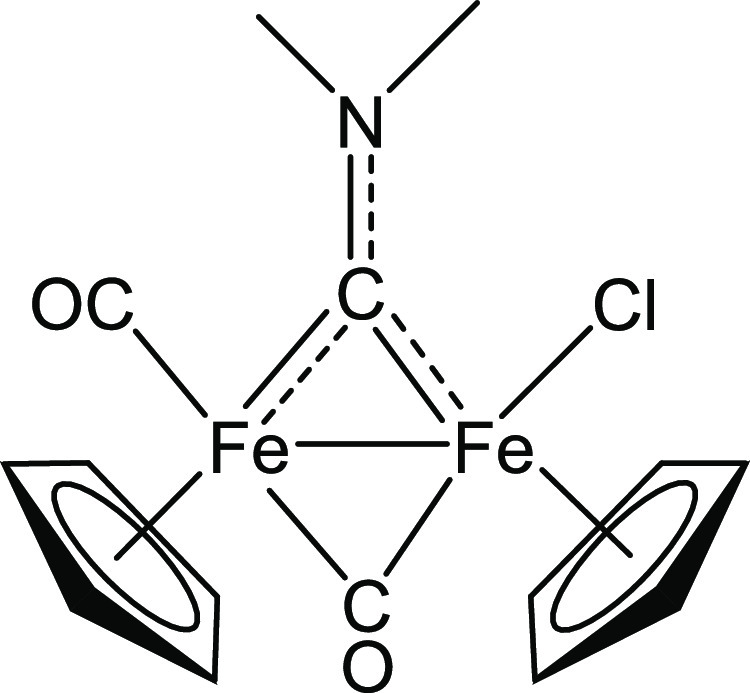
Structure of **1a-Cl**.

The title compound was prepared using a modified literature procedure.^36^ A solution of [**1a**]CF_3_SO_3_ (1.02 g, 1.92 mmol) in acetonitrile (15 mL) was treated with Me_3_NO (188 mg, 2.50 mmol), and the resulting solution was stirred for 2 h, enabling the release of produced gas (CO_2_, Me_3_N). The complete conversion of [**1a**]CF_3_SO_3_ into the acetonitrile adduct [**1**′**a**]CF_3_SO_3_^36^ was checked by IR spectroscopy. The volatiles were eliminated under reduced pressure, affording a dark-brown residue, which was dissolved into acetone (30 mL). Lithium chloride (132 mg, 3.11 mmol) was added, and the resulting mixture was heated at reflux for 2 h. The complete conversion of the acetonitrile adduct into **1a-Cl** was checked by IR spectroscopy in CH_2_Cl_2_ solution. After removal of the solvent under reduced pressure, the residue was dissolved in dichloromethane and filtered on a celite pad under N_2_ atmosphere. The solvent removal under vacuum led to recover the title compound as a light-brown solid. Yield 559 mg (75%). Anal. calcd for C_15_H_16_ClFe_2_NO_2_: C, 46.26; H, 4.14; N, 3.60. Found: C, 46.35; H, 4.16; N, 3.48. IR (CH_2_Cl_2_): υ̃/cm^–1^ = 1978vs (CO), 1800s (μ-CO), 1575m (μ-CN). ^1^H NMR (CDCl_3_): δ/ppm = 4.76, 4.68 (s, 10 H, Cp); 4.73, 4.28 (s, 6H, NMe_2_) (Figure 6).

### Synthesis and Characterization of Diiron Vinyliminium Complexes

#### [Fe_2_Cp_2_(CO)(μ-CO){μ-η^1^:η^3^-C_γ_(CH_2_O-α-Mannopyranosyl)C_β_HC_α_NMe_2_}]CF_3_SO_3_, [2]CF_3_SO_3_ (Figure 7)

**Figure 7:**
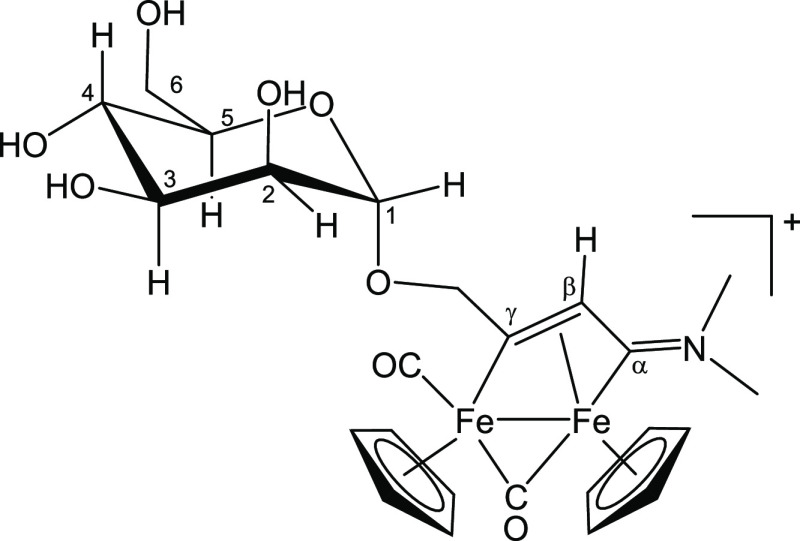
Structure of [**2**]^+^.

A mixture of **1a-Cl** (128 mg, 0.33 mmol) and HC≡CCH_2_OMan (72 mg, 0.33 mmol), in methanol (20 mL), was treated with AgCF_3_SO_3_ (86 mg, 0.33 mmol). The resulting mixture was stirred at room temperature for 70 min and then filtered on a celite pad to remove AgCl. The filtered solution was dried under reduced pressure. The obtained black residue was repeatedly washed with CHCl_3_ and then evaporation of the solvent under reduced pressure afforded [**2**]CF_3_SO_3_ as a hygroscopic black solid. This solid was dissolved in MeOH (2 mL) under N_2_ atmosphere and quickly precipitated by adding petroleum ether (25 mL). A black powder was isolated upon evaporation of the solvent under vacuum and then stored under N_2_. Yield 212 mg (89%). Anal. calcd for C_25_H_30_F_3_Fe_2_NO_11_S: C, 41.63; H, 4.19; N, 1.94. Found: C, 41.24; H, 4.29; N, 1.82. HR-ESI-MS: [M]^+^*m*/*z* = 572.0663 (theoretical for [C_24_H_30_Fe_2_NO_8_]^+^: *m*/*z* = 572.0670; error: −1.2 ppm). IR (CH_3_OH): υ̃/cm^–1^ = 1989vs (CO), 1813s (μ-CO), 1688m (C_α_N). ^1^H NMR (acetone-*d*_6_): δ/ppm = 6.40–5.88 (m, 2 H, C_γ_CH_2_); 5.55, 5.22 (s, 10 H, Cp); 5.52 (m, 1 H, H^1^); 5.29, 4.30–3.80 (m, 6 H, H^2^ + H^3^ + H^4^ + H^5^ + H^6^); 5.25 (s, 1 H, C_β_H); 3.97, 3.37 (s, 6 H, NMe_2_); 3.85–3.58 (s, 4 H, OH). Diastereomeric ratio = 1. ^13^C{^1^H} NMR (acetone-*d*_6_): δ/ppm = 256.1 (μ-CO); 225.5 (C_α_); 210.4 (CO); 201.3 (C_γ_); 121.0 (q, ^1^*J*_C–F_ = 321 Hz, CF_3_); 101.0, 100.0 (C^1^); 89.8, 87.5 (Cp); 88.5, 86.6, 74.1, 73.9, 71.7, 71.5, 71.0, 70.7 (C^2^ + C^3^ + C^4^ + C^5^); 80.3, 79.7 (C_γ_*C*H_2_); 61.7, 61.5 (C^6^); 50.7, 44.4 (NMe_2_); 47.3 (C_β_) (Figure 7).

### General Procedure for the Synthesis of [**3**–**5**]CF_3_SO_3_

A solution of [**1a**–**b**]CF_3_SO_3_ in MeCN (ca. 10 mL) was treated with Me_3_NO (ca. 1.2 equiv). The resulting mixture was stirred for 50 min and progressive color darkening was observed. The complete conversion of the starting material into the corresponding acetonitrile adduct [**1**′**a**–**b**]CF_3_SO_3_^36^ was checked by IR spectroscopy. The volatiles were removed under vacuum to afford a dark-brown residue, which was dissolved in dichloromethane and treated with the selected alkyne. This solution was stirred at room temperature for 4 days, and then it was filtered through celite. The volatiles were evaporated from the filtered solution under reduced pressure; thus, the residue was repeatedly washed with diethyl ether and finally dried under vacuum. The synthesis of [**2**]CF_3_SO_3_ using this procedure (from [**1a**]CF_3_SO_3_) afforded the unclean product in ca. 72% yield.

#### [Fe_2_Cp_2_(CO)(μ-CO){μ-η^1^:η^3^-C_γ_(CH_2_O-2,3,4,6-Tetra-*O*-acetyl-α-mannopyranosyl)C_β_HC_α_NMe_2_}]CF_3_SO_3_, [**3a**]CF_3_SO_3_ (Figure 8)

**Figure 8:**
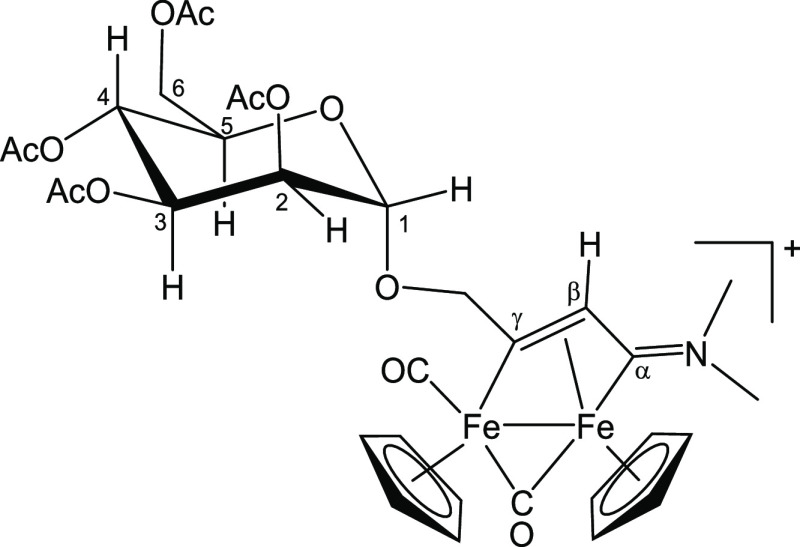
Structure of [**3a**]^+^.

From [**1**′**a**]CF_3_SO_3_, freshly prepared from [**1a**]CF_3_SO_3_ (91 mg, 0.17 mmol) and HC≡CCH_2_OMan′ (91 mg, 0.24 mmol). Brown solid, yield 133 mg (87%). Anal. calcd for C_33_H_38_F_3_Fe_2_NO_15_S: C, 44.56; H, 4.31; N, 1.57. Found: C, 44.38; H, 4.39; N, 1.70. HR-ESI-MS: [M]^+^*m*/*z* = 740.1094 (theoretical for [C_32_H_38_Fe_2_NO_12_]^+^: *m*/*z* = 740.1093; error: 0.1 ppm). IR (CH_2_Cl_2_): υ̃/cm^–1^ = 1992s (CO), 1811m (μ-CO), 1750vs (C=O), 1680w (C_α_N). ^1^H NMR (acetone-*d*_6_): δ/ppm = 6.50–6.00 (m, 2 H, C_γ_CH_2_); 5.58, 5.58, 5.27, 5.26 (s, 10 H, Cp); 5.55–5.38, 5.19, 4.41 (m, 5 H, H^1^ + H^2^ + H^3^ – H^4^ + H^5^); 5.24 (s, 1 H, C_β_H); 4.30, 4.28 (m, 2 H, H^6^); 3.99, 3.38, 3.38 (s, 6 H, NMe_2_); 2.20–1.95 (s, 12 H, 4× O=CMe−). Diastereomeric ratio = 1. ^13^C{^1^H} NMR (acetone-*d*_6_): δ/ppm = 255.3, 255.2 (μ-CO); 225.3 (C_α_); 210.3, 210.2 (CO); 199.7, 199.3 (C_γ_); 170.1, 169.8, 169.4 (4× O=*C*Me); 98.2, 97.3 (C^1^); 89.8, 87.5, 87.5 (Cp); 80.1 (C_γ_*C*H_2_); 69.4, 69.3 (C^3^ + C^4^ + C^5^); 65.9 (C^2^); 62.6, 62.4 (C^6^); 50.7, 44.4 (NMe_2_); 47.6 (C_β_); 19.9 (4× O=C*Me*) (Figure 8).

#### [Fe_2_Cp_2_(CO)(μ-CO){μ-η^1^:η^3^-C_γ_(CH_2_O-2,3,4,6-Tetra-*O*-acetyl-α-mannopyranosyl)C_β_HC_α_N(Me)(Xyl)}]CF_3_SO_3_, [**3b**]CF_3_SO_3_ (Figure 9)

**Figure 9:**
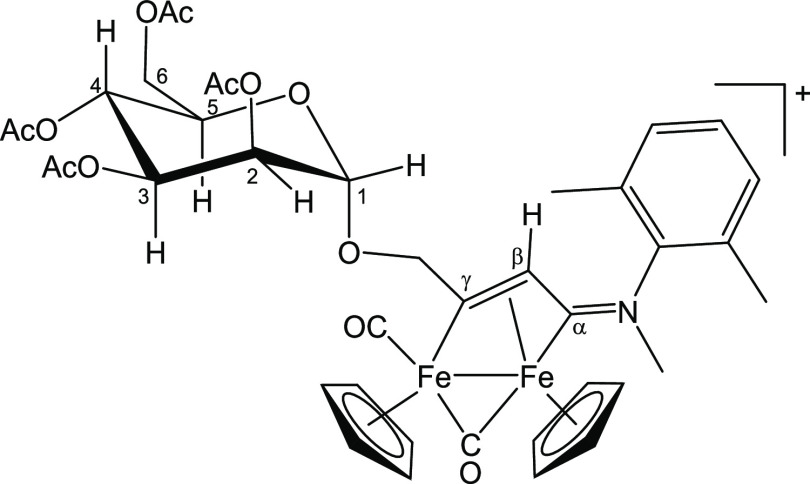
Structure of [**3b**]^+^.

From [**1**′**b**]CF_3_SO_3_, freshly prepared from [**1b**]CF_3_SO_3_ (106 mg, 0.17 mmol) and HC≡CCH_2_OMan′ (120 mg, 0.31 mmol). Brown solid, yield 152 mg (91%). Anal. calcd for C_40_H_44_F_3_Fe_2_NO_15_S: C, 49.05; H, 4.53; N, 1.43. Found: C, 48.80; H, 4.67; N, 1.53. HR-ESI-MS: [M]^+^*m*/*z* = 830.1561 (theoretical for [C_39_H_44_Fe_2_NO_12_]^+^: *m*/*z* = 830.1562; error: −0.1 ppm). IR (CH_2_Cl_2_): υ̃/cm^–1^ = 2002s (CO), 1816s (μ-CO), 1751vs (C=O), 1633m (C_α_N). ^1^H NMR (acetone-*d*_6_): δ/ppm = 7.30–7.20, 7.08 (m, 3 H, C_6_H_3_); 6.38, 6.29, 6.15, 5.97 (d, ^2^*J*_HH_ = 15.0 Hz, 2 H, C_γ_CH_2_); 5.74, 5.73, 5.48, 5.48 (s, 10 H, Cp); 5.69 (m, 1 H, H^1^); 5.44–5.30 (m, 4 H, H^2^ + H^3^ + H^4^ + H^5^); 4.38 (s, 3 H, NMe); 4.27–4.10 (m, 2 H, H^6^); 4.15 (s, 1 H, C_β_H); 2.39, 2.36, 1.87, 1.86 (s, 6 H, C_6_H_3_*Me*_2_); 2.16, 2.15, 2.10, 2.09, 2.03, 2.03, 2.02, 2.01 (s, 12 H, 4× O=CMe). Diastereomeric ratio = 1.2. ^13^C{^1^H} NMR (acetone-*d*_6_): δ/ppm = 253.2, 253.0 (μ-CO); 232.7, 232.6 (C_α_); 210.2, 210.1 (CO); 203.9, 203.5 (C_γ_); 170.0, 169.9, 169.8, 169.8, 169.8, 169.6, 169.2 (4× O=*C*Me); 145.2, 145.2, 131.9, 131.3, 131.3 (*ipso-*C_6_H_3_); 129.6, 129.5, 129.4, 129.3, 129.2 (C_6_H_3_); 98.1, 97.1 (C^1^); 90.6, 87.9, 87.8 (Cp); 80.5, 79.9 (C_γ_*C*H_2_); 69.5, 69.4, 69.3, 69.3, 69.2, 69.1 (C^3^ + C^4^ + C^5^); 65.7, 65.6 (C^2^); 62.3, 62.1 (C^6^); 54.1 (C_β_); 45.8, 45.8 (NMe); 19.9–19.7 (4× O=C*Me*); 17.1, 17.1, 16.6 (C_6_H_3_*Me*_*2*_) (Figure 9).

#### [Fe_2_Cp_2_(CO)(μ-CO){μ-η^1^:η^3^-C_γ_(CH_2_O-2,3,4,6-Tetra-*O*-acetyl-α-glucopyranosyl)C_β_HC_α_NMe_2_}] CF_3_SO_3_, [**4**]CF_3_SO_3_ (Figure 10)

**Figure 10:**
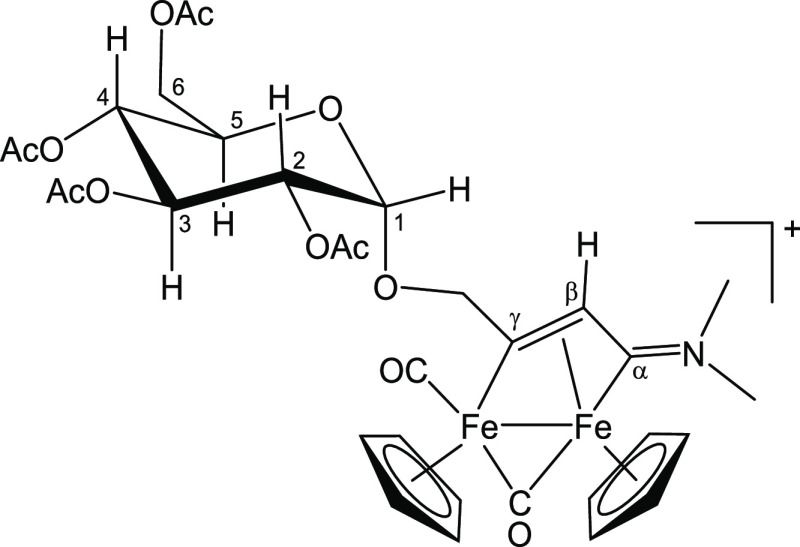
Structure of [**4**]^+^.

From [**1**′**a**]CF_3_SO_3_, freshly prepared from [**1a**]CF_3_SO_3_ (69 mg, 0.13 mmol) and HC≡CCH_2_OGlu′ (74 mg, 0.19 mmol). Dark-brown solid, yield 99 mg (85%). Anal. calcd for C_33_H_38_F_3_Fe_2_NO_15_S: C, 44.56; H, 4.31; N, 1.57. Found: C, 44.68; H, 4.22; N, 1.65. IR (CH_2_Cl_2_): υ̃/cm^–1^ = 1993m (CO), 1811m (μ-CO), 1753vs (C=O), 1682w (C_α_N). HR-ESI-MS: [M]^+^*m*/*z* = 740.1093 (theoretical for [C_32_H_38_Fe_2_NO_12_]^+^: *m*/*z* = 740.1093; error: 0.0 ppm). ^1^H NMR (acetone-*d*_6_): δ/ppm = 6.44–5.95 (m, 2 H, C_γ_CH_2_); 5.66–5.59, 5.24–5.16 (m, 3 H, H^1^ + H^3^ + H^4^); 5.59, 5.56, 5.30, 5.28 (s, 10 H, Cp); 5.20 (s, 1 H, C_β_H); 5.12 (dt, ^3^*J*_H5–H4_ = 10.3 Hz, ^3^*J*_H5–H6_ = 3.3 Hz, 1 H, H^5^); 4.47 (m, 1 H, H^2^); 4.35–4.22 (m, 2 H, H^6^); 3.99, 3.40, 3.38 (s, 6 H, NMe_2_); 2.10–1.97 (s, 12 H, 4× O=CMe). Diastereomeric ratio = 1. ^13^C{^1^H} NMR (acetone-*d*_6_): δ/ppm = 255.0 (μ-CO); 225.2 (C_α_); 210.2 (CO); 200.0 (C_γ_); 169.7, 169.5, 169.4, 169.1 (4× O=*C*Me); 96.8, 95.6 (C^1^); 89.8, 89.7, 87.6, 87.4 (Cp); 80.5, 79.8 (C_γ_*C*H_2_); 70.9, 70.6 (C^5^); 70.0, 69.9, 68.7 (C^3^ + C^4^); 68.1, 68.1 (C^2^); 62.1, 61.9 (C^6^); 50.7, 44.4 (NMe_2_); 47.7, 47.1 (C_β_); 19.9, 19.8, 19.7 (4× O=C*Me*) (Figure 10).

#### [Fe_2_Cp_2_(CO)(μ-CO){μ-η^1^:η^3^-C_γ_(CH_2_O-2,3:4,5-Di-*O*-isopropylidene-)-β-d-fructopyranosyl)C_β_HC_α_NMe_2_}]CF_3_SO_3_, [**5a**]CF_3_SO_3_ (Figure 11)

**Figure 11:**
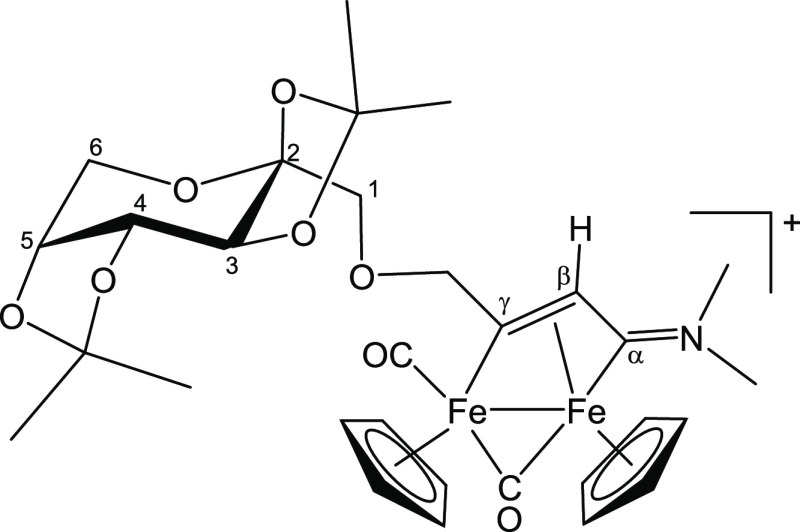
Structure of [**5a**]^+^.

From [**1**′**a**]CF_3_SO_3_, freshly prepared from [**1a**]CF_3_SO_3_ (218 mg, 0.41 mmol) and HC≡CCH_2_OFru′ (147 mg, 0.49 mmol). Brown solid, yield 284 mg (86%). Anal. calcd for C_31_H_38_F_3_Fe_2_NO_11_S: C, 46.46; H, 4.78; N, 1.75. Found: C, 46.32; H, 4.86; N, 1.70. HR-ESI-MS: [M]^+^*m*/*z* = 652.1286 (theoretical for [C_30_H_38_Fe_2_NO_8_]^+^: *m*/*z* = 652.1296; error: −1.5 ppm). IR (CH_2_Cl_2_): υ̃/cm^–1^ = 1991m (CO), 1810m (μ-CO), 1681w (C_α_N). δ/ppm = 5.80–5.75 (m, 2 H, C_γ_CH_2_); 5.24, 5.24, 5.07, 5.06 (s, 10 H, Cp); 5.10, 5.03 (s, 1 H, C_β_H); 4.69 (m, 1 H, H^4^); 4.42 (m, 1 H, H^3^); 4.30 (m, 1 H, H^5^); 4.10–3.95 (m, 2 H, H^6^); 3.98–3.93, 3.84–3.77 (m, 2 H, H^1^); 3.88, 3.30, 3.30 (s, 6 H, NMe_2_); 1.60, 1.59, 1.56, 1.53, 1.51, 1.40, 1.38 (s, 12 H, 2× CMe_2_). Diastereomeric ratio = 1. ^13^C{^1^H} NMR (CDCl_3_): δ/ppm = 256.2, 256.1 (μ-CO); 225.3, 225.1 (C_α_); 209.8, 209.8 (CO); 201.7, 201.5 (C_γ_); 109.1, 108.9, 108.8 (2× *C*Me_2_); 102.6 (C^2^); 89.4, 87.6 (Cp); 85.0, 84.9 (C_γ_*C*H_2_); 73.8, 73.6 (C^6^); 71.0, 70.8, 70.7, 70.1 (C^3^ + C^4^ + C^5^); 61.2 (C^1^); 51.4, 44.9 (NMe_2_); 50.1, 49.7 (C_β_); 26.5, 26.1, 25.6, 24.1 (2× C*Me*_2_) (Figure 11).

#### [Fe_2_Cp_2_(CO)(μ-CO){μ-η^1^:η^3^-C_γ_(CH_2_O-2,3:4,5-Di-*O*-isopropylidene-)-β-d-fructopyranosyl)C_β_HC_α_N(Me)(Xyl)}]CF_3_SO_3_, [**5b**]CF_3_SO_3_ (Figure 12)

**Figure 12:**
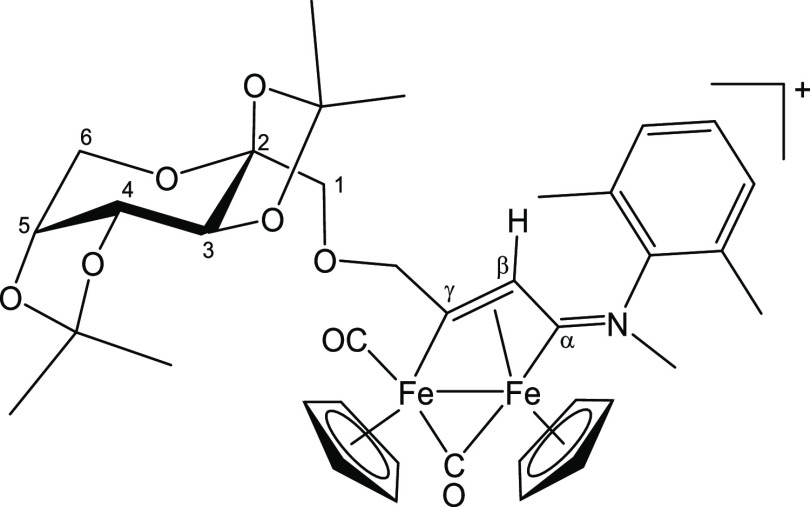
Structure of [**5b**]^+^.

From [**1**′**b**]CF_3_SO_3_, freshly prepared from [**1b**]CF_3_SO_3_ (229 mg, 0.37 mmol) and HC≡CCH_2_OFru′ (135 mg, 0.45 mmol). Brown solid, yield 280 mg (85%). Anal. calcd for C_38_H_44_F_3_Fe_2_NO_11_S: C, 51.19; H, 4.97; N, 1.57. Found: C, 50.94; H, 5.03; N, 1.47. HR-ESI-MS: [M]^+^*m*/*z* = 742.1759 (theoretical for [C_37_H_44_Fe_2_NO_8_]^+^: *m*/*z* = 742.1766; error: −0.9 ppm). IR (CH_2_Cl_2_): υ̃/cm^–1^ = 2002vs (CO), 1815s (μ-CO), 1633m (C_α_N). ^1^H NMR (CDCl_3_): δ/ppm = 7.18, 7.10, 6.95 (m, 3 H, C_6_H_3_); 6.00–5.70 (m, 2 H, C_γ_CH_2_); 5.44, 5.21, 5.20 (s, 10 H, Cp); 4.71, 4.66 (s, 1 H, C_β_H); 4.63 (m, 1 H, H^4^); 4.30, 4.27 (m, 2 H, H^3^ + H^5^); 4.00–3.85 (m, 2 H, H^6^); 3.95–3.85, 3.75 (m, 2 H, H^1^); 4.20 (s, 3 H, NMe); 2.28, 2.27, 1.77 (s, 6 H, C_6_H_3_*Me*_2_); 1.55, 1.54, 1.49, 1.45, 1.37, 1.36, 1.35, 1.31 (s, 12 H, 2× CMe_2_). Diastereomeric ratio = 1. ^13^C{^1^H} NMR (CDCl_3_): δ/ppm = 255.8, 253.9 (μ-CO); 232.9 (C_α_); 209.9, 209.6 (CO); 205.8, 205.6 (C_γ_); 145.9, 144.9, 131.4, 131.1 (*ipso-*C_6_H_3_); 129.6, 129.4, 129.3 (C_6_H_3_); 109.1, 108.6 (2× *C*Me_2_); 102.6 (C^2^); 90.3, 87.9 (Cp); 85.1, 84.7 (C_γ_*C*H_2_); 74.1, 73.5 (C^6^); 70.9, 70.7, 70.1 (C^3^ + C^4^ + C^5^); 61.1 (C^1^); 48.8, 48.0 (C_β_); 46.2, 46.1 (NMe); 26.5, 26.0, 25.6, 24.0 (2× C*Me*_2_); 17.9, 17.2 (C_6_H_3_*Me*_*2*_) (Figure 12).

### General Procedure for the Synthesis of [**6a**–**b**]CF_3_SO_3_

A solution of [**1a–b**]CF_3_SO_3_ (ca. 0.5 mmol) in MeCN (ca. 10 mL) was treated with Me_3_NO (ca. 1.2 equiv). The resulting mixture was stirred for 50 min, and progressive color darkening was observed. The complete conversion of the starting material into the corresponding acetonitrile adduct [**1**′**a–b**]CF_3_SO_3_^36^ was checked by IR spectroscopy. The volatiles were removed under vacuum to afford a dark-brown residue, which was dissolved in dichloromethane and treated with methyl propargyl ether. This solution was stirred at room temperature for 3 days, then it was charged on an alumina column. Elution with CH_2_Cl_2_/tetrahydrofuran (THF) mixtures allowed separation of the unreacted alkyne and impurities, and hence a brown band was collected with methanol. After removal of the solvent, the residue was dissolved in dichloromethane and filtered on a short celite pad. Evaporation of the solvent under vacuum afforded the product as a hygroscopic solid material.

#### [Fe_2_Cp_2_(CO)(μ-CO){μ-η^1^:η^3^-C_γ_(CH_2_OMe)C_β_HC_α_NMe_2_}]CF_3_SO_3_, [**6a**]CF_3_SO_3_ (Figure 13)

**Figure 13:**
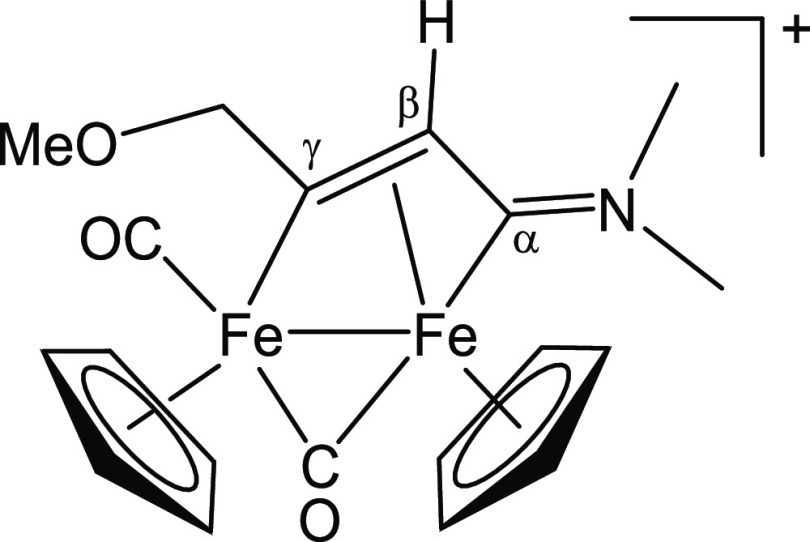
Structure of [**6a**]^+^.

From [**1**′**a**]CF_3_SO_3_, freshly prepared from [**1a**]CF_3_SO_3_ (111 mg, 0.21 mmol) and methyl propargyl ether (0.17 mL, 2.0 mmol). Black solid, yield 114 mg (95%). Anal. calcd for C_20_H_22_F_3_Fe_2_NO_6_S: C, 41.91; H, 3.87; N, 2.44. Found: C, 42.06; H, 3.74; N, 2.51. IR (CH_2_Cl_2_): υ̃/cm^–1^ = 1990vs (CO), 1808s (μ-CO), 1681m (C_α_N). ^1^H NMR (acetone-*d*_6_): δ/ppm = 5.99, 5.79 (d, 2 H, ^2^*J*_HH_ = 11.7 Hz, CH_2_); 5.52, 5.19 (s, 10 H, Cp); 5.09 (s, 1 H, C_β_H); 3.94, 3.35 (s, 6 H, NMe_2_); 3.74 (s, 3 H, OMe). ^13^C{^1^H} NMR (acetone-*d*_6_): δ/ppm = 256.1 (μ-CO); 225.6 (C_α_); 210.6 (CO); 202.8 (C_γ_); 89.8, 87.6 (Cp); 85.3 (CH_2_); 58.2 (OMe); 50.9, 44.6 (NMe_2_); 47.9 (C_β_) (Figure 13).

#### [Fe_2_Cp_2_(CO)(μ-CO){μ-η^1^:η^3^-C_γ_(CH_2_OMe)C_β_HC_α_N(Me)(Xyl)}]CF_3_SO_3_, [**6b**]CF_3_SO_3_ (Figure 14)

**Figure 14:**
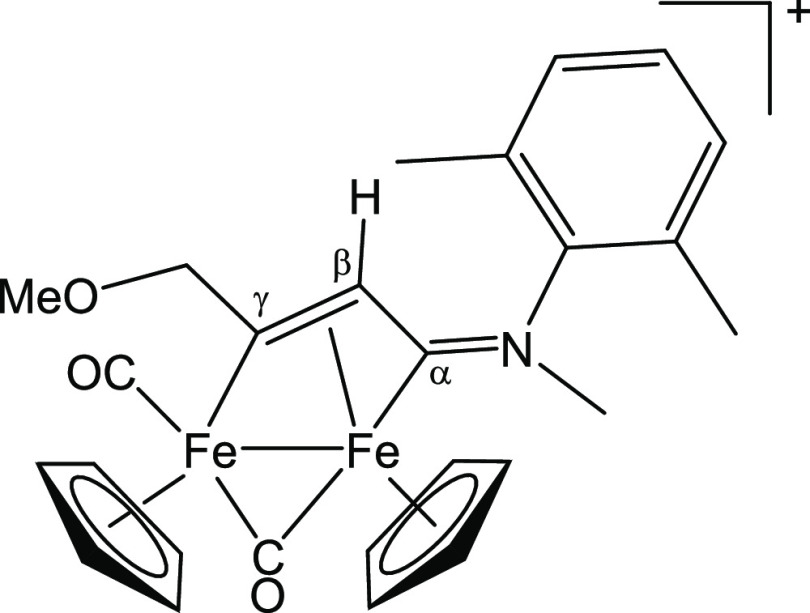
Structure of [**6b**]^+^.

From [**1**′**b**]CF_3_SO_3_, freshly prepared from [**1b**]CF_3_SO_3_ (79 mg, 0.13 mmol) and methyl propargyl ether (0.050 mL, 0.59 mmol). Dark-brown solid, yield 75 mg (88%). Anal. calcd for C_27_H_28_F_3_Fe_2_NO_6_S: C, 48.89; H, 4.26; N, 2.11. Found: C, 48.99; H, 4.17; N, 2.17. IR (CH_2_Cl_2_): υ̃/cm^–1^ = 2001vs (CO), 1814s (μ-CO), 1634m (C_α_N), 1587w (C–C_arom_). ^1^H NMR (CDCl_3_): δ/ppm = 7.17–7.09, 6.97–6.91 (m, 3 H, C_6_H_3_); 6.08, 5.53 (d, 2 H, ^2^*J*_HH_ = 14 Hz, CH_2_); 5.51, 5.27, 4.83 (s, 10 H, Cp); 4.68 (s, 1 H, C_β_H); 4.20, 3.49 (s, 3 H, NMe); 3.67 (s, 3 H, OMe); 2.48, 2.28, 1.97, 1.75 (s, 6 H, C_6_H_3_*Me*_2_). *E*/*Z* ratio = 11:1. ^13^C{^1^H} NMR (CDCl_3_): δ/ppm = 254.7 (μ-CO); 233.4 (C_α_); 209.8 (CO); 206.9 (C_γ_); 144.9 (*ipso*-C_6_H_3_); 131.4, 131.2, 129.6, 129.4, 129.3 (C_6_H_3_); 90.4, 87.9 (Cp); 85.5 (CH_2_); 59.0 (OMe); 47.3 (C_β_); 46.3 (NMe); 18.2, 17.2 (C_6_H_3_*Me*_2_) (Figure 14).

### Behavior in Aqueous Media

#### Solubility in D_2_O

The selected diiron compound was added to a D_2_O solution (0.7 mL) of Me_2_SO_2_ (*c* = 7.1 × 10^–3^ mol·L^–1^), and the resulting mixture was stirred at 21 °C for 30 min. The saturated solution was filtered to remove some solids, and then transferred into an NMR tube and analyzed by ^1^H NMR spectroscopy. The concentration (i.e., solubility) was calculated by the relative integral with respect to Me_2_SO_2_ as internal standard [δ/ppm = 3.14 (s, 6 H) in D_2_O)]. Solubility data are as follows. [**2**]CF_3_SO_3_: 6.5 × 10^–3^ M (6.1 g·L^–1^); [**3a**]CF_3_SO_3_: 2.2 × 10^–3^ M (2.0 g·L^–1^);

#### Stability in Aqueous Solution

The selected diiron compound (ca. 4 mg) was added to 1 mL of D_2_O/DMSO-*d*_6_ containing Me_2_SO_2_ (3.36 × 10^–3^ M), and the resulting mixture was stirred at ambient temperature for 30 min. The final mixture was filtered over celite, and the filtered solution was transferred into an NMR tube. The solution was analyzed by ^1^H NMR (“time0”) and subsequently heated at 37 °C for 72 h. After cooling to room temperature, the final solution was separated from a brown solid by filtration through celite, and the ^1^H NMR spectrum was recorded (delay time = 3 s; number of scans = 20). In each case, no new {FeCp} species was identified. The amount of starting material in solution (% with respect to the initial spectrum) was calculated by the relative integral with respect to Me_2_SO_2_ as the internal standard (δ/ppm = 3.14 (s, 6 H)), Table 1.^37^ NMR spectra at time0 were as follows.

[**2**]CF_3_SO_3_: ^1^H NMR (D_2_O): δ/ppm = 6.30–5.70 (m, 2 H, C_γ_CH_2_); 5.31, 4.97 (s, 10 H, Cp); 3.73, 3.16 (s, 6 H, NMe_2_).

[**3a**]CF_3_SO_3_: ^1^H NMR (D_2_O): δ/ppm = 6.10–5.80 (m, 2 H, C_γ_CH_2_); 5.32, 5.00 (s, 10 H, Cp); 3.74, *3.18*, 3.17 (s, 6 H, NMe_2_); 2.18, *2.16*, 2.10, 2.06, 2.03, 1.99, 1.98 (s, 12 H, 4× O=CMe).

[**3b**]CF_3_SO_3_: ^1^H NMR (DMSO-*d*_6_/D_2_O = 1:2): δ/ppm = 7.30–7.00 (m, 3 H, C_6_H_3_); 6.10–5.80 (m, 2 H, C_γ_CH_2_); 5.58, 5.54, 5.28, 5.27 (s, 10 H, Cp); 4.20, 4.19 (s, 3 H, NMe); 2.26, 2.25, 1.79, 1.78 (s, 6 H, C_6_H_3_*Me*_2_); 2.20, 2.19, 2.13, 2.10, 2.06, 2.04, 2.03, 2.00 (s, 12 H, 4× O=CMe).

[**4**]CF_3_SO_3_: ^1^H NMR (DMSO-*d*_6_/D_2_O = 1:2): δ/ppm = 6.10–5.75 (m, 2 H, C_γ_CH_2_); 5.41, 5.38, 5.09 (s, 10 H, Cp); 3.80, 3.24, 3.22 (s, 6 H, NMe_2_); 2.15–2.00 (s, 12 H, 4× O=CMe).

[**5a**]CF_3_SO_3_: ^1^H NMR (DMSO-*d*_6_/D_2_O = 1:2): δ/ppm = 6.00–5.70 (m, 2 H, C_γ_CH_2_); 5.35, 5.35, 5.02 (s, 10 H, Cp); 3.74, 3.16 (s, 6 H, NMe_2_); 1.55, 1.54, 1.46, 1.45, 1.44, 1.43, 1.35, 1.33 (s, 12 H, 2× CMe_2_).

[**5b**]CF_3_SO_3_: ^1^H NMR (DMSO-*d*_6_/D_2_O = 1:2): δ/ppm = 7.30–6.90 (m, 3 H, C_6_H_3_); 6.00–5.60 (m, 2 H, C_γ_CH_2_); 5.49, 5.48, 5.20 (s, 10 H, Cp); 4.13 (s, 3 H, NMe); 2.20, 1.72 (s, 6 H, C_6_H_3_*Me*_2_); 1.49, 1.42, 1.35, 1.34, 1.30, 1.22, 1.18 (s, 12 H, 2× CMe_2_).

[**6a**]CF_3_SO_3_: ^1^H NMR (DMSO-*d*_6_/D_2_O = 1:2): δ/ppm = 5.68 (m, 2 H, C_γ_CH_2_); 5.34, 5.00 (s, 10 H, Cp); 4.82 (s, 1 H, C_β_H); 3.73, 3.16 (s, 6 H, NMe_2_); 3.67 (s, 3 H, OMe).

[**6b**]CF_3_SO_3_: ^1^H NMR (DMSO-*d*_6_/D_2_O = 1:2): δ/ppm = 7.30–6.90 (m, 3 H, C_6_H_3_); 5.80–5.50 (m, 2 H, C_γ_CH_2_); 5.47, 5.19 (s, 10 H, Cp); 4.48 (s, 1 H, C_β_H); 4.13 (s, 3 H, NMe); 3.54 (s, 3 H, OMe); 2.19, 1.73 (s, 6 H, C_6_H_3_*Me*_2_).

#### Stability in Cell Culture Medium

The selected diiron compound (ca. 3 mg) was dissolved in DMSO (0.2 mL) in a glass tube, and then 4 mL of RPMI-1640 medium (Merck; modified with sodium bicarbonate, without l-glutamine and phenol red, liquid, sterile-filtered, suitable for cell culture) was added. A portion of the resulting solution was diluted 1:1000 with acetonitrile, filtered on a poly(tetrafluoroethylene) (PTFE) filter (0.45 μm pore size), and analyzed by flow injection ESI-MS (time0), while the remaining solution was kept at 37 °C for 72 h and stored in the dark. Then, the final mixture was diluted 1:1000 with acetonitrile, filtered on a PTFE filter (0.45 μm pore size), and analyzed by flow injection ESI-MS (injection volume = 0.1–1 μL, depending on the instrumental response; eluent = acetonitrile). The amount of unaltered complex in solution (% with respect to the time0 mass spectrum) was calculated as the ratio between the intensity of the corresponding molecular ions, Table 1. Assuming a comparable ionizability for diiron vinyliminium complexes (with or without the sugar moiety), the overall percentage of all diiron species in solution, compared to the starting complex at time0, is also provided. Mass spectra after 72 h are displayed in Figures S43–S50 and are as follows.

[**2**]CF_3_SO_3_: [**6a**]^+^ (*m*/*z* calcd for [C_19_H_22_Fe_2_NO_3_]^+^ 424.0299, found 424.0296, error: −0.7 ppm) + [**9a**]^+^ (*m*/*z* calcd for [C_18_H_20_Fe_2_NO_3_]^+^ 410.0142, found 410.0137, error: −1.2 ppm), ratio [**6a**]^+^:[**9a**]^+^ = 55:1.

[**3a**]CF_3_SO_3_: [**3a**]^+^ (*m*/*z* calcd for [C_32_H_38_Fe_2_NO_12_]^+^ 740.1094, found 740.1087, error: −0.9 ppm) + [**3a-Ac+H**]^+^ (*m*/*z* calcd for [C_30_H_36_Fe_2_NO_11_]^+^ 698.0988, found 698.0975, error: −1.8 ppm) + [**3a-2Ac+2H**]^+^ (*m*/*z* calcd for [C_28_H_34_Fe_2_NO_10_]^+^ 656.0883, found 656.0871, error: −1.8 ppm), ratio [**3a**]^+^:[**3a-Ac+H**]^+^:[**3a-2Ac+2H**]^+^ = 56:11:1.

[**3b**]CF_3_SO_3_: [**3b**]^+^ (*m*/*z* calcd for [C_39_H_44_Fe_2_NO_12_]^+^ 830.1564, found 830.1570, error: 0.7 ppm) + [**3b-Ac+H**]^+^ (*m*/*z* calcd for [C_37_H_42_Fe_2_NO_11_]^+^ 788.1458, found 788.1459, error: 0.1 ppm) + [**3b-2Ac+2H**]^+^ (*m*/*z* calcd for [C_35_H_40_Fe_2_NO_10_]^+^ 746.1352, found 746.1353, error: 0.1 ppm), ratio [**3b**]^+^:[**3b-Ac+H**]^+^:[**3b-2Ac+2H**]^+^ = 11:3:1.

[**4**]CF_3_SO_3_: [**4**]^+^ (*m*/*z* calcd for [C_32_H_38_Fe_2_NO_12_]^+^ 740.1094, found 740.1099, error: 0.7 ppm) + [**4-Ac+H**]^+^ (*m*/*z* calcd for [C_30_H_36_Fe_2_NO_11_]^+^ 698.0988, found 698.0991, error: 0.4 ppm) + [**4-2Ac+2H**]^+^ (*m*/*z* calcd for [C_28_H_34_Fe_2_NO_10_]^+^ 656.0883, found 656.0895, error: 1.8 ppm), ratio [**4**]^+^:[**4-Ac+H**]^+^:[**4-2Ac+2H**]^+^ = 4:2:1.

[**5a**]CF_3_SO_3_: [**5a**]^+^ (*m*/*z* calcd for [C_30_H_38_Fe_2_NO_8_]^+^ 652.1297, found 652.1300, error: 0.5 ppm).

[**5b**]CF_3_SO_3_: [**5b**]^+^ (*m*/*z* calcd for [C_37_H_44_Fe_2_NO_8_]^+^ 742.1767, found 742.1778, error: 1.5 ppm).

[**6a**]CF_3_SO_3_: [**6a**]^+^ (*m*/*z* calcd for [C_19_H_22_Fe_2_NO_3_]^+^ 424.0299, found 424.0293, error: −1.4 ppm) + [**9a**]^+^ (*m*/*z* calcd for [C_18_H_20_Fe_2_NO_3_]^+^ 410.0142, found 410.0128, error: −3.4 ppm), ratio [**6a**]^+^:[**9a**]^+^ = 55:1.

[**6b**]CF_3_SO_3_: [**6b**]^+^ (*m*/*z* calcd for [C_26_H_28_Fe_2_NO_3_]^+^ 514.0769, found 514.0775, error: 1.2 ppm).

All of the isotopic patterns fit well the corresponding calculated ones.

### Determination of Partition Coefficients (Log *P*_ow_)

Partition coefficients (*P*_ow_; IUPAC: *K*_D_ partition constant,^38^ defined as *P*_ow_ = *c*_org_/*c*_aq_, where *c*_org_ and *c*_aq_ are the molar concentrations of the selected compound in the organic and aqueous phases, respectively, were determined by the shake-flask method and UV–vis measurements.^37,39^ Values of Log *P*_ow_ for diiron complexes are compiled in Table 2. All of the operations were carried out at 21 ± 1 °C. Deionized water and 1-octanol were mixed and vigorously stirred for 24 h at ambient temperature to allow saturation of both phases, then separated by centrifugation, and used for the following experiments. A solution of the selected diiron compound in octanol-saturated water (*V* = 5 mL) was prepared and its UV–vis spectrum was recorded. An aliquot of the solution (*V*_aq_ = 1.5 mL) was then transferred into a test tube and the organic phase (*V*_org_ = *V*_aq_ = 1.5 mL) was added. The mixture was vigorously stirred for 20 min, and the resulting emulsion was centrifuged (5000 rpm, 10′). Hence, the UV–vis spectrum of the aqueous phase was recorded. The procedure was repeated three times for each compound. The partition coefficient was then calculated as , where *A*_0,aq_ and *A*_aq_ are the absorbance values in the aqueous phase, respectively, before and after partition with the organic phase.^39^ For [**6b**]CF_3_SO_3_, an inverse procedure was followed, starting from a solution of the compound in water-saturated octanol. The partition coefficient was calculated as *P*_ow_ = *A*_org_/(*A*_org_^0^ – *A*_org_) where *A*_org_^0^ and *A*_org_ are the absorbances in the organic phase, respectively, before and after partition with the aqueous phase. UV–vis measurements were carried out using 1 cm PMMA cuvettes. The wavelength of the maximum absorption of each compound (415–400 nm range) was used for UV–vis quantification.

### Cell Culture and Cytotoxicity Studies

#### Assessment of Cytotoxic Activity

CT26 (mouse colon carcinoma) and MCF-7 (human breast adenocarcinoma) cells were cultured in DMEM, U87 (human glioblastoma) cells were cultured in MEM, and RPE-1 (human normal retina pigmented epithelium) cells were cultured in DMEM/F-12 media (Gibco). All of the culture media were supplemented with 10% fetal calf serum (Gibco) and 1% PenStrep (Gibco). Cells were maintained in a humidified atmosphere at 37°C and 5% CO_2_.

Cells were seeded at a 4.000 cells/well density in flat-bottom 96-well plates (100 μL/well) and were incubated at 37°C for 24 h to allow the cells to attach to the bottom of the wells. Stock solutions of the diiron compounds were prepared in DMSO and rapidly diluted in a medium (1% DMSO content maximum). The stock solution of the reference drug cisplatin was prepared in saline solution, NaCl 0.9% w/v. The medium was replaced by dilutions of tested compounds in a fresh medium (100 μL/well) to obtain the following concentration range: 0.01, 0.03, 0.1, 0.3, 1, 3, 10, 30, and 100 μM for the tested compounds and 0.3, 0.6, 2, 3, 6, 10, and 30 μM for the reference drug cisplatin. After loading the drug, cells were incubated for 48 h at 37 °C. The medium was then replaced with 100 μL of a fresh medium containing resazurin (0.2 mg mL^–1)^ and incubated for 4 h. The florescence of the wells, directly proportional to the number of survived cells, was determined by reading the plates using a SpectraMaxM2 Microplate Reader (λ_exc_ = 540 nm; λ_read_ = 590 nm). Fluorescence data were normalized by attributing 100% cell viability to the mean signal obtained for the lowest compound concentration and 0% to the signal obtained from wells containing the highest drug concentration or only the resazurin solution (when no toxicity was observed). Data were fitted using GraphPad Prism Software (v6) and IC_50_ values were calculated by nonlinear regression. All experiments were performed in triplicates.

#### Viability Test With No-Glucose Medium

CT26 cells were seeded at a 4.000 cells/well density in flat-bottom 96-well plates (100 μL/well) and were incubated at 37 °C for 8 h to allow the attachment of cells to the bottom of the wells. After 8 h, the medium was carefully removed and replaced with no-glucose DMEM. The cells were incubated overnight. Stock solutions of the compounds were prepared in DMSO and rapidly diluted in a medium without glucose (1% DMSO content maximum). The medium was replaced by dilutions of tested compounds in a fresh no-glucose medium (100 μL/well) to obtain the following concentration ranges: 0.3, 1, 3, 10, 30, and 100 μM for the tested compounds and 0.03, 0.1, 0,3, 1, 3, and 30 μM for the reference drug cisplatin. After loading the drug, the cells were incubated for 48 h at 37 °C. The medium was then replaced with 100 μL/well of a fresh medium containing resazurin (0.2 mg mL^–1^) and incubated for 4 h. The fluorescence of the wells, directly proportional to the number of survived cells, was determined by reading the plates using a SpectraMaxM2 Microplate Reader (λ_exc_ = 540 nm; λ_read_ = 590 nm). Fluorescence data were normalized by attributing 100% cell viability to the mean signal obtained for the lowest compound concentration and 0% to the signal obtained from wells containing the highest drug concentration or only the resazurin solution (when no toxicity was observed). Data were fitted using GraphPad Prism Software (v6), and IC_50_ values were calculated by nonlinear regression. All experiments were performed in triplicates.

#### Scratch Assay

CT26 cells were seeded at 2 × 10^5^ cells/well density in a 6-well plate. The cells were incubated for 48 h to obtain a 90–100% confluency. The cellular monolayer was scratched with a 200 μL tip, the cells were washed once with PBS to remove the debris, and then 4 mL of the solution containing IC_20_ of each tested drug was added to the wells. Less than 1% of DMSO was used in the preparation of the drug solutions. The cells were monitored by imaging over 30 h with the following time intervals: 1, 3, 8, 24, 30 h. Agilent BioTek Gen 5 Cytation was used to record the pictures. The cells were maintained at 37 °C during the time needed for the imaging. The images are representative from one successive experiment out of three successive individual experiments.

## Supporting Information Available

Synthesis and ^1^H NMR characterization of carbohydrate alkynes (Figures S1–S4 and S5–S8); IR spectra (Figures S9–S17); NMR spectra (Figures S18–S42); ESI-MS spectra (Figures S43–S50); and dose–response cell viability curves (Figures S51–S55) (PDF)
