# Supplementary material for: Tethering Carbohydrates to the Vinyliminium Ligand of Antiproliferative Organometallic Diiron Complexes
Source: Organometallics. 2022 Feb 28;41(5):514–26. doi: 10.1021/acs.organomet.1c00519 (PMC8924928; doi:10.1021/acs.organomet.1c00519)

Tethering Carbohydrates to the Vinyliminium Ligand of Antiproliferative Organometallic Diiron Complexes

Silvia Schoch,[a,$] Dalila Iacopini,[a,$] Maria Dalla Pozza,[b,$] Sebastiano Di Pietro,[c] Ilaria Degano,[a] Gilles Gasser,[b,*] Valeria Di Bussolo,[c,*] Fabio Marchetti [a,*]

[a] University of Pisa, Department of Chemistry and Industrial Chemistry, 56124 Pisa, Italy.

[b] Chimie ParisTech, PSL University, CNRS, Institute of Chemistry for Life and Health, Paris, France.

[c] University of Pisa, Department of Pharmacy, 56126 Pisa, Italy.

* gilles.gasser@chimieparistech.psl.eu; valeria.dibussolo@unipi.it; fabio.marchetti1974@unipi.it

Supporting Information

Pages

Synthesis of carbohydrate-functionalized alkynes (**Figures S1-S4**) S2-S5

Figures S5-S8: ^{1}H NMR spectra of carbohydrate-functionalized alkynes S6-S7

Figures S9-S17: IR spectra of diiron complexes S8-S10

Figures S18-S34: ^{1}H and ^{13}C NMR spectra of diiron complexes S11-S19

Figures S35-S42: ^{1}H spectra of diiron complexes in aqueous solution S20-S23

Figures S43-S50: ESI-MS spectra of diiron complexes in cell culture medium S24-S26

Figures S51-S55: Dose-response cell viability curves S27-S31

Synthesis and characterization of sugar-functionalized alkynes

1-*O*-(2-Propynyl)-α-D-mannopyranose (Figure S1) [1]

Figure S1. Structure of **HC≡CCH$_2$OMan**

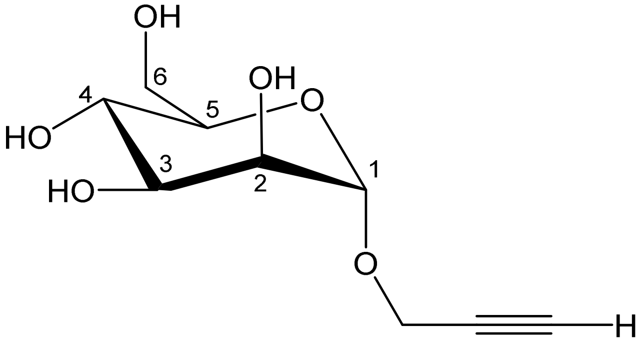

According to the literature procedure, D-mannose (500 mg, 2.77 mmol) was suspended in propargyl alcohol (1.0 mL, 13.8 mmol) and the resulting mixture was stirred at 65 °C. H_2SO_4-treated silica (14 mg) was added, and the stirring was continued for 3 h, until complete dissolution of the solids. The conversion of the starting D-mannose was monitored by TLC (CH_2Cl_2/MeOH; 5:1). After cooling to room temperature, the reaction mixture was transferred to a short (4 cm x 2 cm) silica gel column and the excess propargyl alcohol was eluted with CH_2Cl_2 (100 mL), followed by elution of the propargyl glycoside with CH_2Cl_2/EtOH (5:1). The title product was obtained as a colorless oil upon removal of the volatiles under vacuum. Yield 500 mg, 83%. ^{1}H NMR (CD_3OD): δ/ppm = 4.96 (d, 1 H, J = 1.4 Hz, H^1); 4.28-4.27 (d, 2 H, ^4J = 2.3 Hz, CH_2C≡); 3.85-3.78 (m, 2 H, H^3 + H^2); 3.73-3.64 (m, 2 H, H^4 + H^{6a}); 3.62-3.51 (m, 2 H, H^5 + H^{6b}); 2.88-2.86 (br-t, 1 H, ≡CH).

[1] B. Roy, B. Mukhopadhyay, *Tetrahedron Letters* **2007**, *48*, 3783-3787.

2,3,4,6-Tetra-*O*-acetyl-1-*O*-(2-propinyl)-α-D-mannopyranose (Figure S2) [1]

Figure S2. Structure of **HC≡CCH$_2$OMan'**

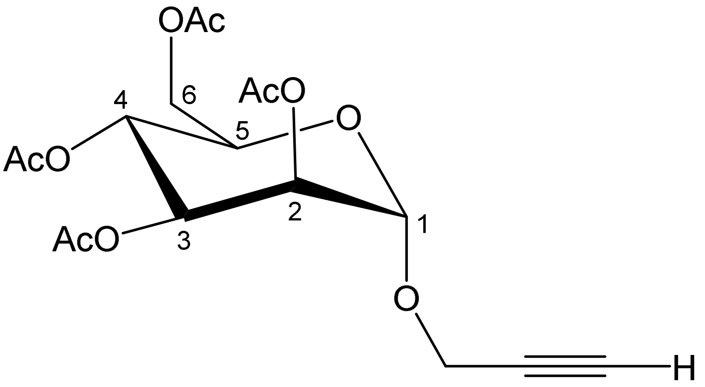

1-*O*-(2-Propynyl)-α-D-mannopyranose (250 mg, 1.14 mmol) was dissolved in pyridine (4 mL, 50 mmol) and the mixture was stirred at room temperature for 10 min. Then, acetic anhydride (2.0 mL, 21 mmol) was added dropwise and the reaction was completed after 3 hours. The mixture was evaporated under reduced pressure and the crude was purified by flash column chromatography (SiO_2, hexane/ethyl acetate 6:4 v/v as eluent). The title product was obtained as a colorless oil upon removal of the volatiles under vacuum. Yield 330 mg, 75%. ^{1}H NMR ($CDCl_3$): δ/ppm = 5.21-5.16 (m, 3 H, H^3 + H^2 + H^4); 4.93 (br-s, 1 H, H^1); 4.22-4.15 (m, 3 H, CH_2C≡ + H^{6a}); 4.02-3.92 (m, 2 H, H^{6b} + H^5); 2.44 (br-t, 1 H, ≡CH); 2.06, 2.00, 1.94, 1.88 (s, 12 H, 4 x O=CMe).

2,3,4,6-tetra-*O*-Acetyl-1-*O*-(2-propynyl)-α-D-glucopyranose (Figure S3) [1]

Figure S3. Structure of **HC≡CCH2OGlu'**

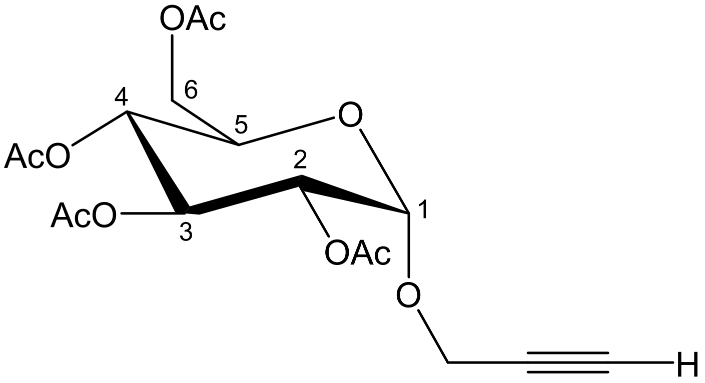

α-Propargyl-D-glucopyranoside (500 mg, 2.28 mmol), synthesized according to the literature,[1] was dissolved in pyridine (8.0 mL, 50 mmol), and the mixture was stirred at room temperature for 10 min. Later, acetic anhydride (4.0 mL, 21 mmol) was added dropwise, and the per-O-acetylation reaction was completed after 3 hours. The mixture was evaporated under reduced pressure and the crude, containing both α- and β-anomers, was passed through automated flash column chromatography (SiO_2 100 g, gradient of hexane/ethyl acetate mixture as eluent) to afford the pure α-glycoside. The title product was then obtained as a colorless oil upon removal of the volatiles under vacuum. Yield 266 mg, 30%. ^{1}H NMR ($CDCl_3$): δ/ppm = 5.50-5.45 (t, 1 H, ^3J = 9.8 Hz, H^3); 5.27-5.26 (d, 1 H, ^3J = 3.8 Hz, H^1); 5.10-5.05 (t, 1 H, ^3J = 9.8 Hz, H^4); 4.92-4.88 (dd, 1 H, ^3J = 3.9 Hz, 10.3 Hz, H^2); 4.27-4.23 (m, 3 H, $CH_2C≡$ + H^{6a}); 4.10-4.04 (m, 2 H, H^5 + H^{6b}); 2.44-2.43 (t, 1 H, ^4J = 2.4 Hz, ≡CH); 2.08, 2.06, 2.01, 2.00 (s, 12 H, 4 x O=CMe).

2,3:4,5-di-*O*-isopropylidene-1-*O*-(2-propynyl)-β-D-fructopyranose (Figure S4) [2]

Figure S4. Structure of **HC≡CCH₂OFru'**

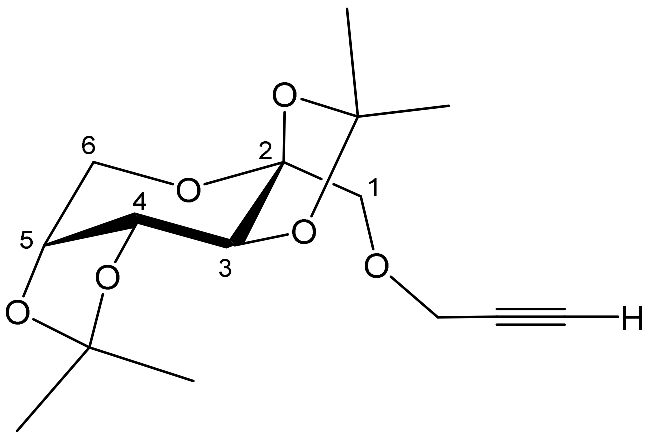

Commercial 2,3,4,5-di-*O*-isopropylidene-β-D-fructopyranose (300 mg, 1.15 mmol) was dissolved in THF (3 mL) and the mixture was stirred at 0 °C for 10 min. After complete solubilization, tetrabutylammonium iodide (43 mg, 0.12 mmol) was added, followed by an aqueous solution of NaOH 60% w/v (1.15 mL) and propargyl bromide (0.25 mL, 2.87 mmol). The resulting solution was warmed to room temperature and stirred for 48 h. The obtained mixture was diluted with Et_2O and washed with H_2O (3 x 5 mL). The organic phase was dried over anhydrous Na_2SO_4, filtered and evaporated to dryness. The crude was purified by flash column chromatography (SiO_2, 1:1 hexane/ethyl acetate mixture as eluent). The product was obtained as a yellow oil upon removal of the volatiles under vacuum. Yield 200 mg, 60%. ^{1}H NMR ($CDCl_3$): δ/ppm = 4.60-4.57 (dd, 1 H, ^3J = 2.4 Hz, 7.8 Hz, H^4); 4.36-4.35 (d, 1 H, ^3J = 2.6 Hz, H^3); 4.30-4.26 (m, 1 H, H^5); 4.26-4.23 (m, 2 H, H^{6a} + H^{6b}); 3.91-3.88 (dd, 1 H, ^4J = 1.8 Hz, ^2J = 12.9 Hz, CH_2C≡); 3.75-3.71 (d, 1 H, ^2J = 12.9 Hz, CH_2C≡); 3.66 (s, 2 H, H^{1a} + H^{1b}); 2.41-2.40 (t, 1 H, ^4J = 2.5 Hz, ≡CH); 1.53, 1.46, 1.41, 1.33 (s, 12 H, 4 x O=CMe).

[2] A. Hausherr, B. Orschel, S. Scheler, H.-U. Reissig, *Synthesis* **2001**, 9, 1377-1385.

Figure S5. ^{1}H NMR spectrum (401 MHz, CD_3OD) of **HC≡CCH₂OMan**.

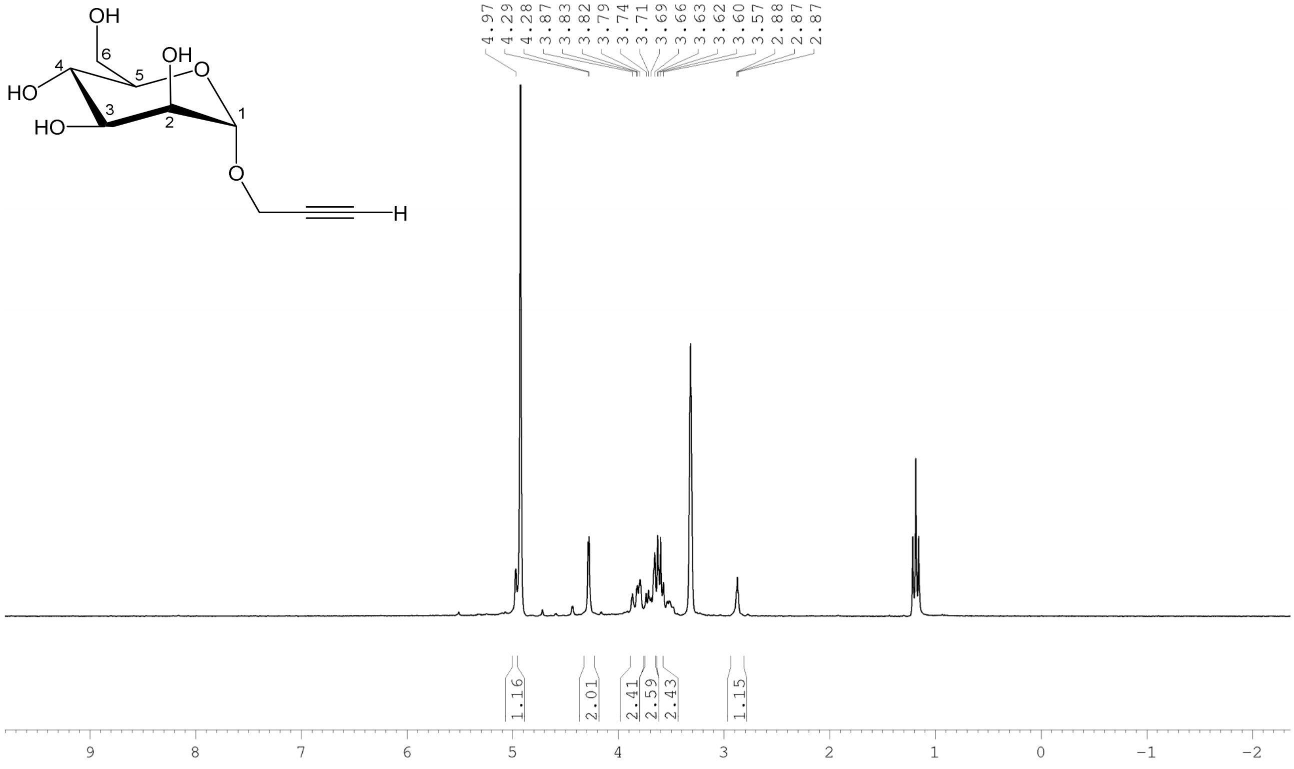

Figure S6. ^{1}H NMR spectrum (401 MHz, CD_3OD) of **HC≡CCH₂OMan'**.

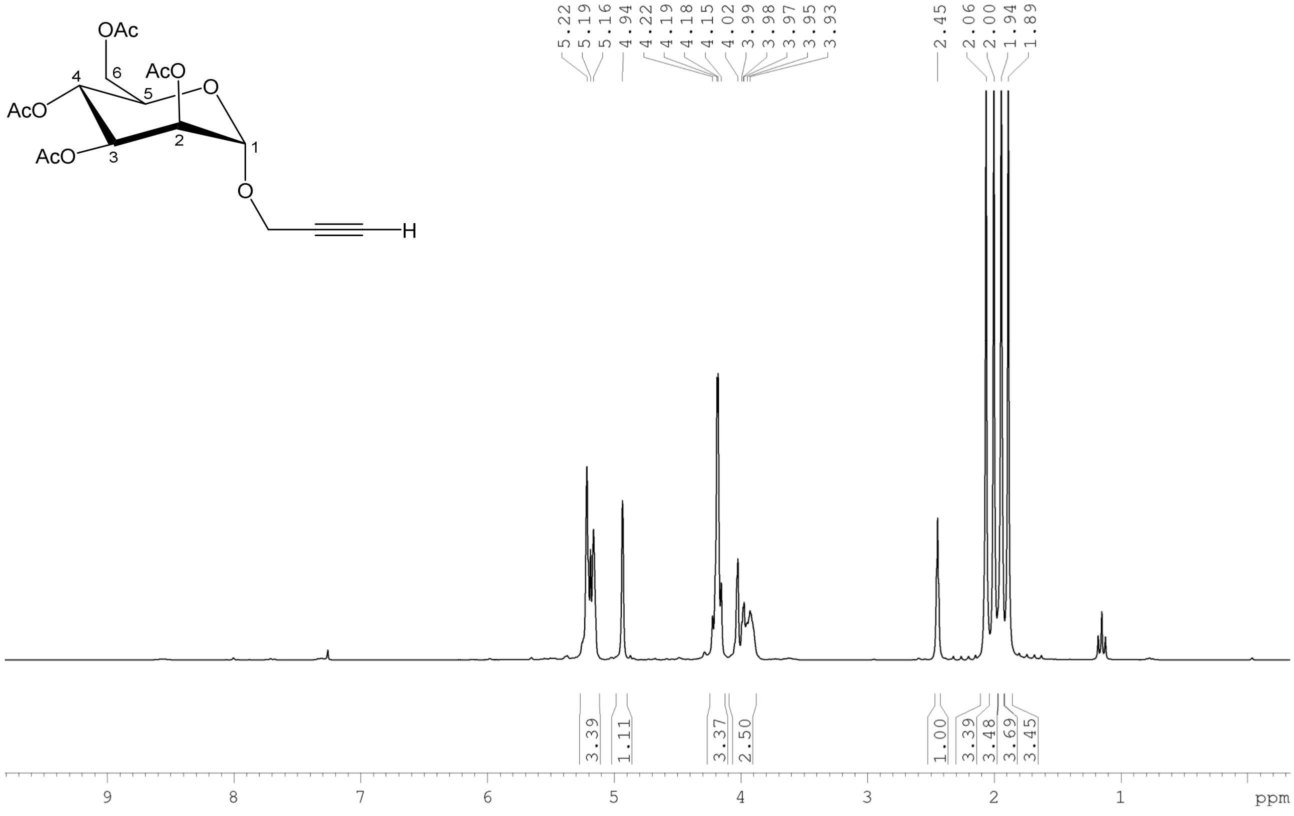

Figure S7. ^{1}H NMR spectrum (401 MHz, CD_3OD) of **HC≡CCH₂OGlu'**.

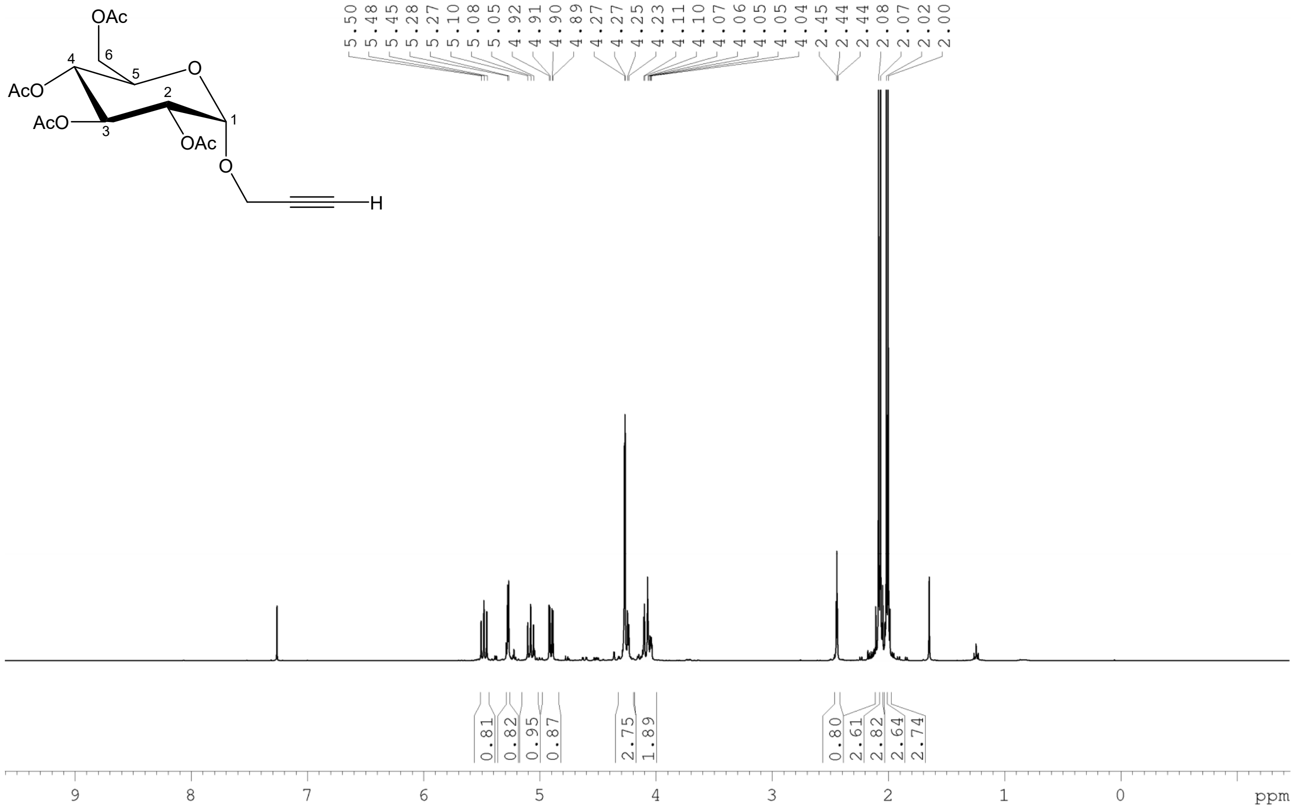

Figure S8. ^{1}H NMR spectrum (401 MHz, CD_3OD) of **HC≡CCH₂OFru'**.

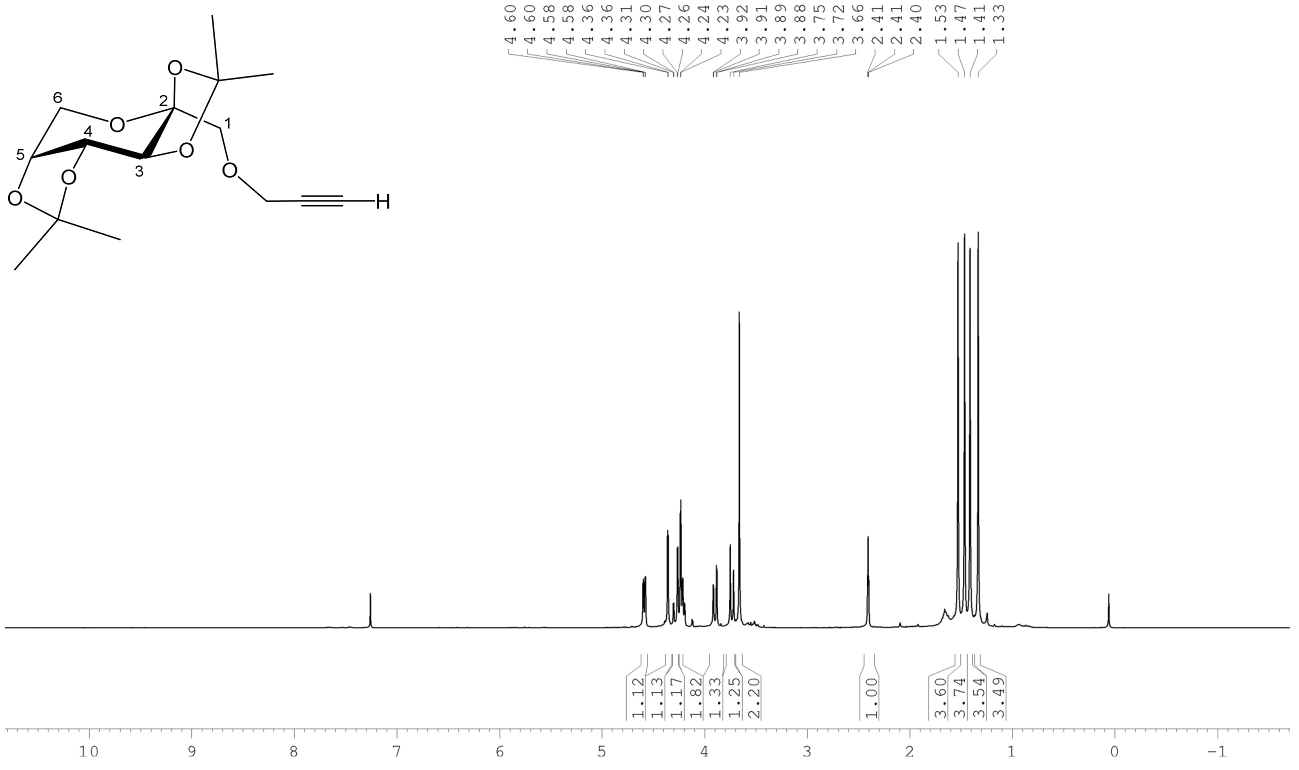

Figure S9. IR spectrum of **1a-Cl** in CH_2Cl_2 solution.

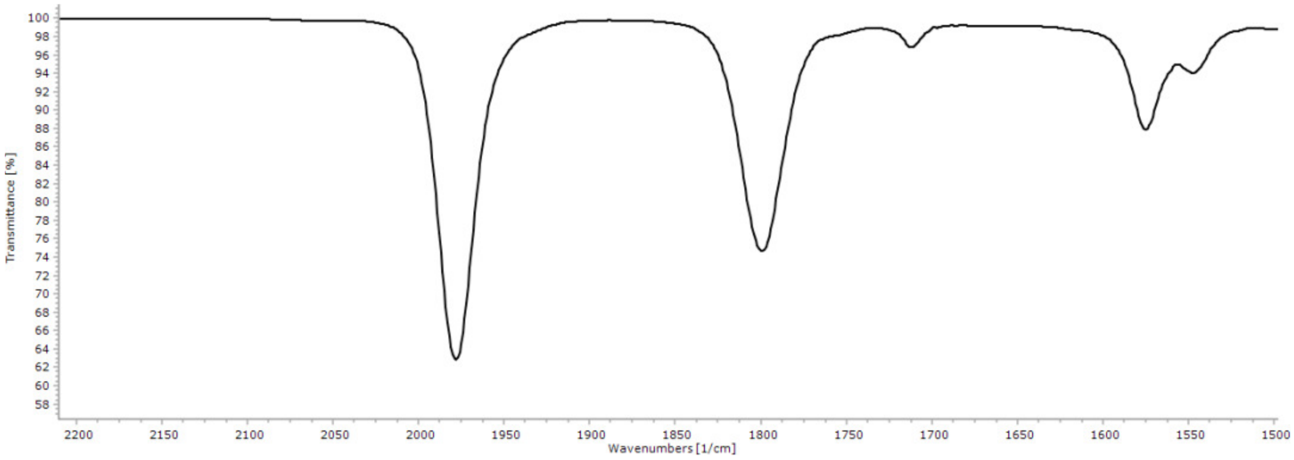

Figure S10. IR spectrum of [**2**]CF_3SO_3 in CH_3OH solution.

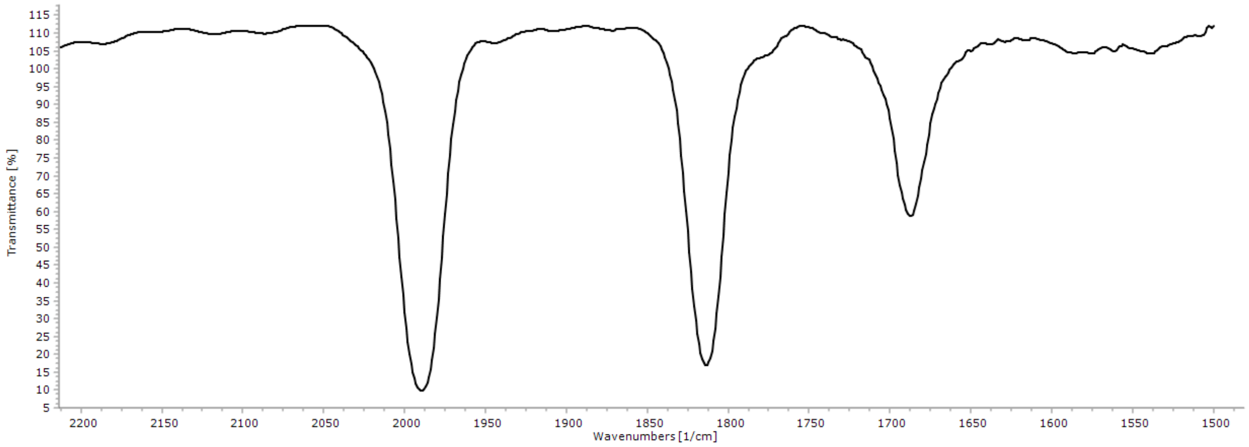

Figure S11. IR spectrum of [**3a**]CF_3SO_3 in CH_2Cl_2 solution.

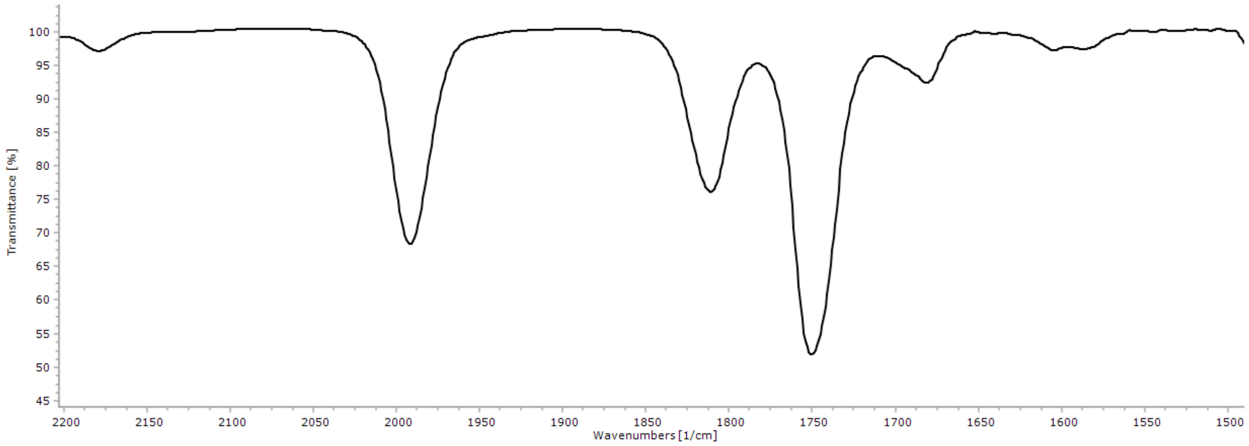

Figure S12. IR spectrum of [**3b**]CF_3SO_3 in CH_2Cl_2 solution.

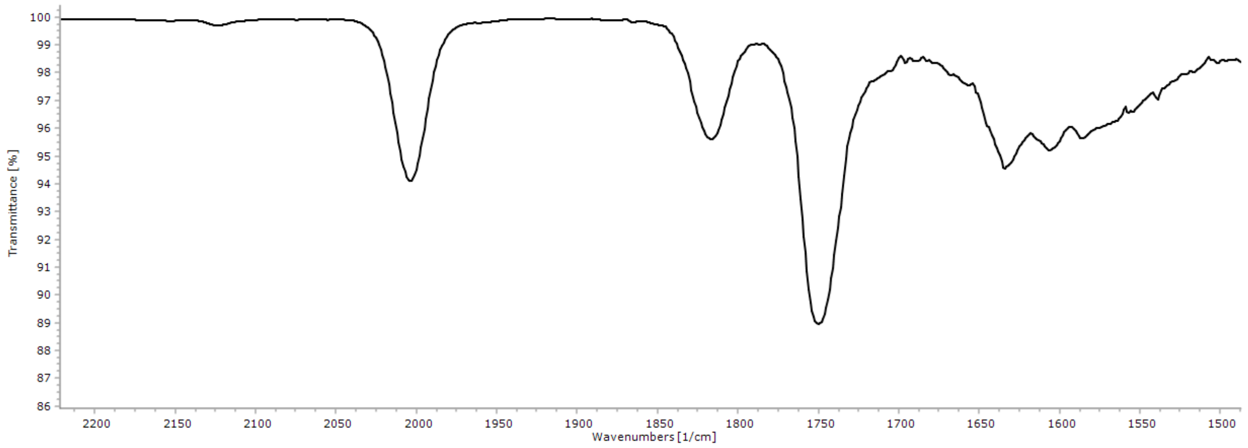

Figure S13. IR spectrum of [**4**]CF_3SO_3 in CH_2Cl_2 solution.

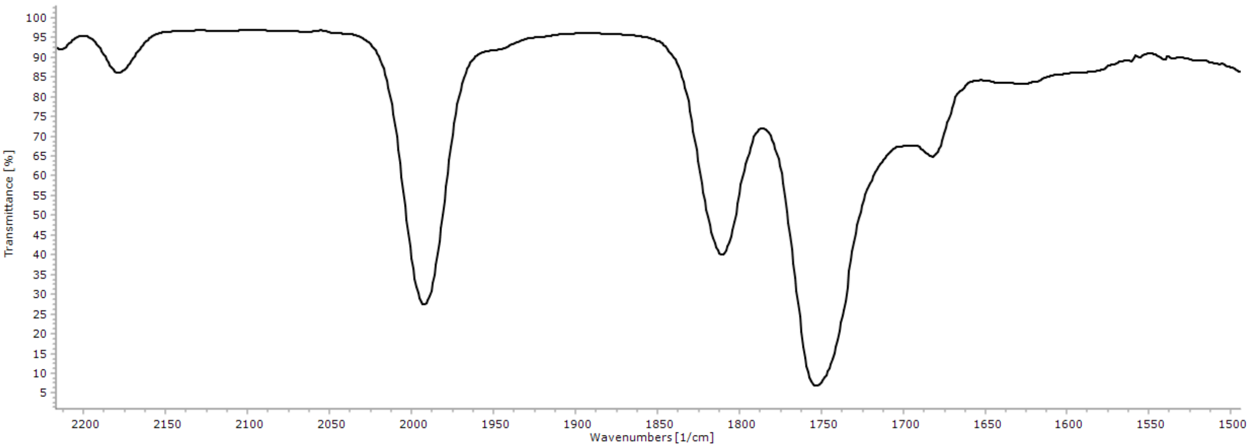

Figure S14. IR spectrum of [**5a**]CF_3SO_3 in CH_2Cl_2 solution.

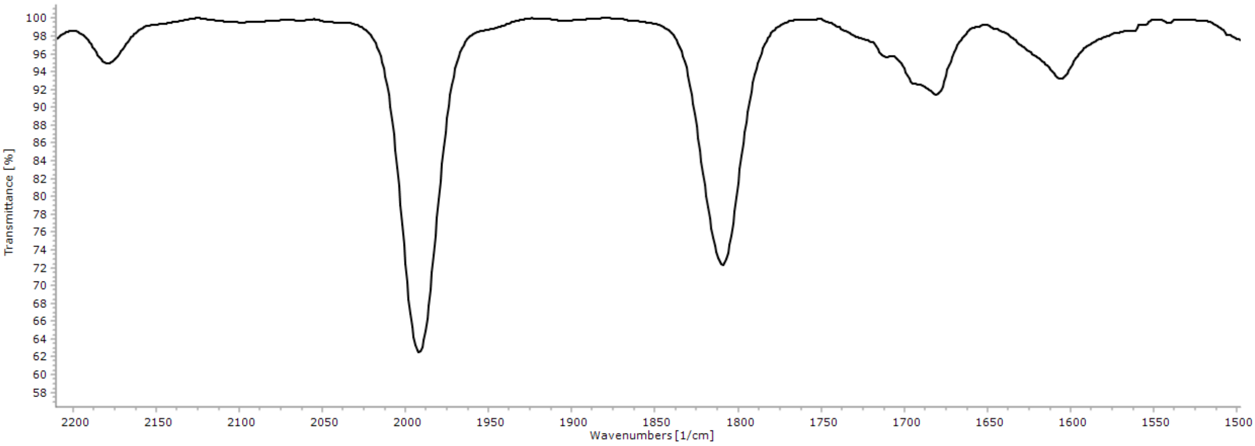

Figure S15. IR spectrum of [**5b**]CF_3SO_3 in CH_2Cl_2 solution.

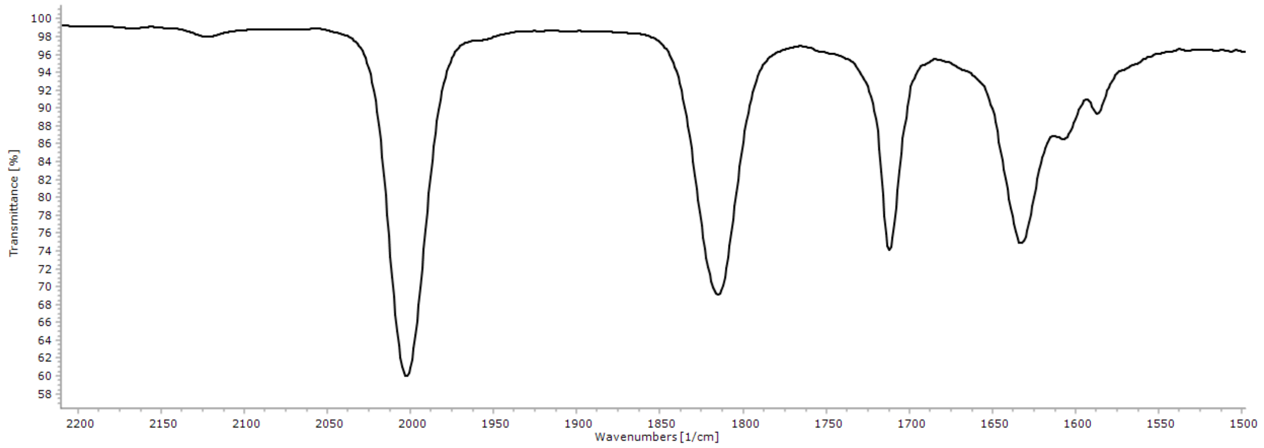

Figure S16. IR spectrum of [**6a**]CF_3SO_3 in CH_2Cl_2 solution.

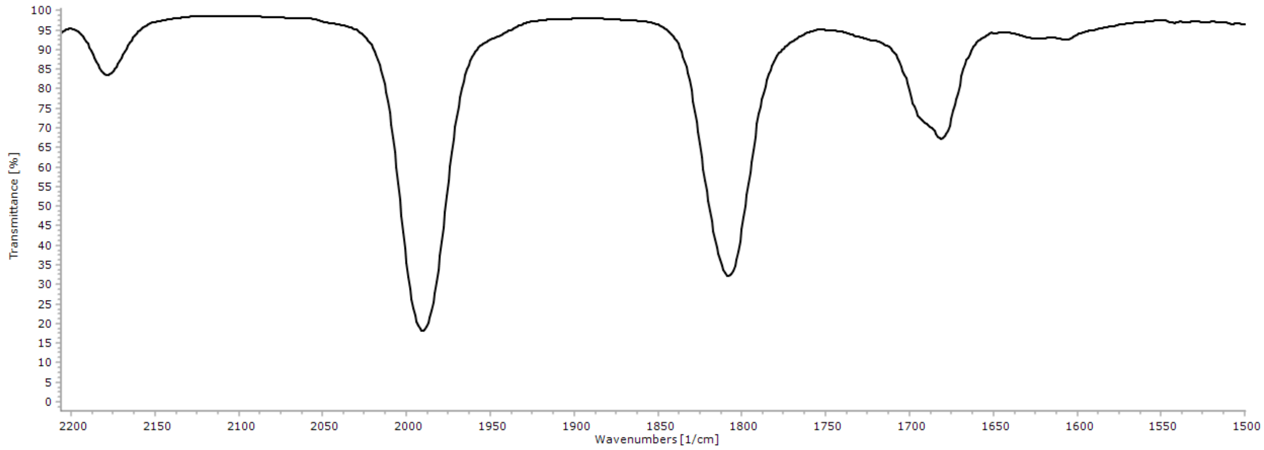

Figure S17. IR spectrum of [**6b**]CF_3SO_3 in CH_2Cl_2 solution.

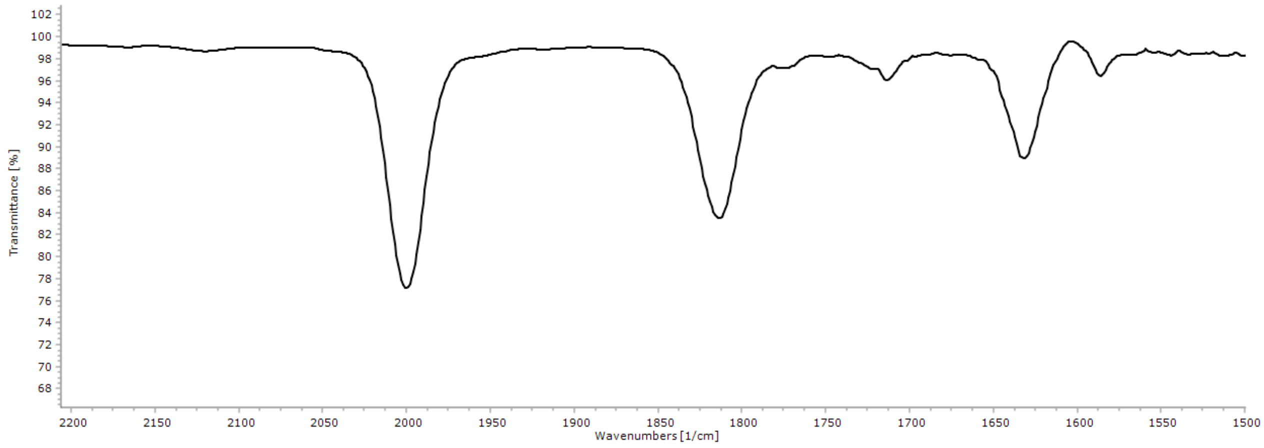

Figure S18. ^{1}H NMR spectrum (401 MHz, $CDCl_3$) of **1a-Cl**.

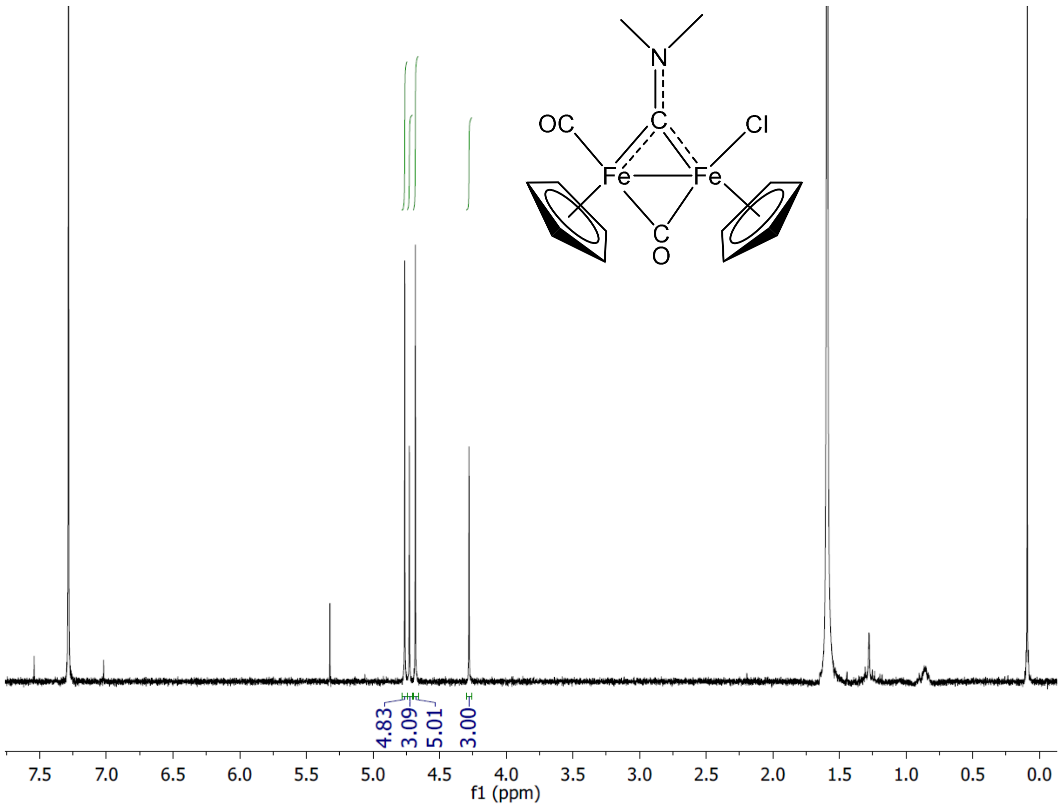

Figure S19. ^{1}H NMR spectrum (401 MHz, acetone-d$_6$) of [**2**]CF_3SO_3.

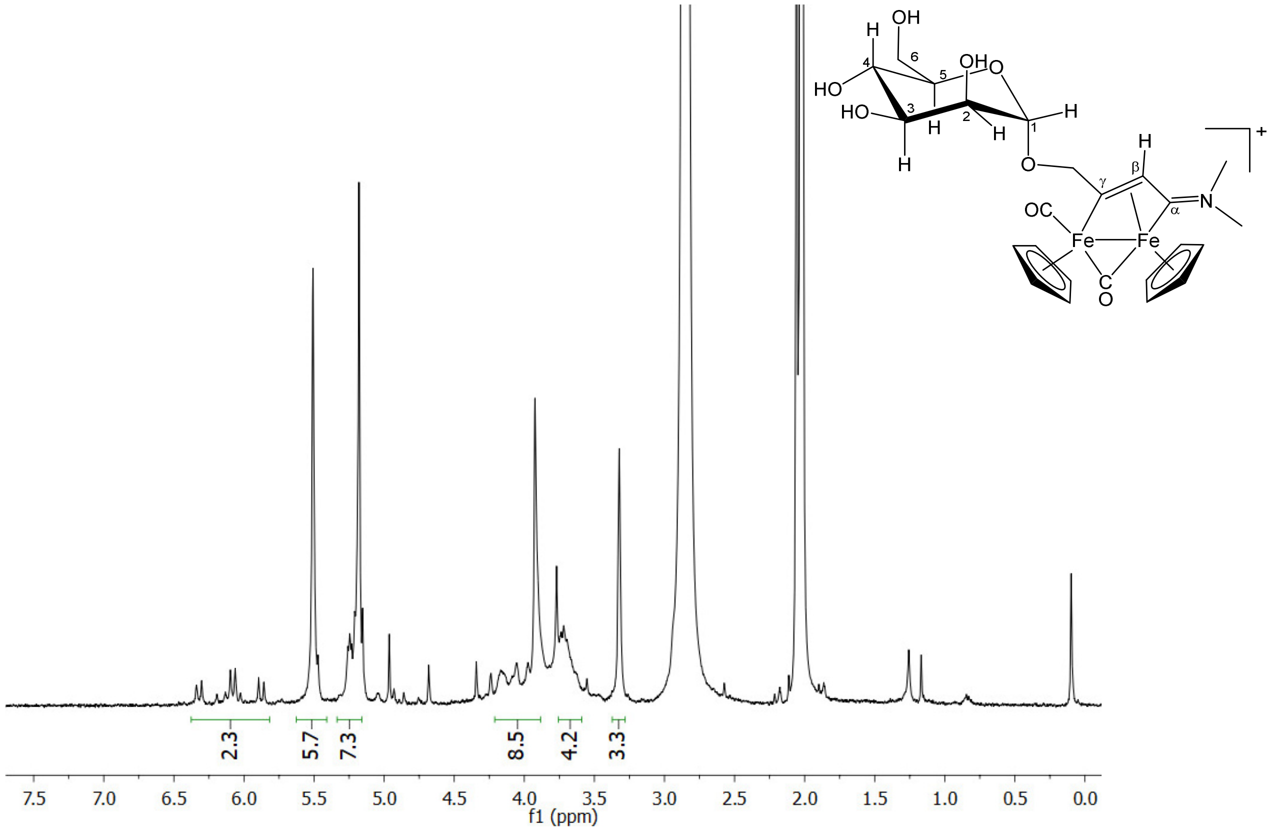

Figure S20. ^{13}C{^{1}H} NMR spectrum (101 MHz, acetone-d$_6$) of [**2**]CF_3SO_3.

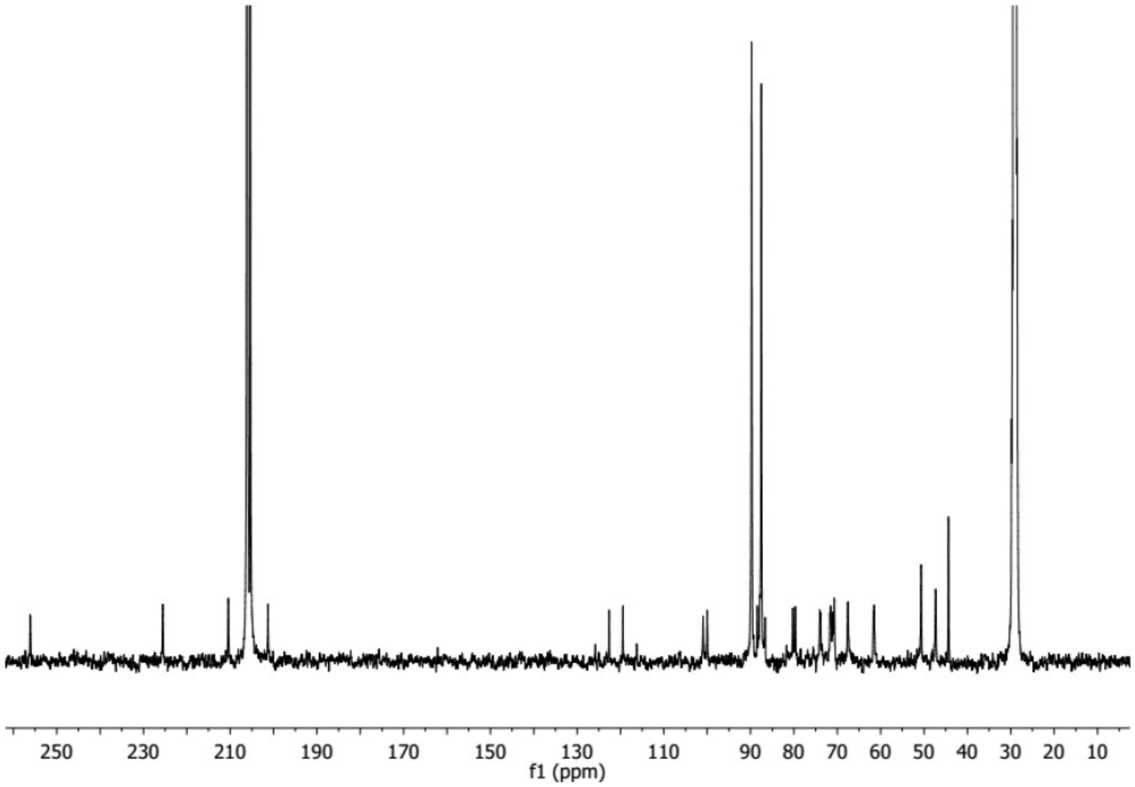

Figure S21. ^{1}H NMR spectrum (401 MHz, acetone-d$_6$) of [**3a**]CF$_3$SO$_3$.

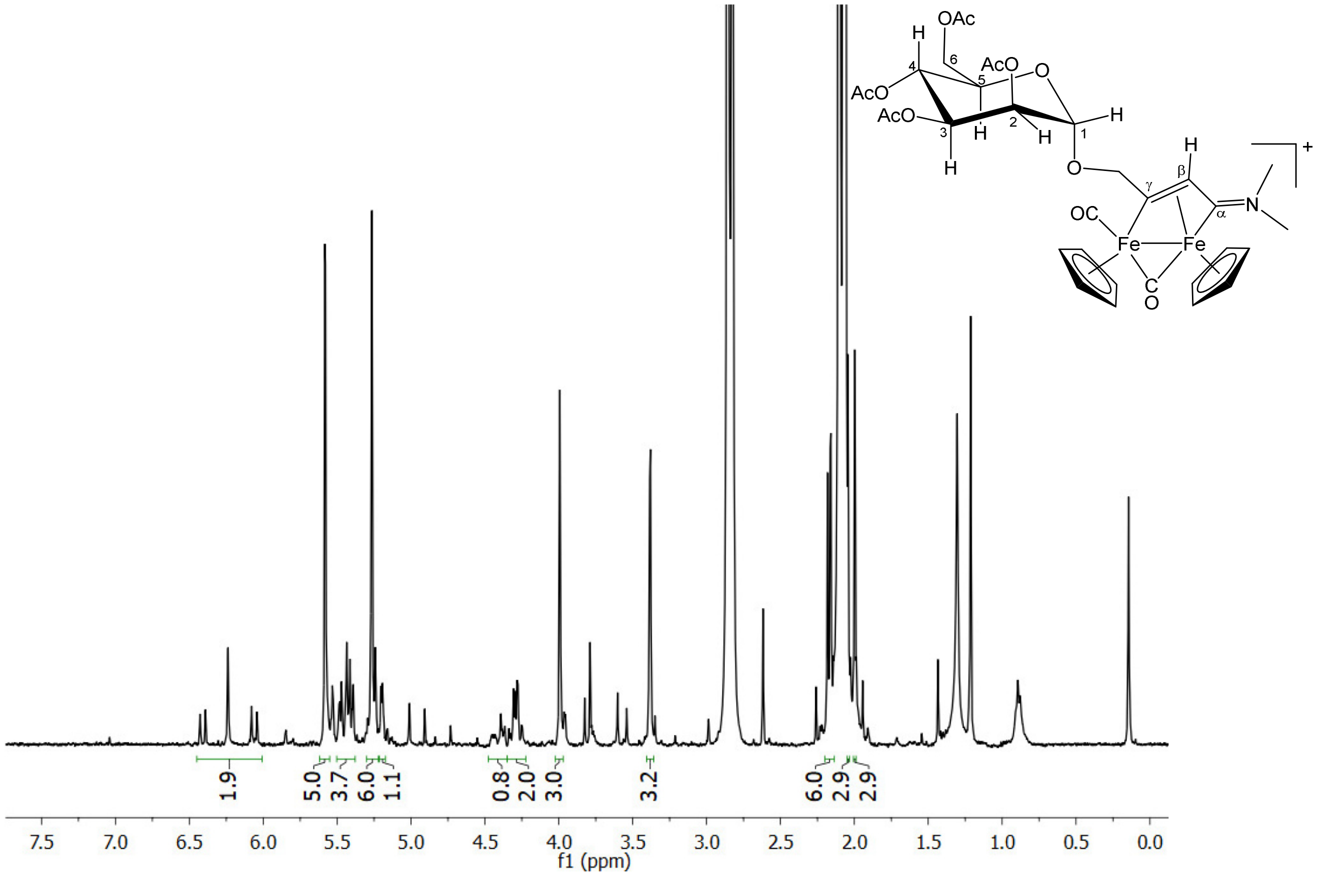

Figure S22. ^{13}C{^{1}H} NMR spectrum (101 MHz, acetone-d$_6$) of [**3a**]CF$_3$SO$_3$.

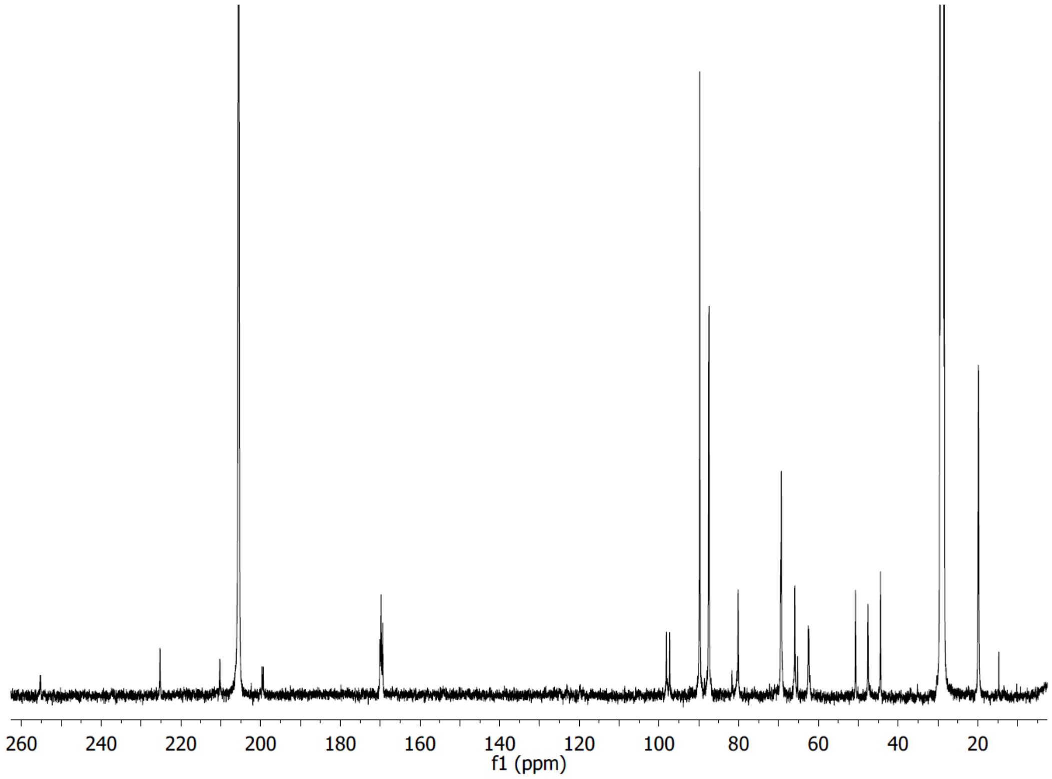

Figure S23. ^{1}H NMR spectrum (401 MHz, acetone-d$_6$) of [**3b**]CF_3SO_3.

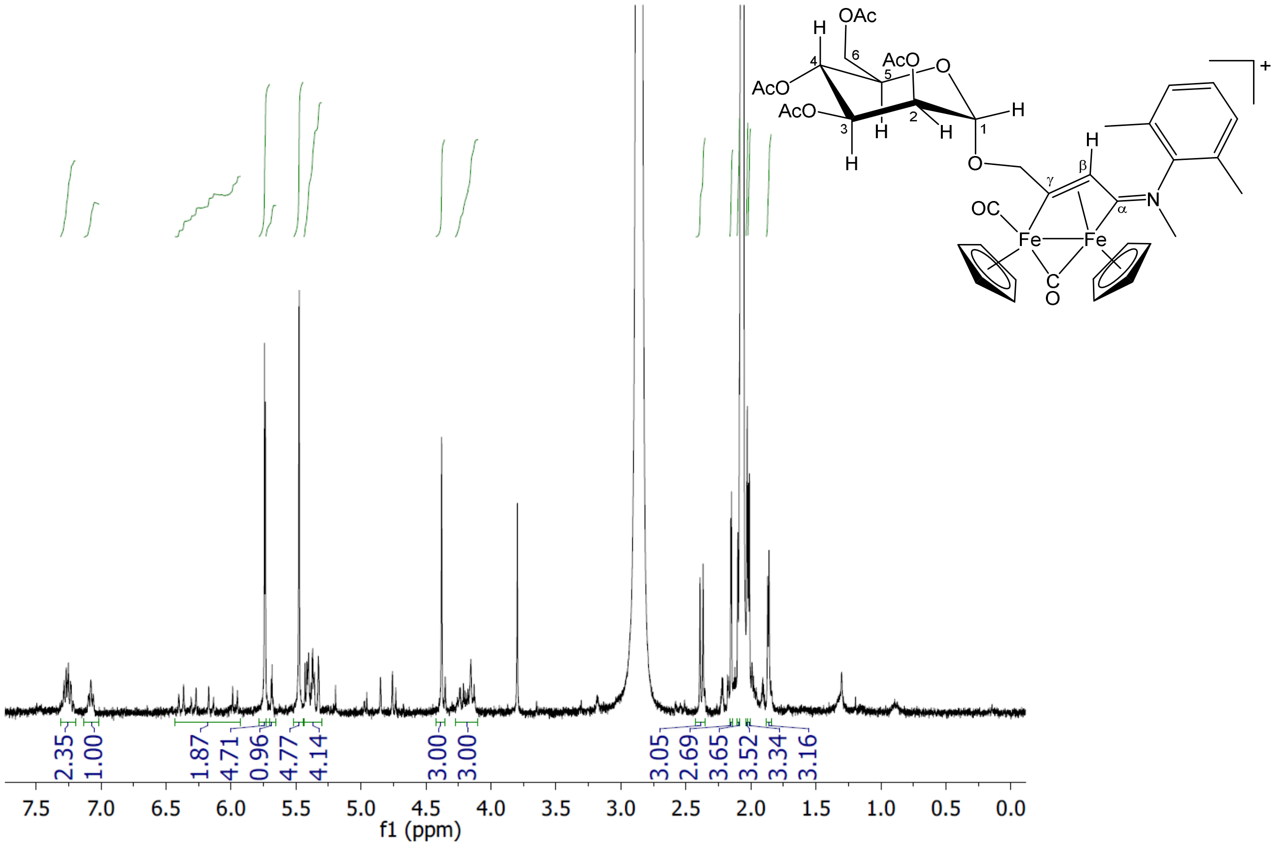

Figure S24. $^{13}C\{^{1}H\}$ NMR spectrum (101 MHz, acetone-d$_6$) of [**3b**]CF_3SO_3.

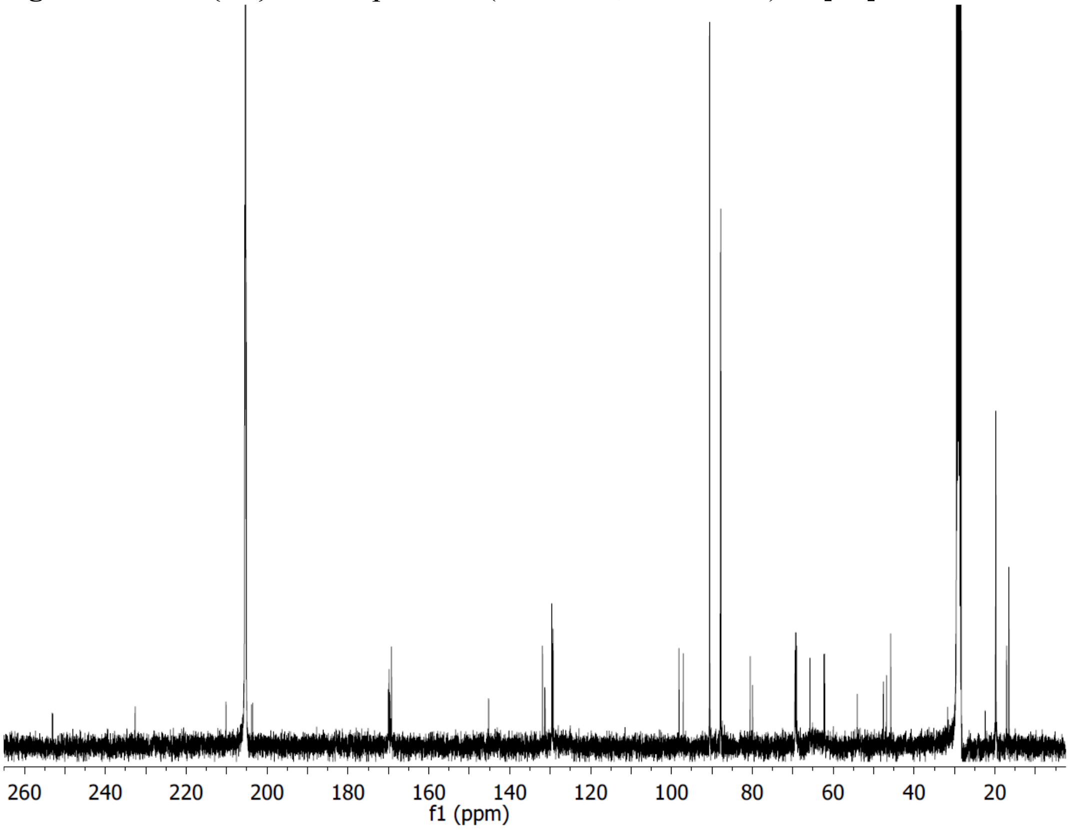

Figure S25. ^{1}H NMR spectrum (401 MHz, acetone-d$_6$) of [**4**]CF_3SO_3.

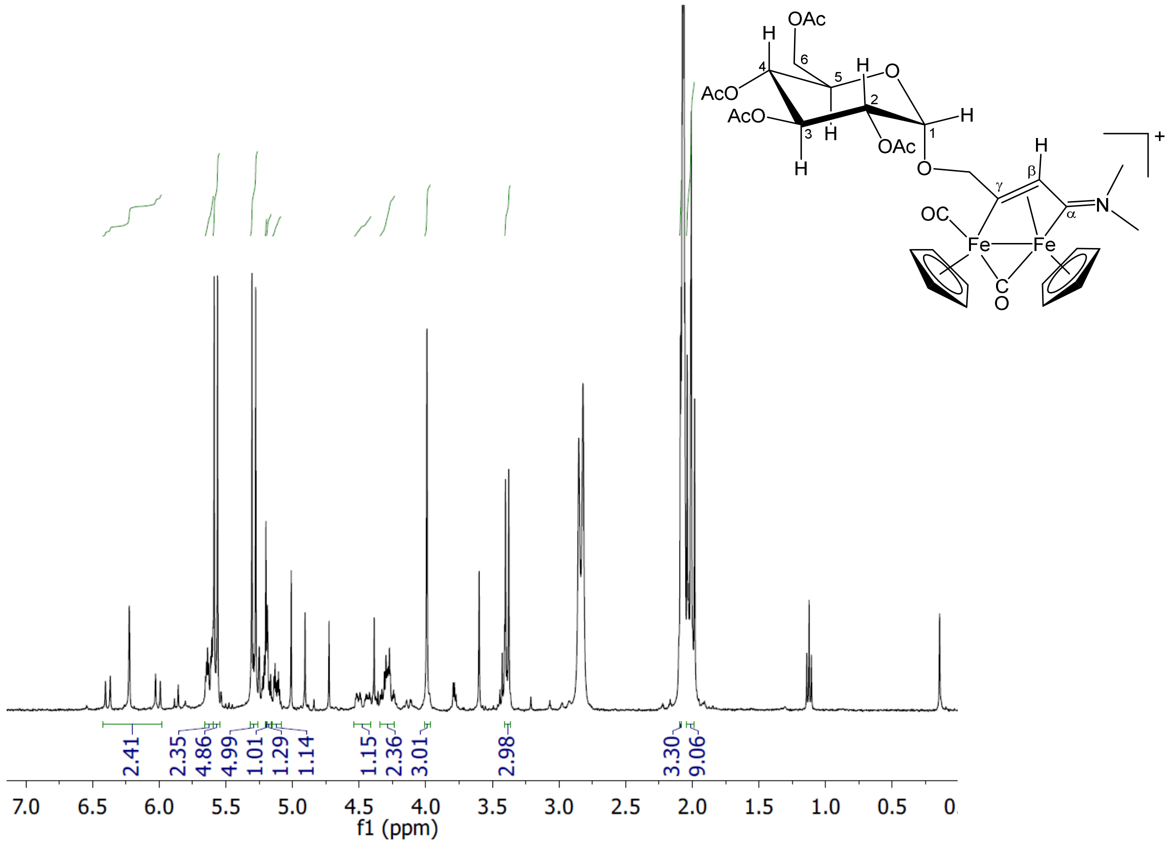

Figure S26. ^{13}C{^{1}H} NMR spectrum (101 MHz, acetone-d$_6$) of [**4**]CF_3SO_3.

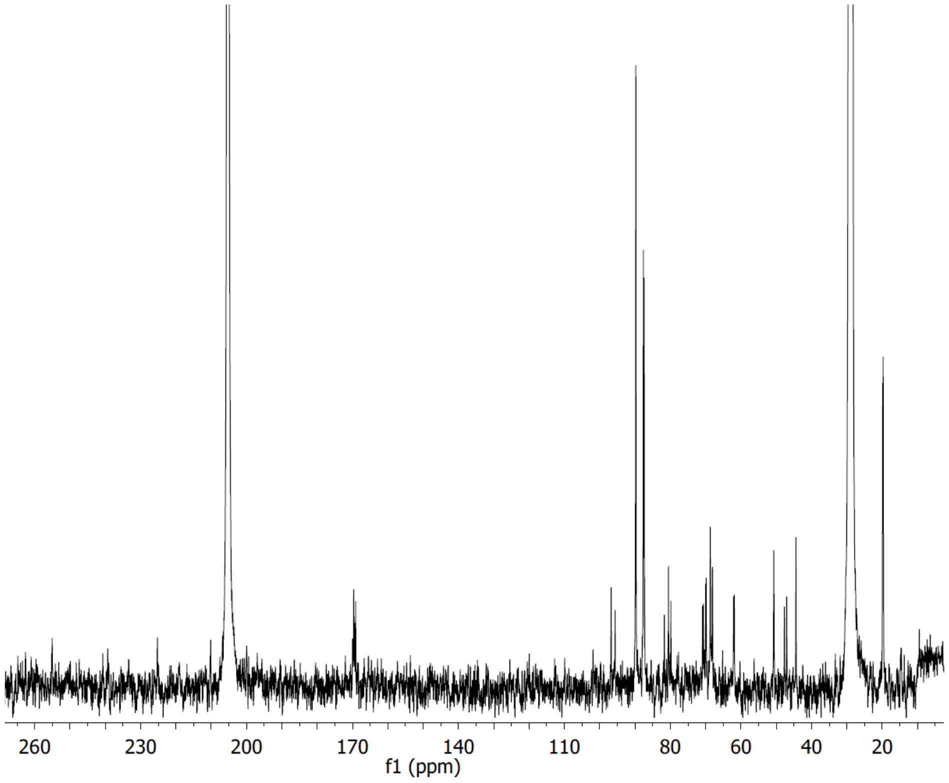

Figure S27. ^{1}H NMR spectrum (401 MHz, $CDCl_3$) of [**5a**]CF_3SO_3.

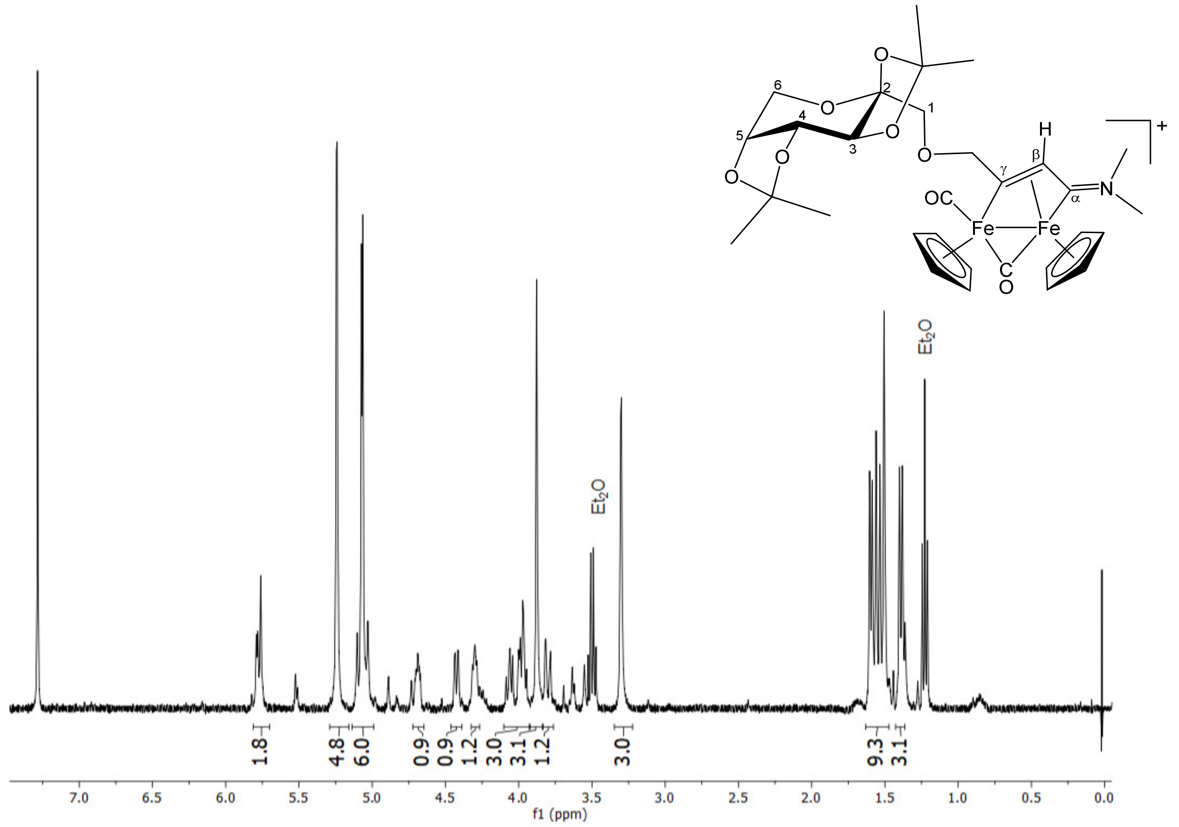

Figure S28. $^{13}C\{^{1}H\}$ NMR spectrum (101 MHz, $CDCl_3$) of [**5a**]CF_3SO_3.

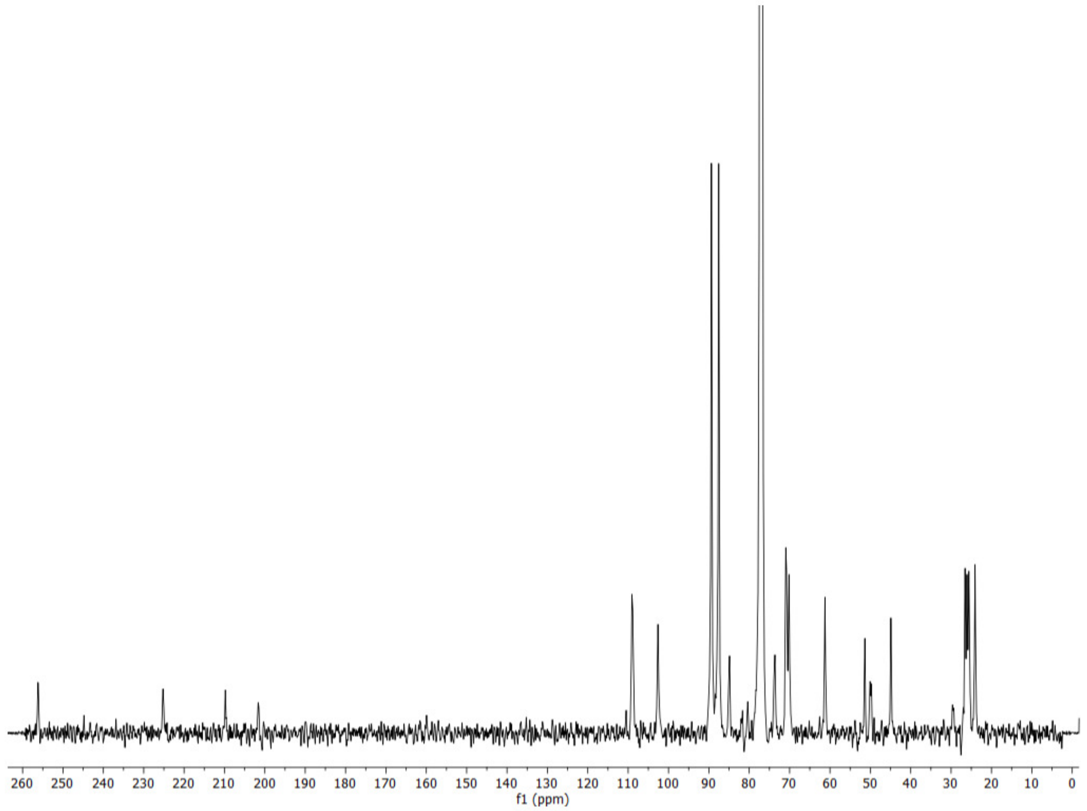

Figure S29. ^{1}H NMR spectrum (401 MHz, $CDCl_3$) of [**5b**]CF_3SO_3.

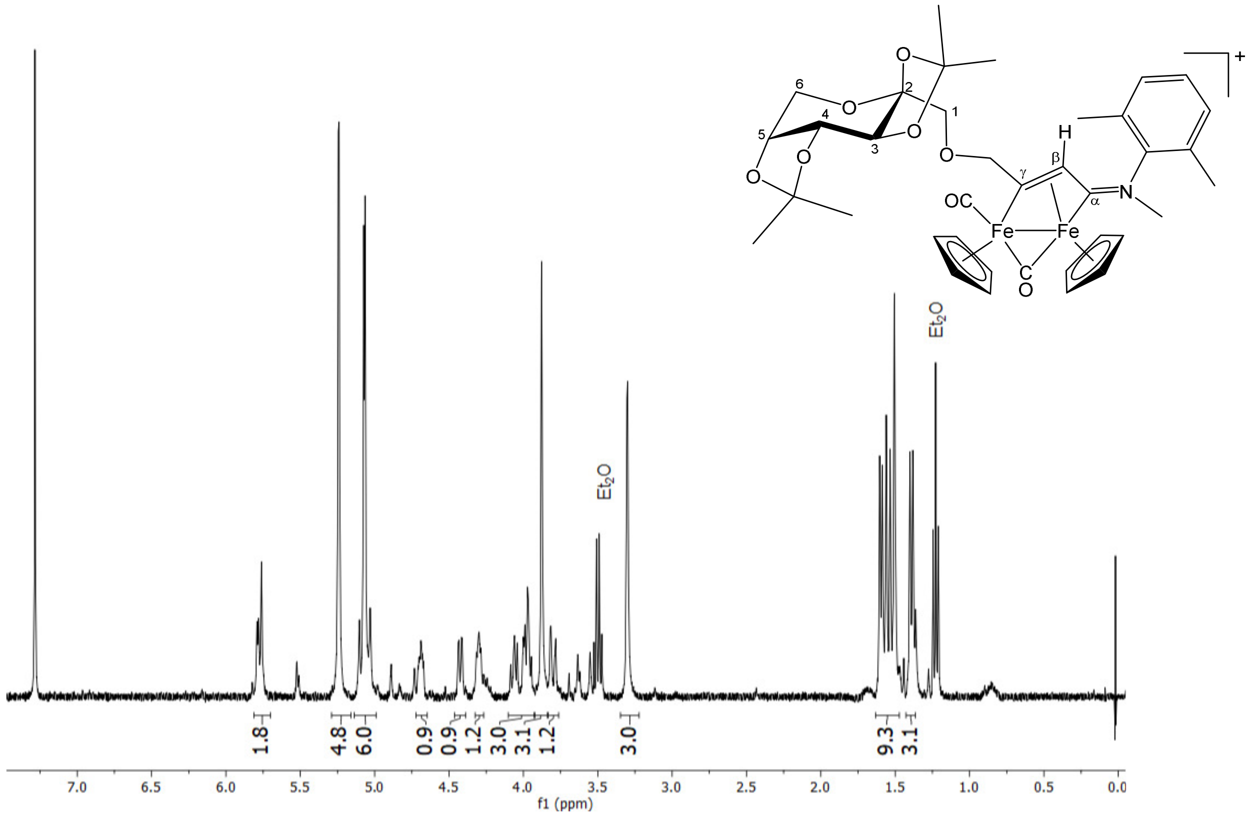

Figure S30. $^{13}C\{^{1}H\}$ NMR spectrum (101 MHz, $CDCl_3$) of [**5b**]CF_3SO_3.

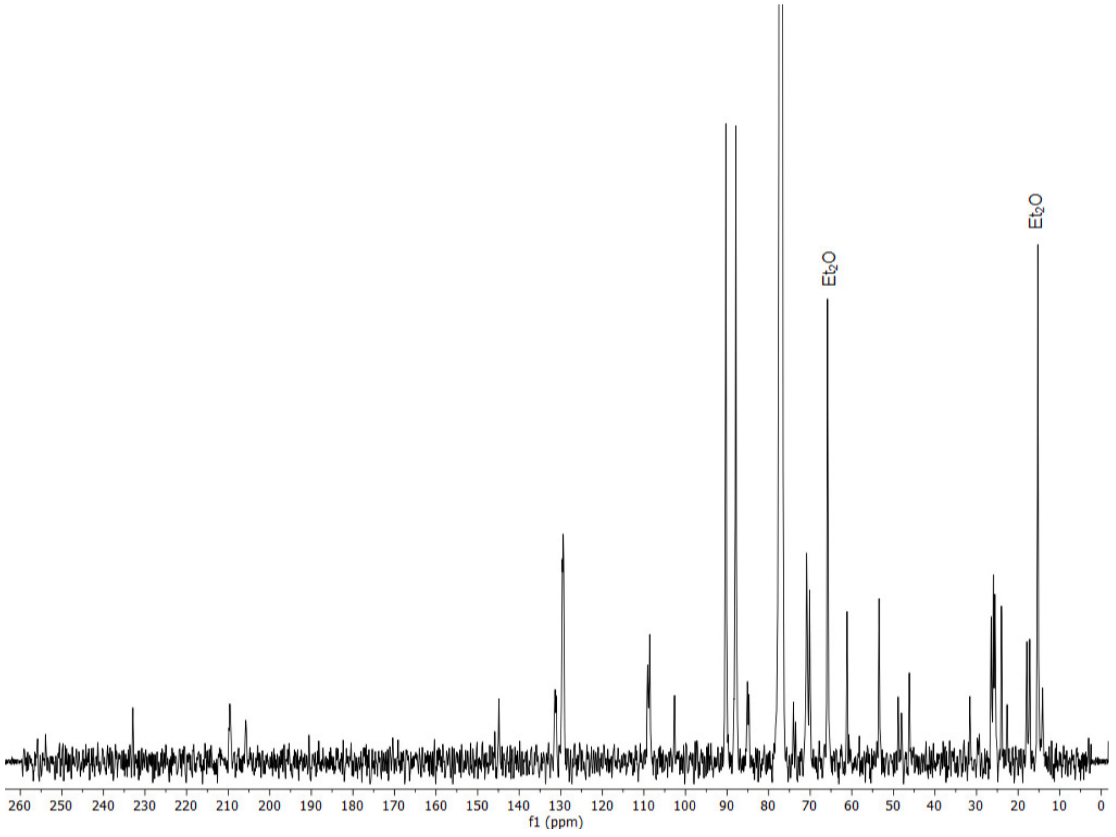

Figure S31. ^{1}H NMR spectrum (401 MHz, acetone-d_6) of [**6a**]CF_3SO_3.

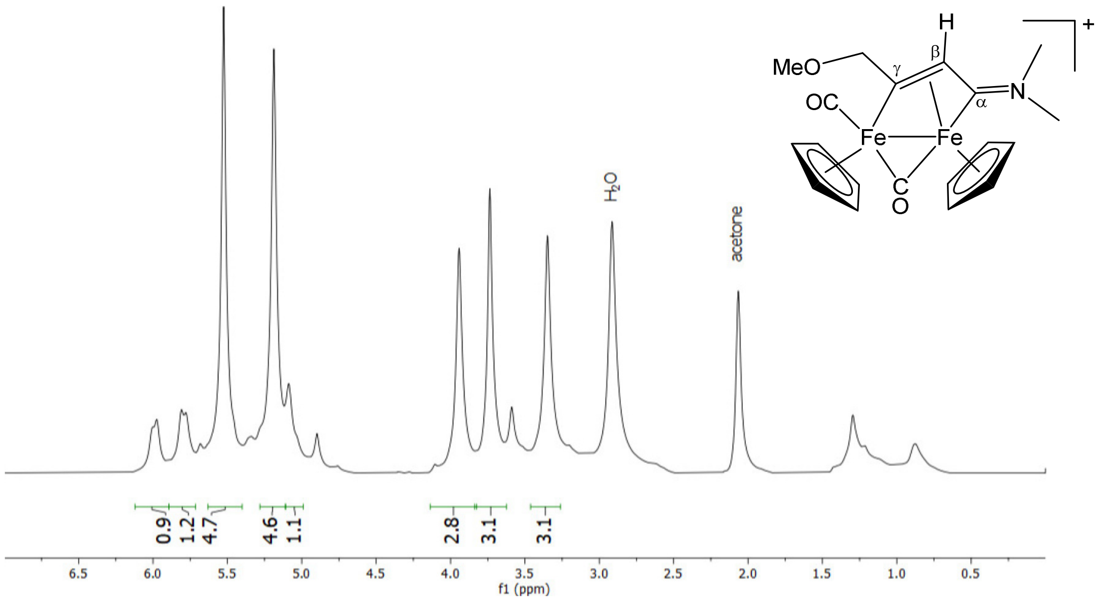

Figure S32. ^{13}C{^{1}H} NMR spectrum (101 MHz, acetone-d_6) of [**6a**]CF_3SO_3.

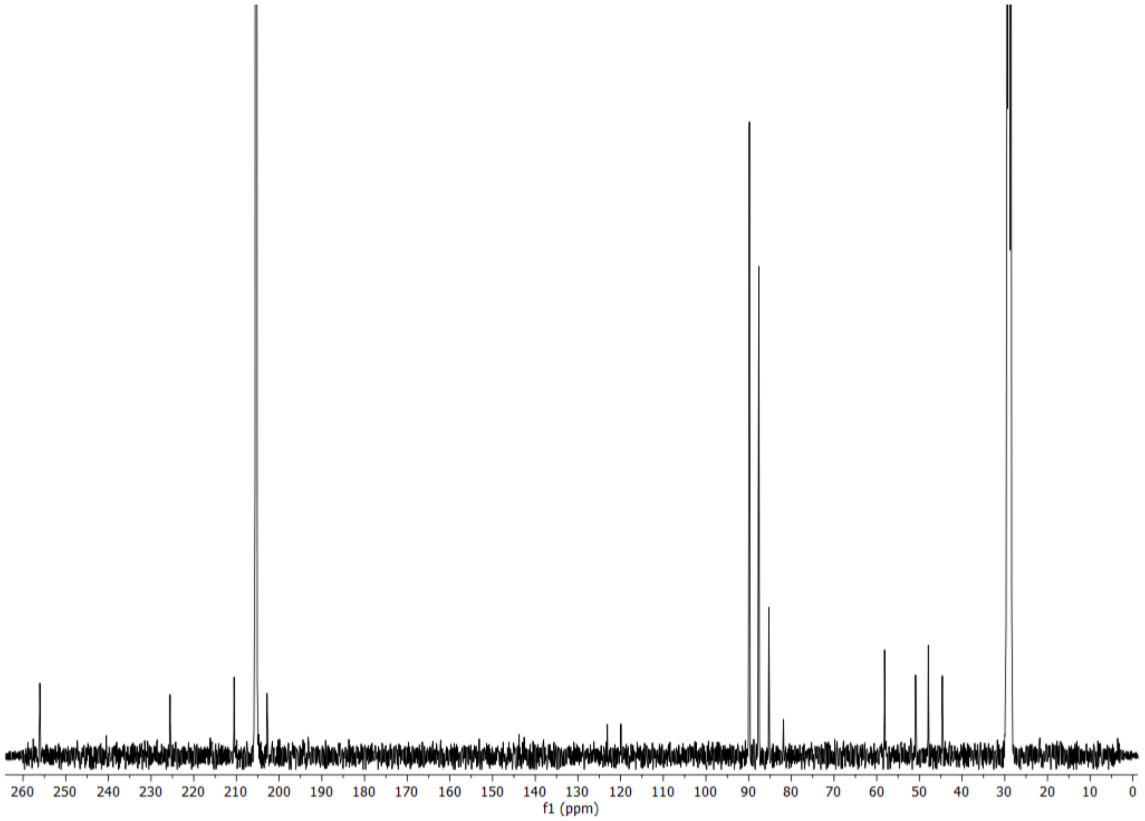

Figure S33. ^{1}H NMR spectrum (401 MHz, $CDCl_3$) of [**6b**]CF_3SO_3.

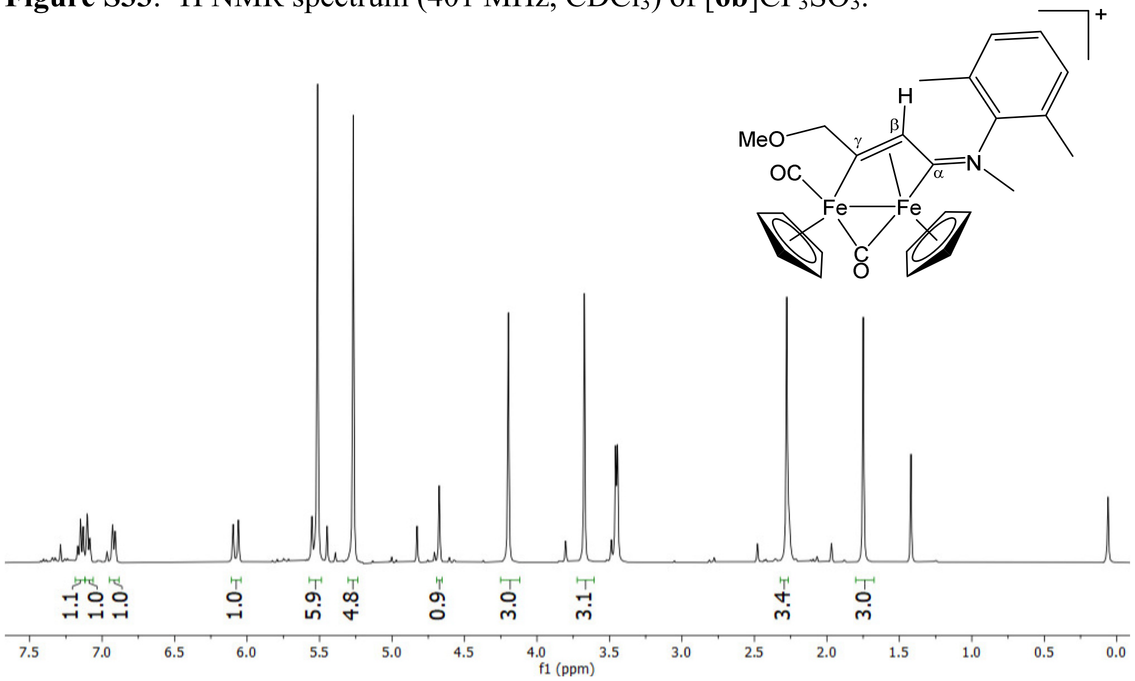

Figure S34. ^{13}C{^{1}H} NMR spectrum (101 MHz, $CDCl_3$) of [**6b**]CF_3SO_3.

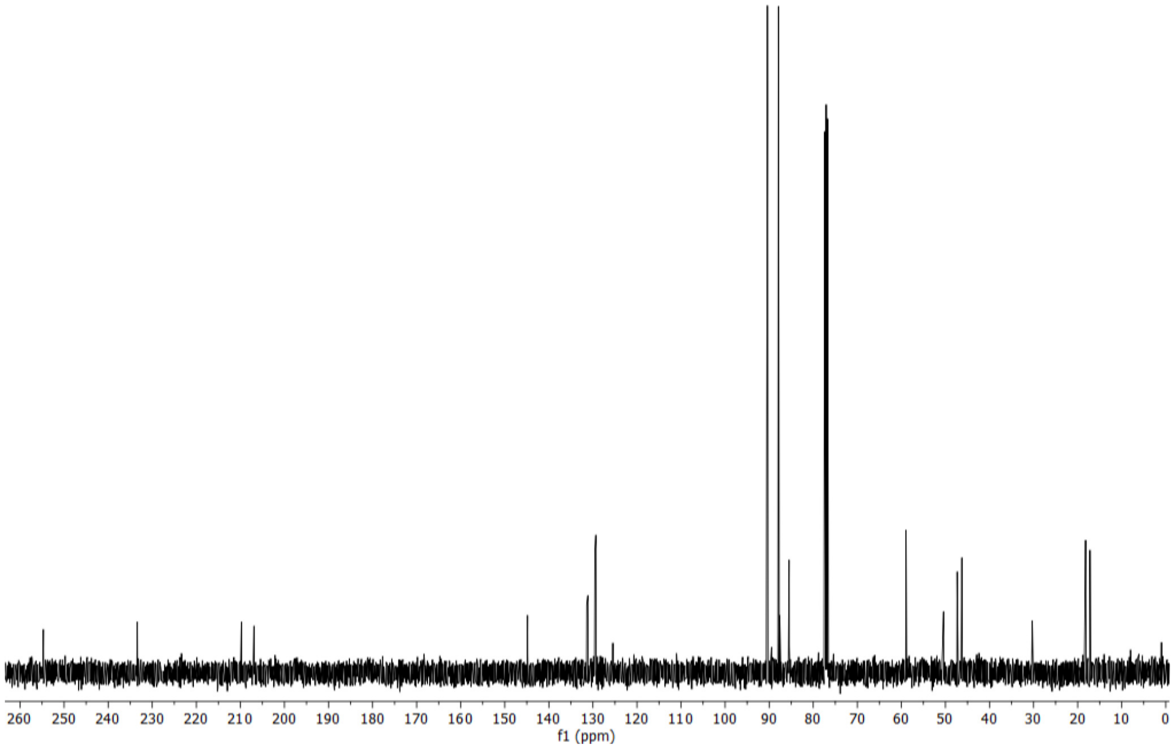

Figure S35. ^{1}H NMR spectrum (401 MHz) of [**2**]CF_3SO_3 in D_2O.

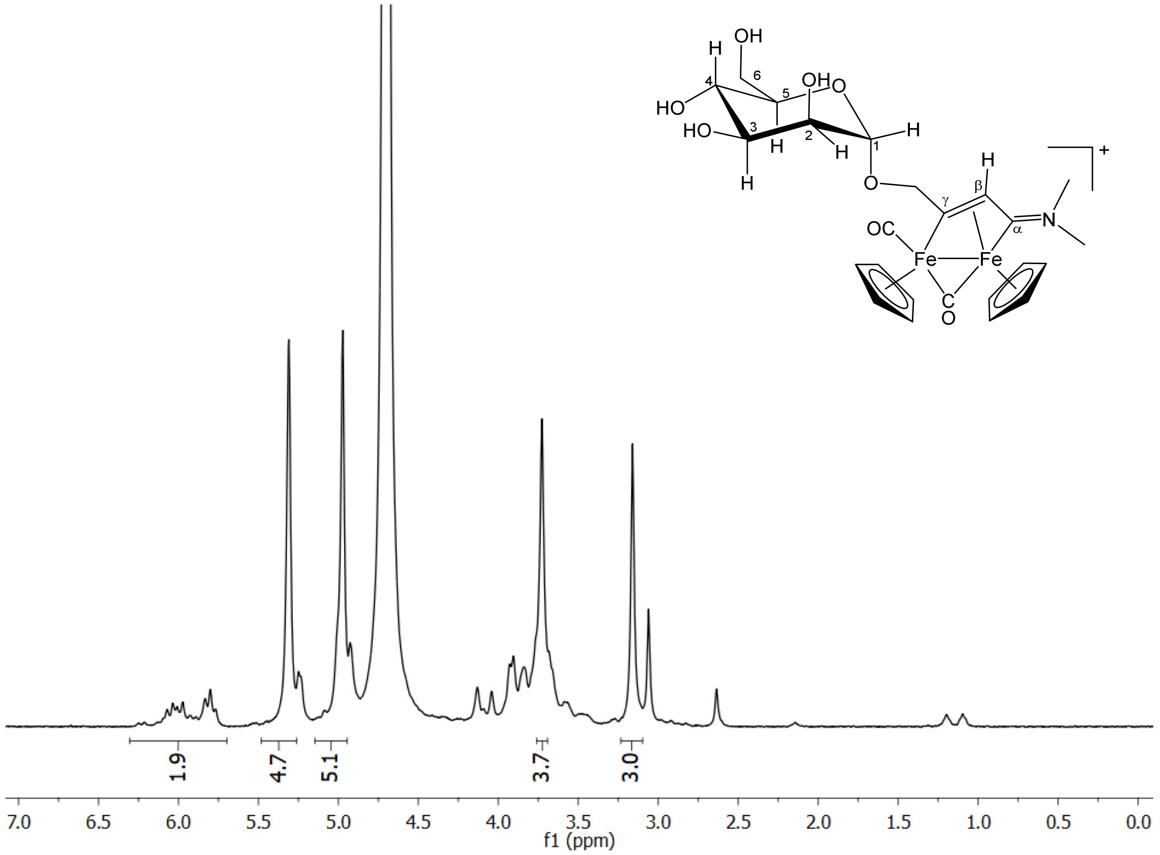

Figure S36. ^{1}H NMR spectrum (401 MHz) of [**3a**]CF_3SO_3 in D_2O.

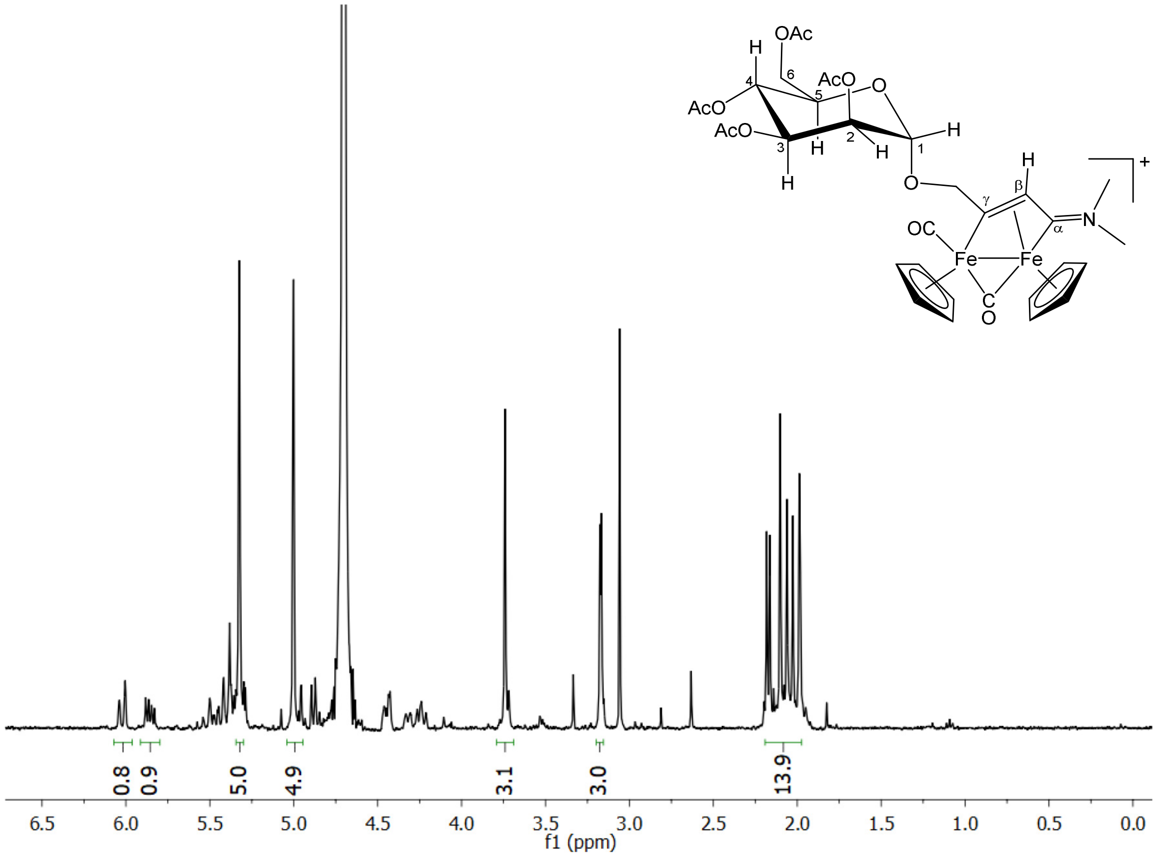

Figure S37. ¹H NMR spectrum (401 MHz) of [**3b**]CF_3SO_3 in D_2O/DMSO-d_6 (2:1 v/v).

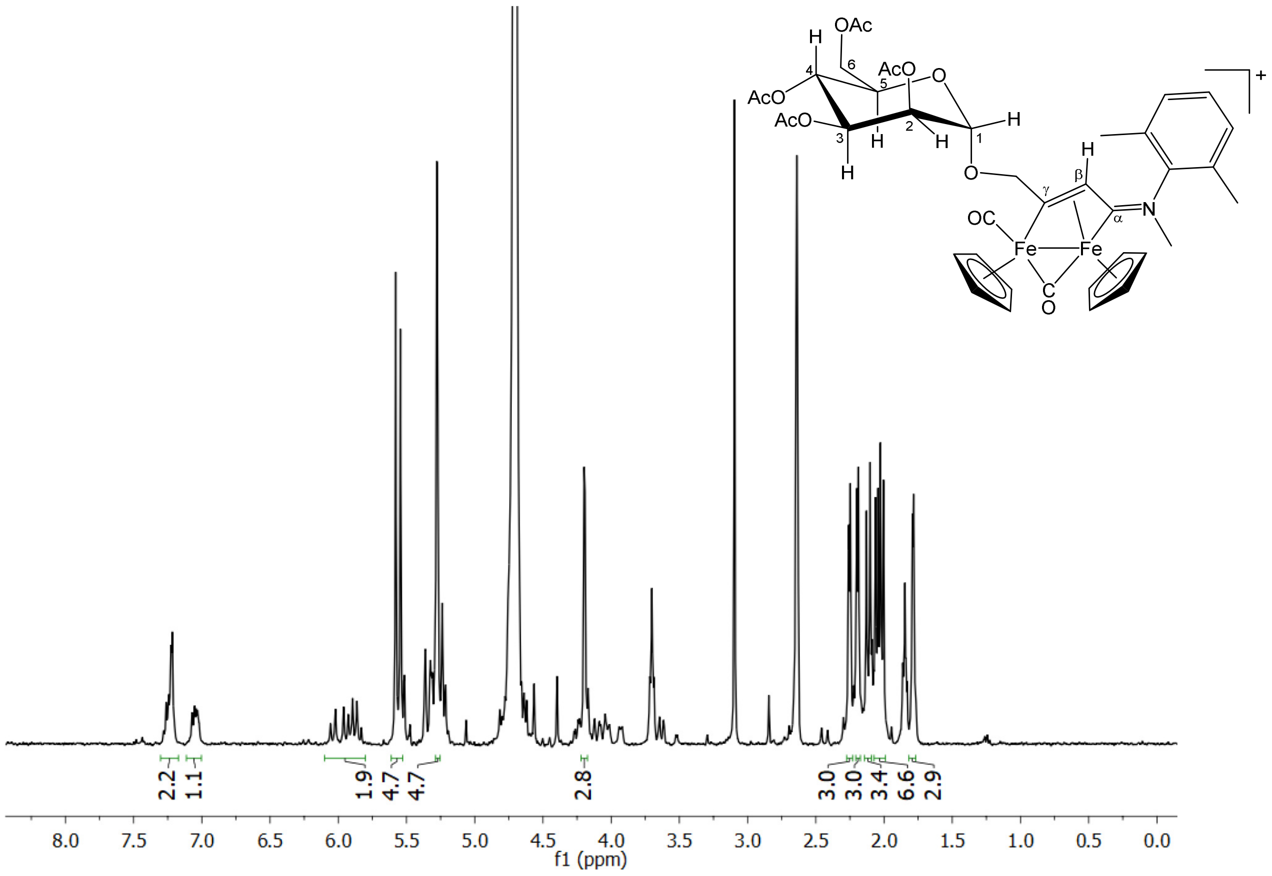

Figure S38. ¹H NMR spectrum (401 MHz) of [**4**]CF_3SO_3 in D_2O/DMSO-d_6 (2:1 v/v).

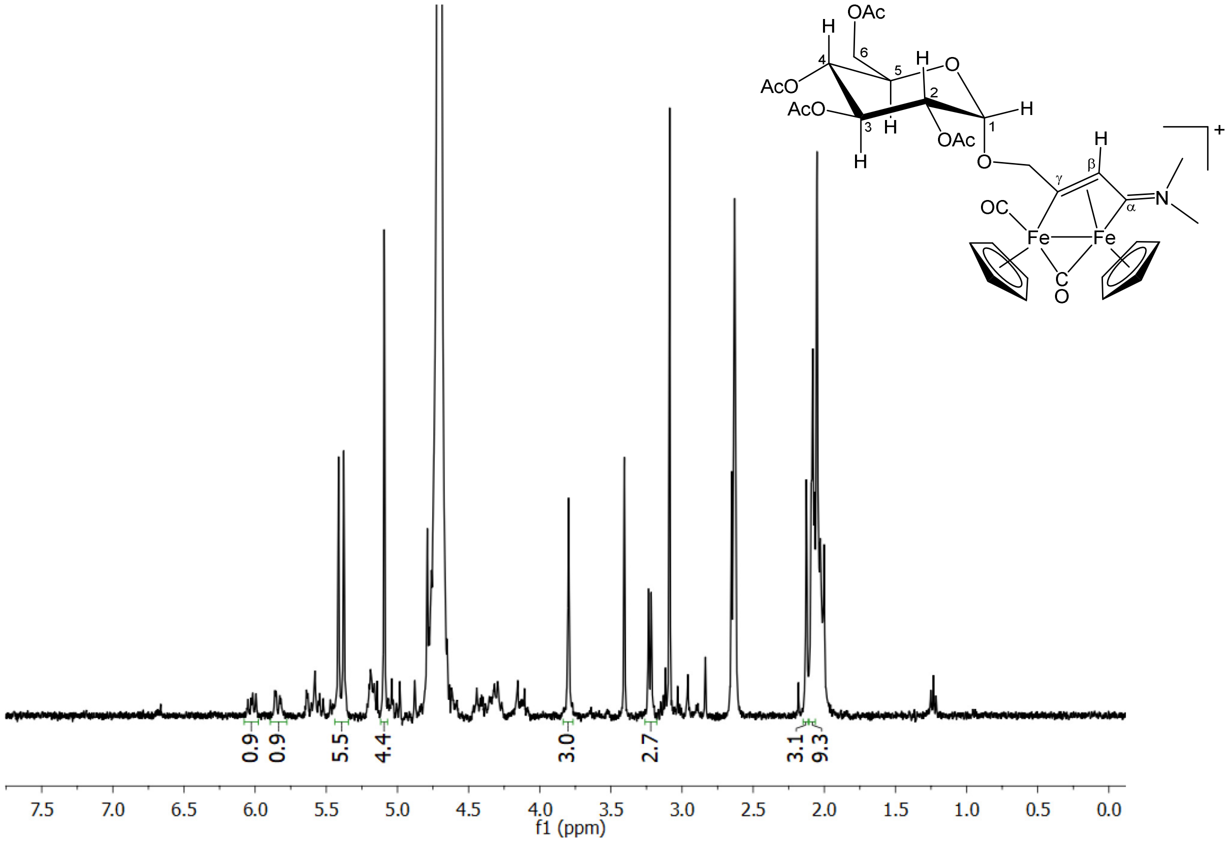

Figure S39. ^{1}H NMR spectrum (401 MHz) of [**5a**]CF_3SO_3 in D_2O/DMSO-d_6 (2:1 v/v).

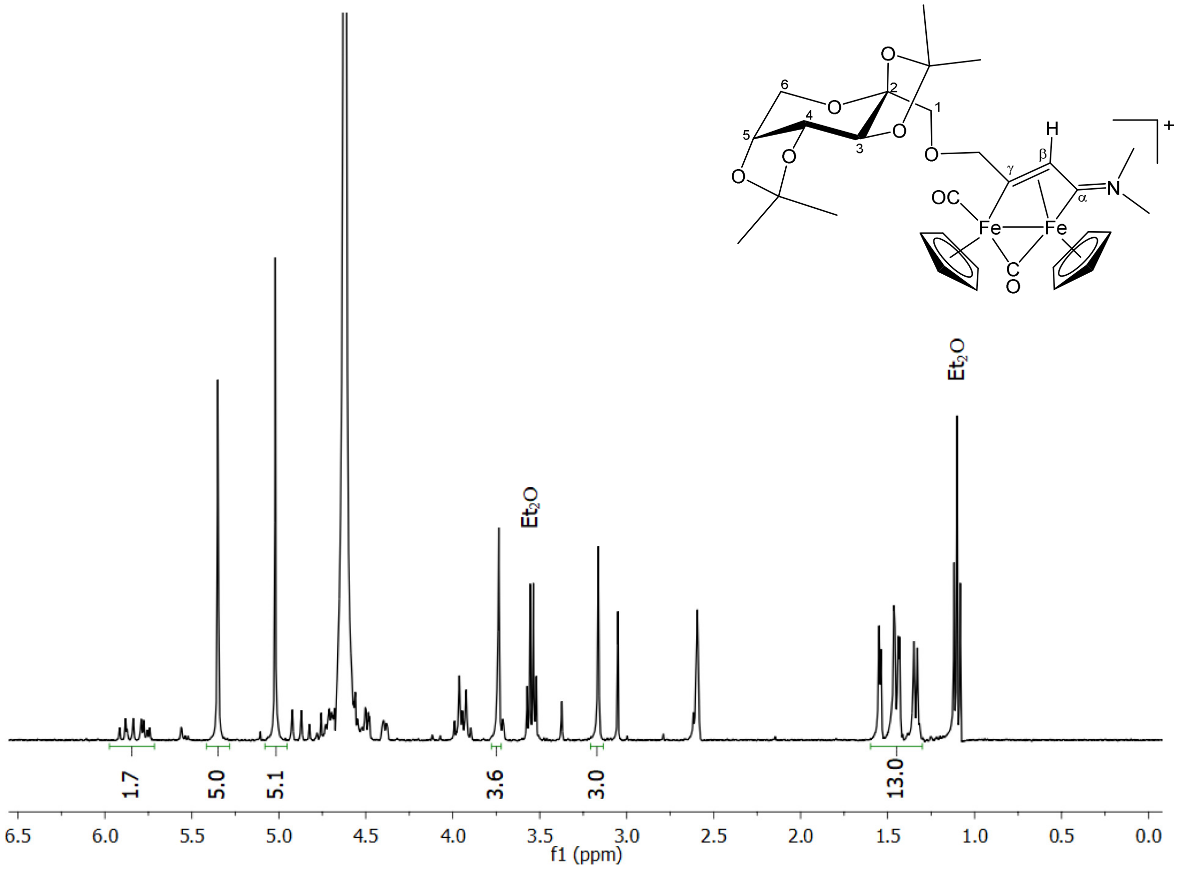

Figure S40. ^{1}H NMR spectrum (401 MHz) of [**5b**]CF_3SO_3 in D_2O/DMSO-d_6 (2:1 v/v).

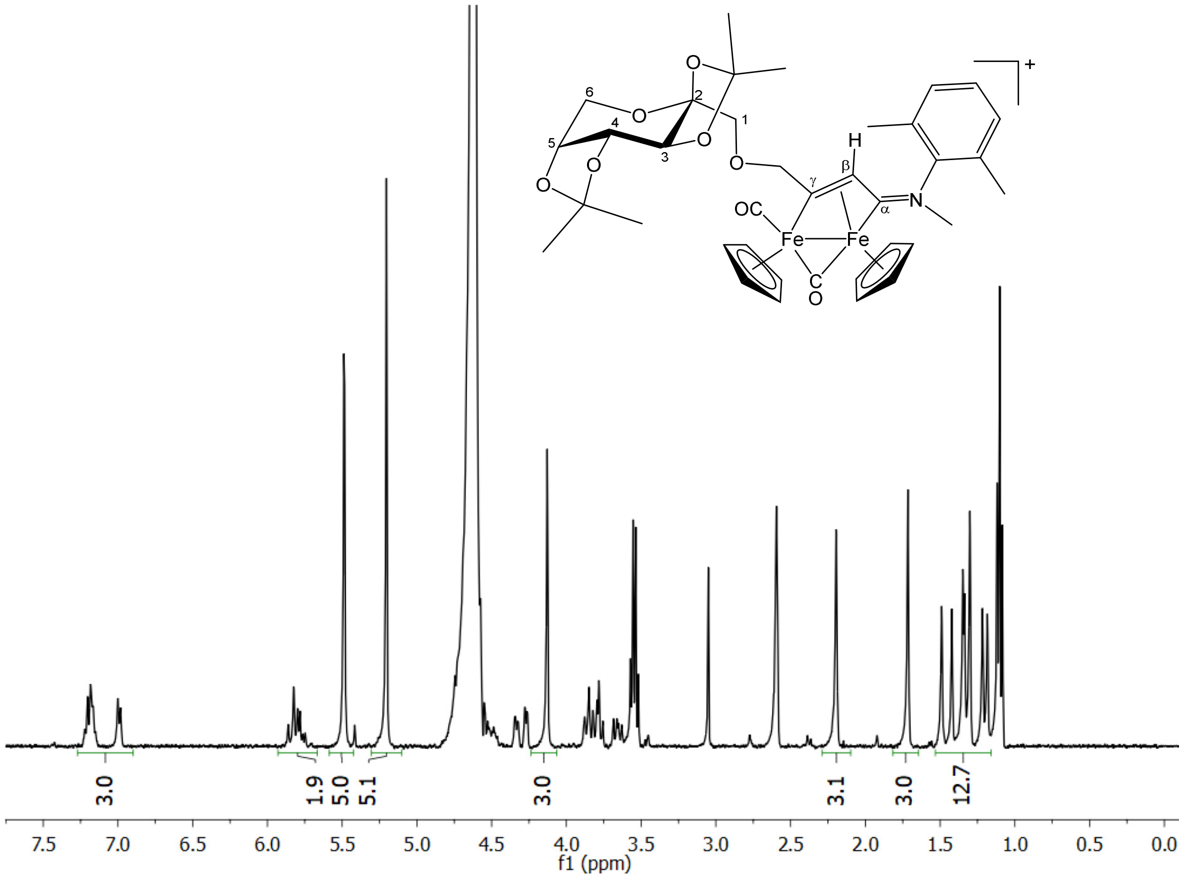

Figure S41. ^{1}H NMR spectrum (401 MHz) of [**6a**]CF_3SO_3 in D_2O/DMSO-d_6 (2:1 v/v).

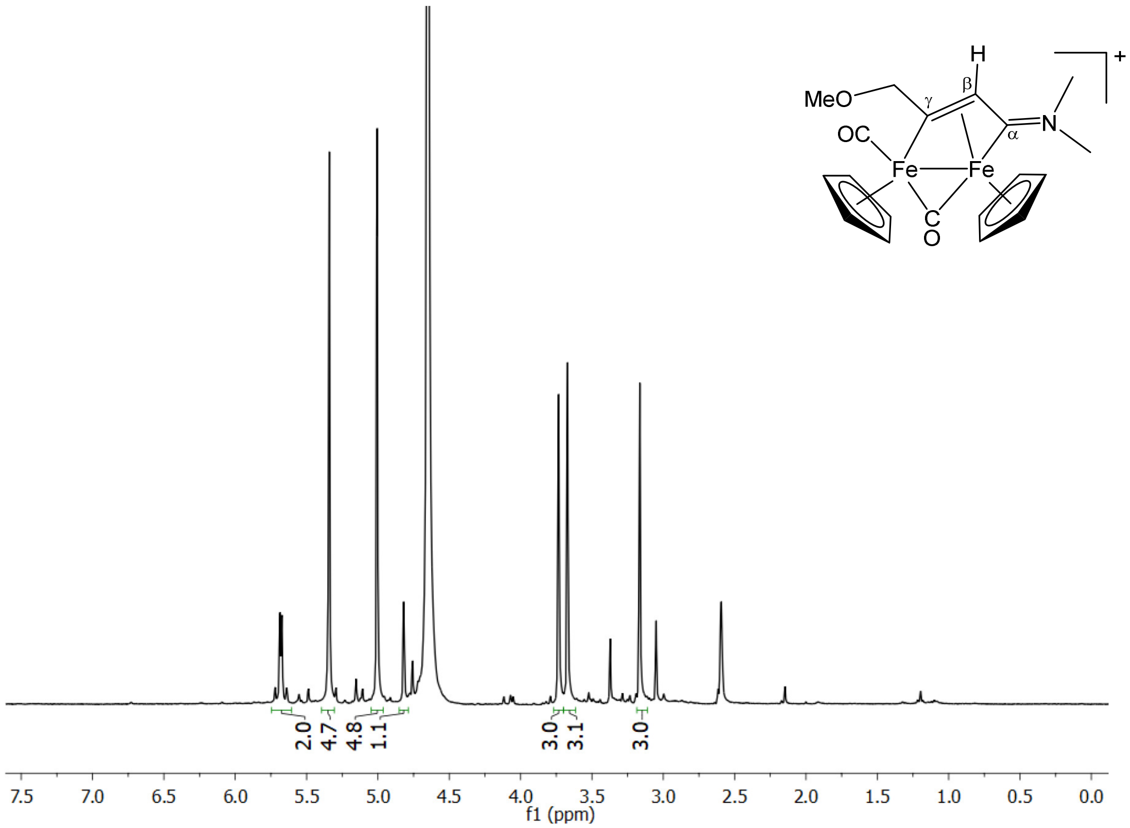

Figure S42. ^{1}H NMR spectrum (401 MHz) of [**6b**]CF_3SO_3 in D_2O/DMSO-d_6 (2:1 v/v).

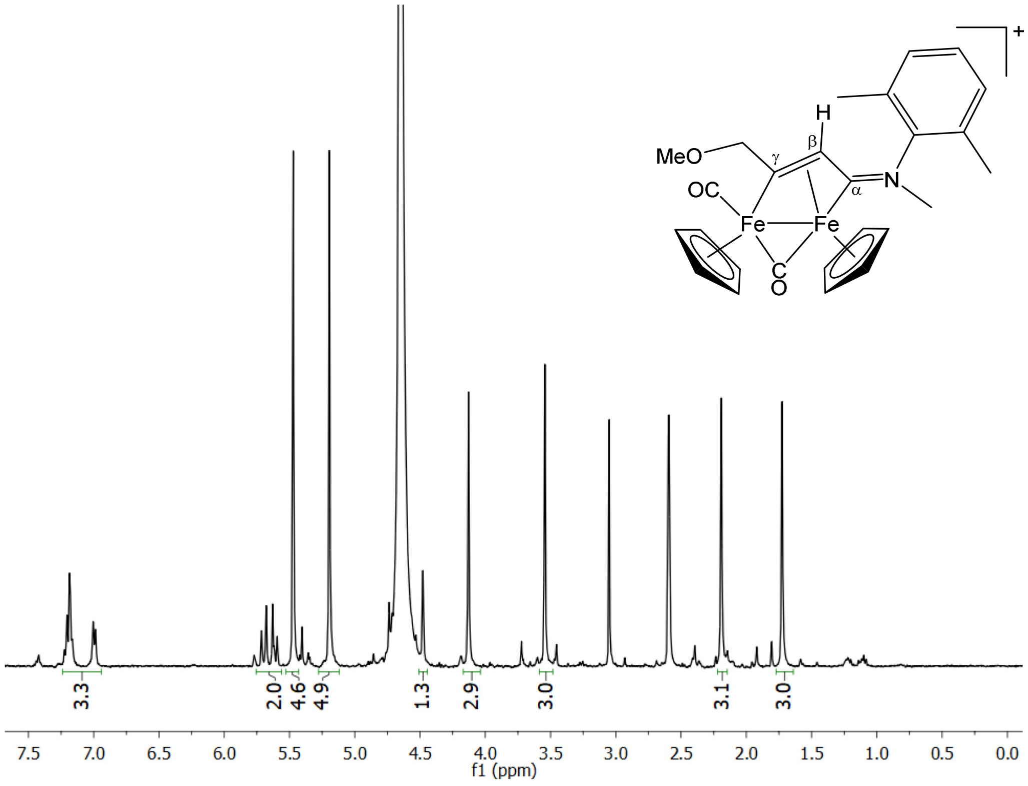

Figure S43. ESI-MS spectrum of [**2**]CF_3SO_3 before (A) and after (B) 72 hours in DMSO-RPMI mixture. Peaks at 506.53, 544.49, 1012.05 and 1034.03 m/z are present in the procedural blanks. Zoomed ESI-MS spectra of [**2**]CF_3SO_3 in the range 380-500 m/z highlighting the peaks at 410.0 and 424.0 m/z before (C) and after (D) 72 hours in DMSO-RPMI mixture

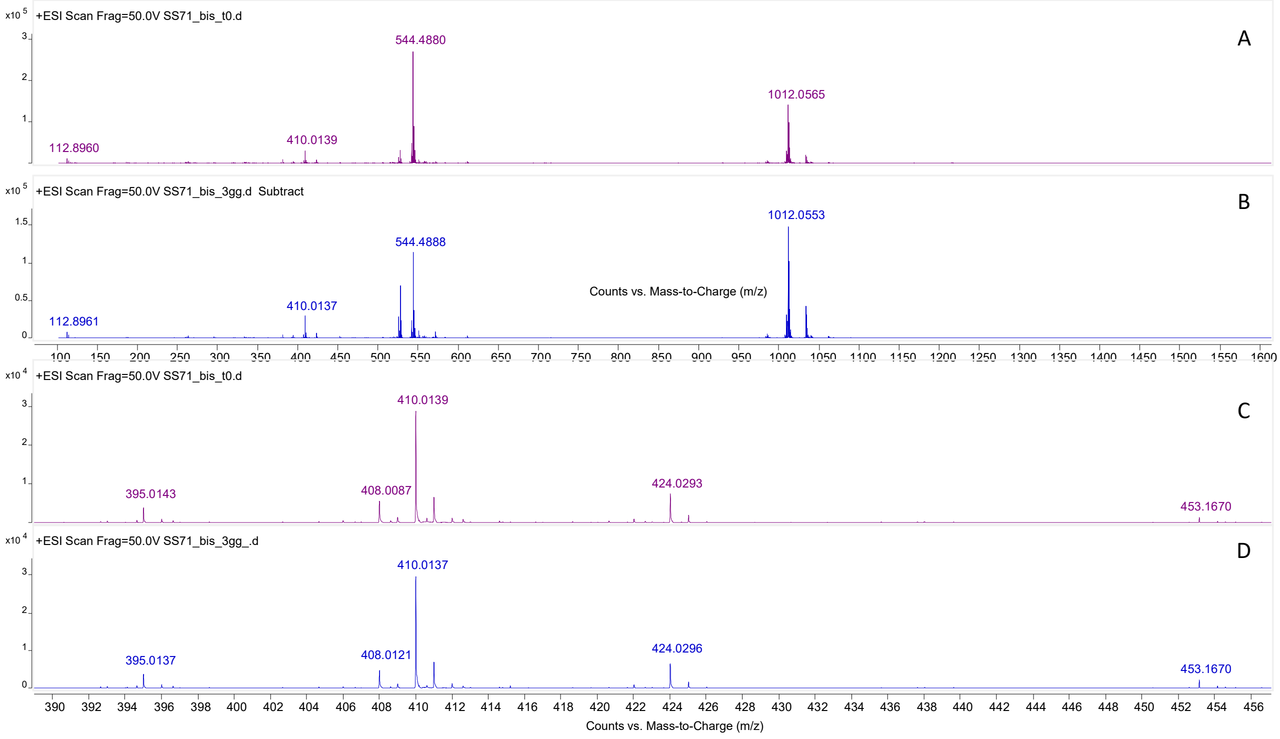

Figure S44. ESI-MS spectrum of [**3a**]CF_3SO_3 after 72 hours in DMSO-RPMI mixture.

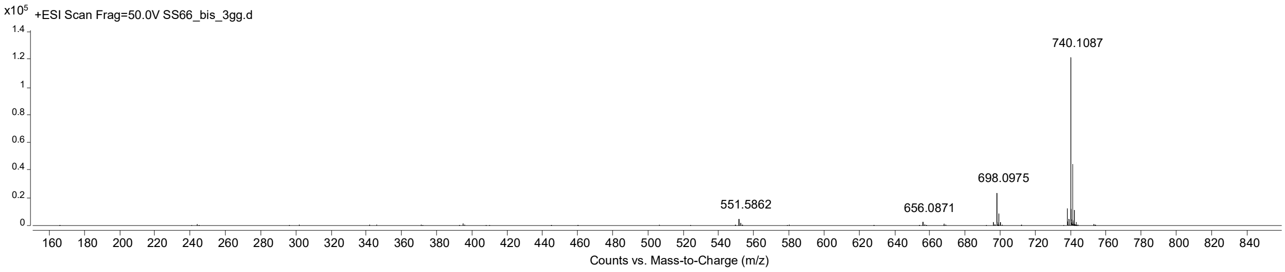

Figure S45. ESI-MS spectrum of [**3b**]CF_3SO_3 after 72 hours in DMSO-RPMI mixture.

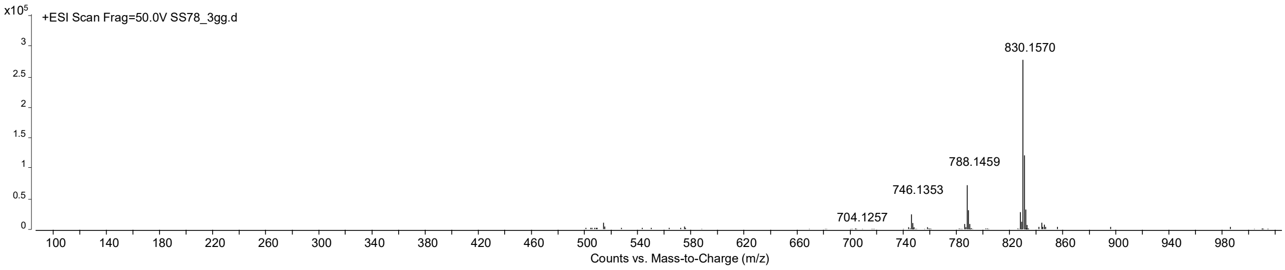

Figure S46. ESI-MS spectrum of [**4**]CF_3SO_3 after 72 hours in DMSO-RPMI mixture.

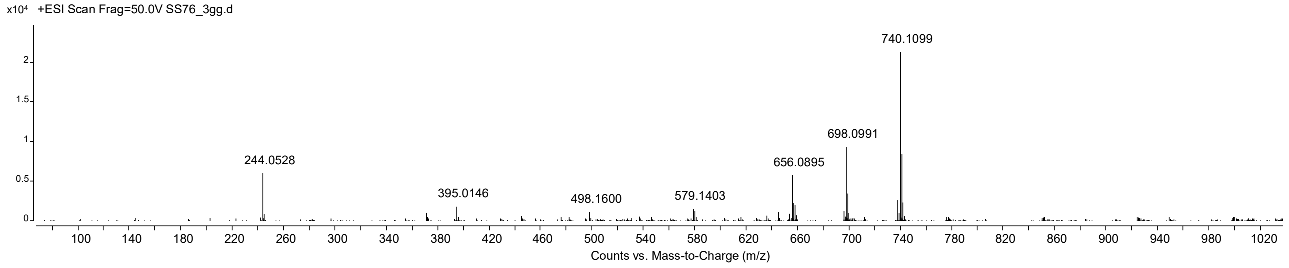

Figure S47. ESI-MS spectrum of [**5a**]CF_3SO_3 after 72 hours in DMSO-RPMI mixture.

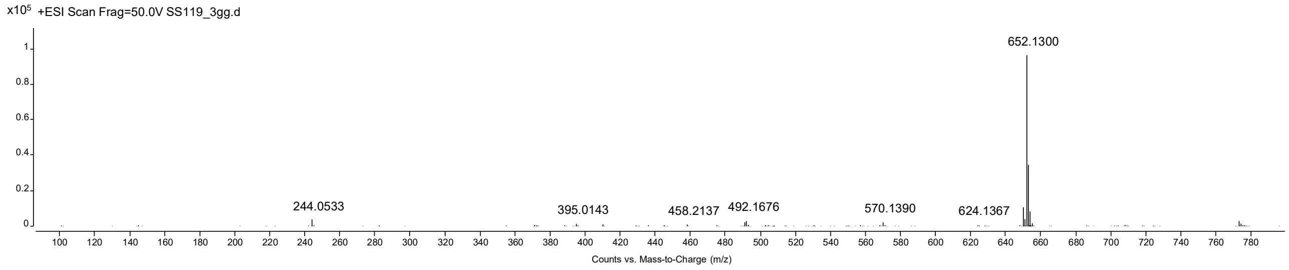

Figure S48. ESI-MS spectrum of [**5b**]CF_3SO_3 after 72 hours in DMSO-RPMI mixture.

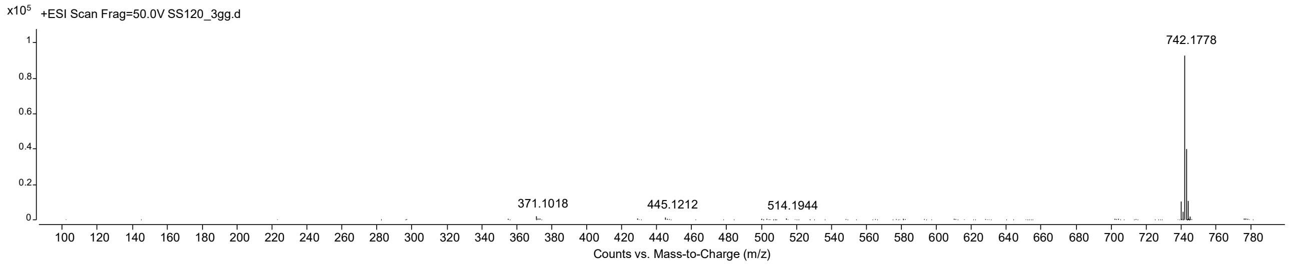

Figure S49. ESI-MS spectrum of [**6a**]CF_3SO_3 after 72 hours in DMSO-RPMI mixture.

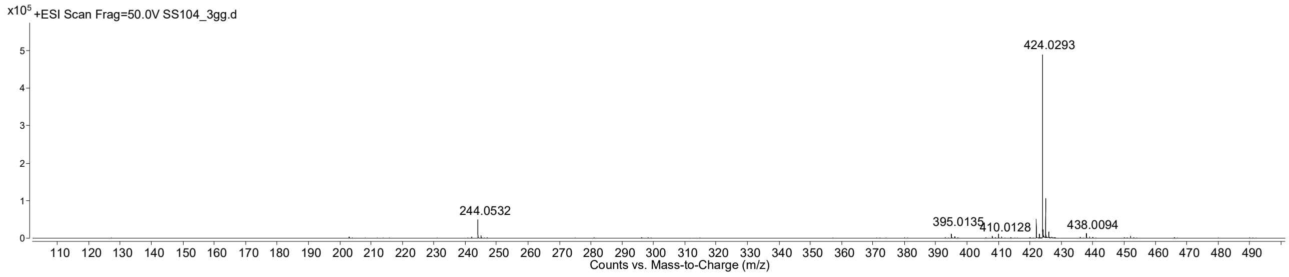

Figure S50. ESI-MS spectrum of [**6b**]CF_3SO_3 after 72 hours in DMSO-RPMI mixture.

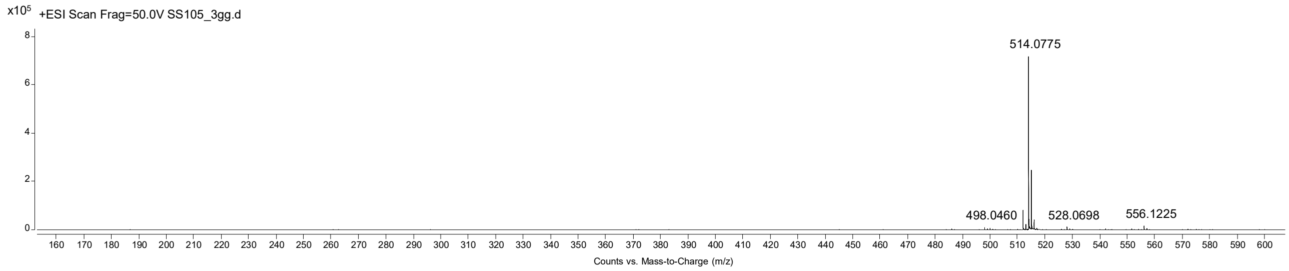

Figure S51. IC_{50} plots for CT26 cell line treated with diiron complexes over 48 h.

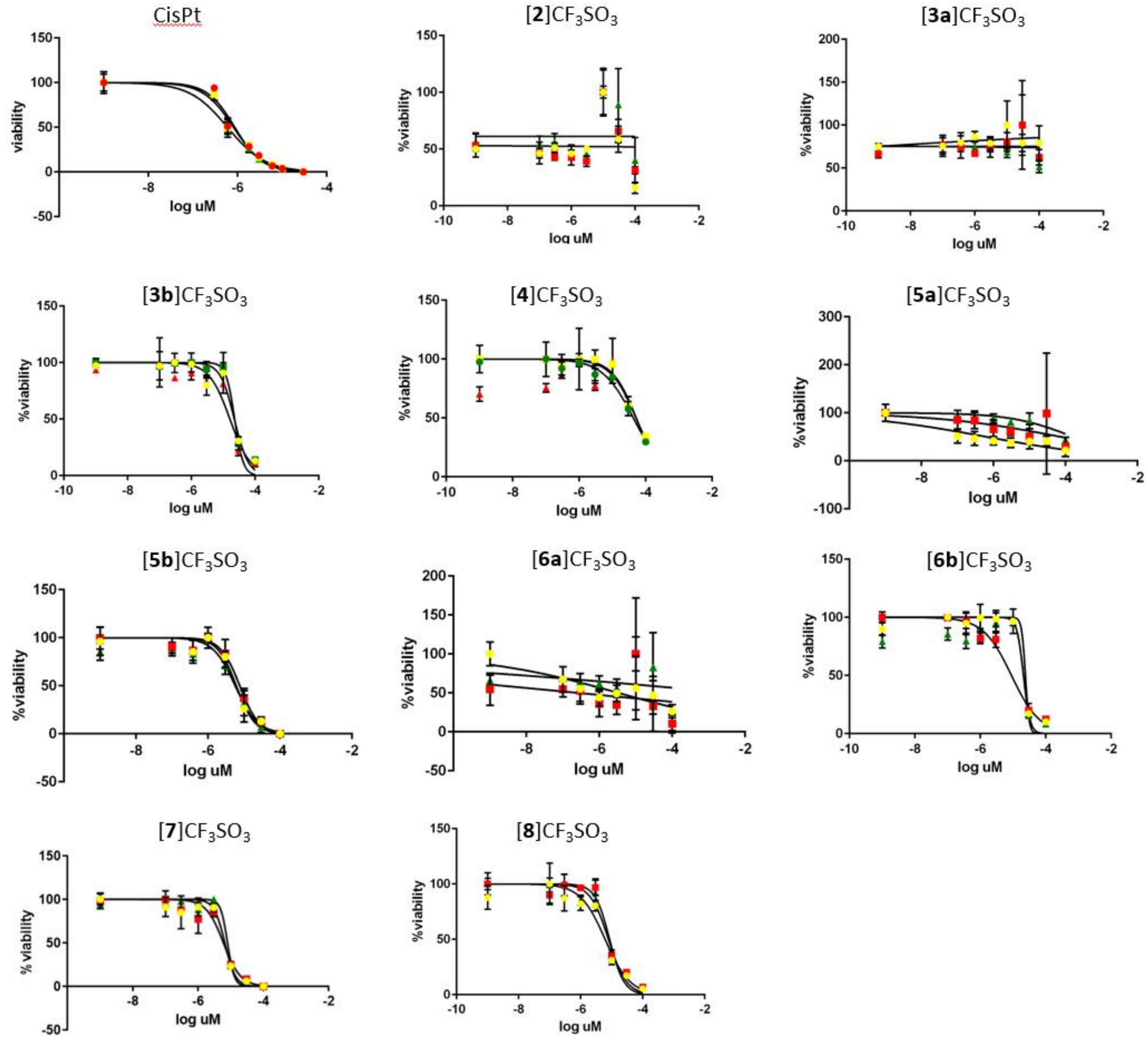

Figure S52. IC_{50} plots for U87 cell line treated with diiron complexes over 48 h.

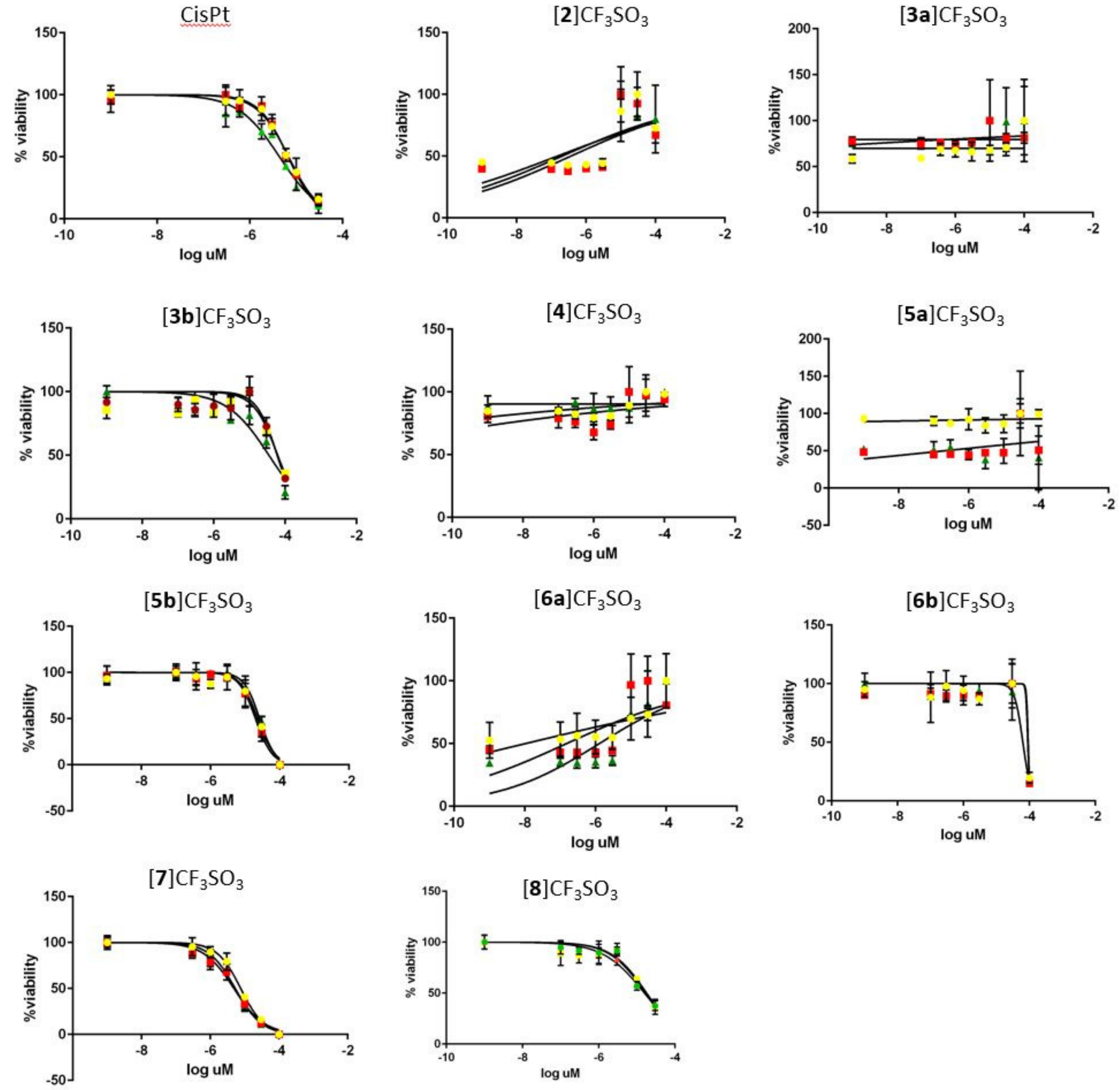

Figure S53. IC_{50} plots for MCF7 cell line treated with diiron complexes over 48 h.

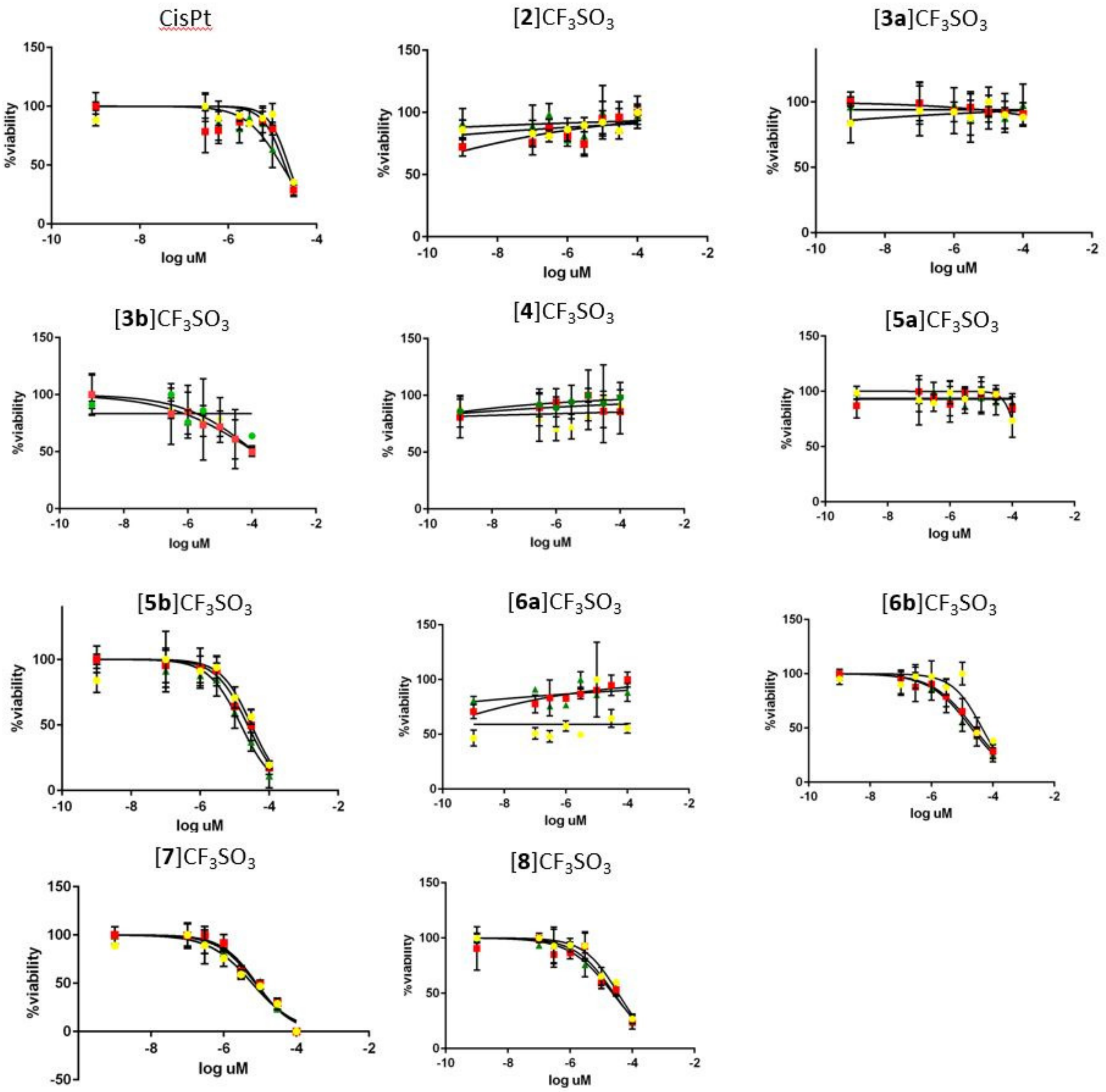

Figure S54. IC_{50} plots for RPE1 cell line treated with diiron complexes over 48 h.

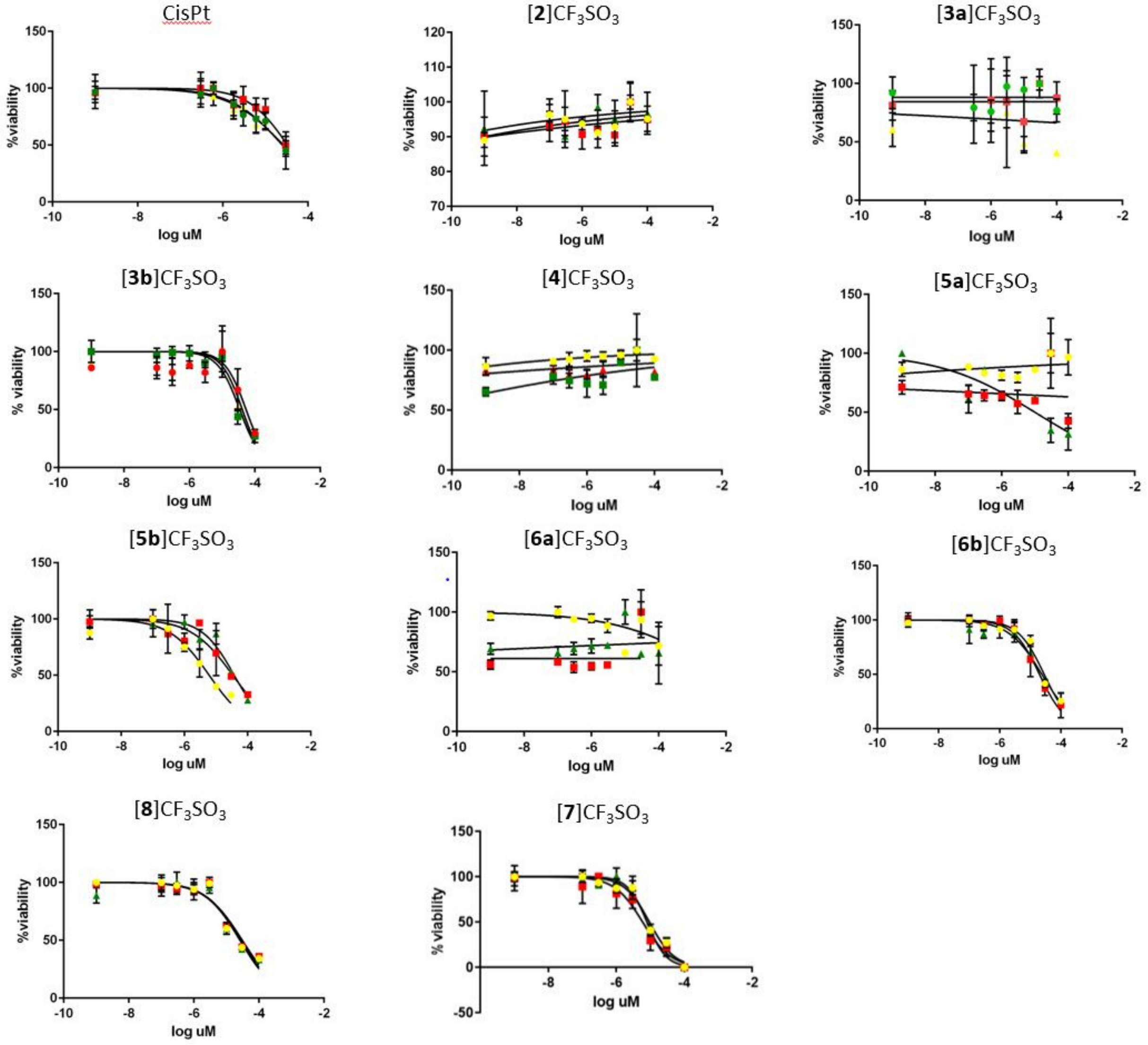

Figure S55. IC_{50} plots for CT26 cell line treated with selected diiron complexes over 48 h, in the presence and in the absence of glucose, respectively.

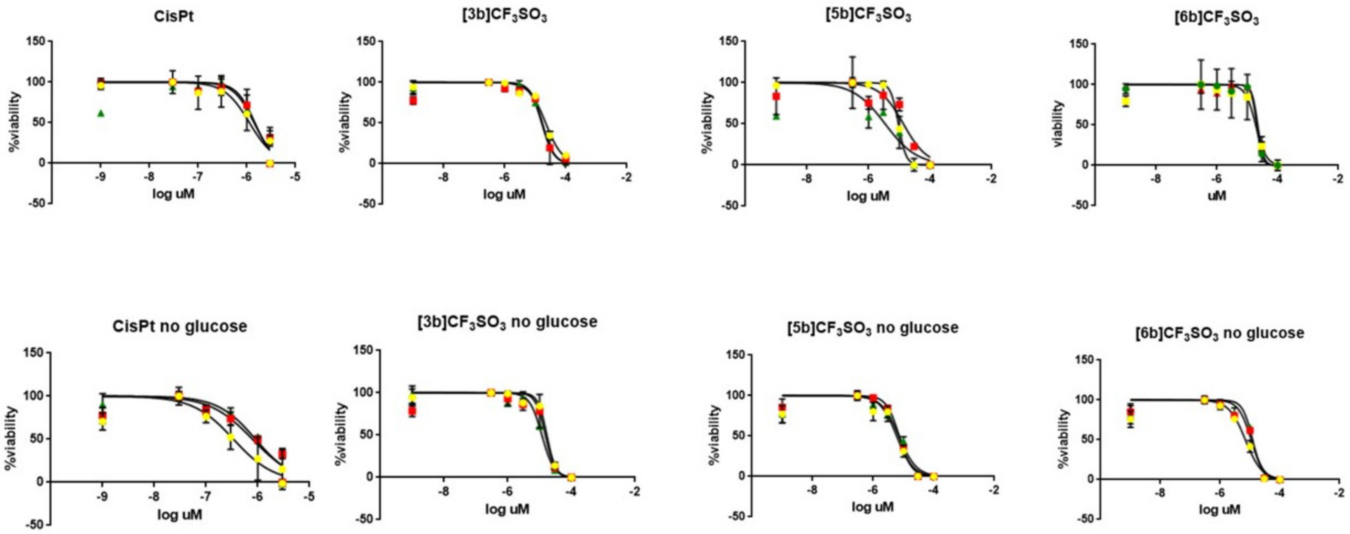

Supplement: Supplementary file 1 — om1c00519_si_001.pdf [file om1c00519_si_001.pdf]
